# Uncarialines A-E, new alkaloids from *Uncaria rhynchophylla* and their anticoagulant activity

**DOI:** 10.1007/s13659-023-00377-0

**Published:** 2023-04-12

**Authors:** Ke-Pu Huang, Li-Li Xu, Sheng Li, Yin-Ling Wei, Lian Yang, Xiao-Jiang Hao, Hong-Ping He, Yu Zhang

**Affiliations:** 1 School of Chinese Materia Medica, Yunnan University of Chinese Medicine, Kunming, 650500 People’s Republic of China; 2 State Key Laboratory of Phytochemistry and Plant Resources in West China, Kunming Institute of Botany, Chinese Academy of Sciences, Kunming, 650201 People’s Republic of China

**Keywords:** *Uncaria rhynchophylla*, Monoterpene indole alkaloids, Uncarialines A-E, Anticoagulant activity

## Abstract

Uncarialines A-E (**1–5**), five undescribed monoterpene indole alkaloids, together with five known analogues were obtained from the stems of *Uncaria rhynchophylla*. Alkaloids **1**–**3** were unique 3,4-seco-tricyclic alkaloids with a 6/5/10 ring system, while **4** and **5** possessed a rare rearranged scaffold originated from corynantheine-type alkaloids with C-2/C-7 oxidation. Their structures were characterized by a comprehensive analysis of MS, NMR, and ECD. Their effects on blood clotting times of human plasma were evaluated and alkaloid **5** had a slight prolongation effect on both thrombin time and activated partial thromboplastin time (*p* < 0.001).

**Graphical Abstract:**

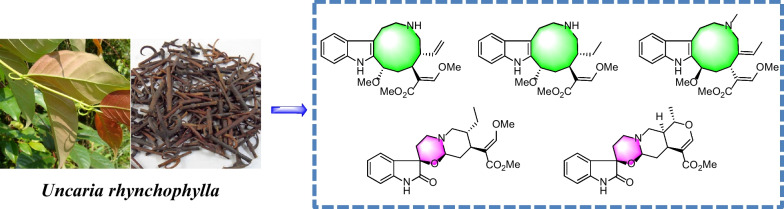

**Supplementary Information:**

The online version contains supplementary material available at 10.1007/s13659-023-00377-0.

## Introduction

Monoterpene indole alkaloids (MIAs) are a class of intriguing natural products, which are characterized by structural diversity and promising bioactivities [1–5]. The genus *Uncaria* (Rubiaceae family) is widely available in tropical regions and there are 14 species in southeast China [1, 6]. The genus *Uncaria* is enriched with MIAs with anticoagulant [7–9], anti-hypertensive [10], anti-Alzheimer’s disease [11], anti-inflammatory [12] and sedative effect [13]. *Uncaria rhynchophylla* (named “Gou Teng”) was conventionally used for treatment of cardiovascular and cerebrovascular diseases [14]. Interestingly, recent studies have shown that rhynchophylline and isorhynchophylline have anticoagulant effects that delay thrombosis [8, 9]. Besides, many novel MIAs with structural complexity have been characterized from *U. rhynchophylla* [15–19]. In order to discover structurally novel and biologically active MIAs, uncarialines A-E (**1–5**), five undescribed MIAs, as well as five known analogues, namely uncarialins D-G (**6–9**) and dihydrocorynantheine (**10**) were isolated from the stems of *U. rhynchophylla* [20, 21]. Presented herein are the isolation, identification, biosynthesis pathway and the anticoagulant activity of uncarialines A-E (**1–5**).

## Results and discussion

### Structure elucidation of the compounds

**Fig. 1:**
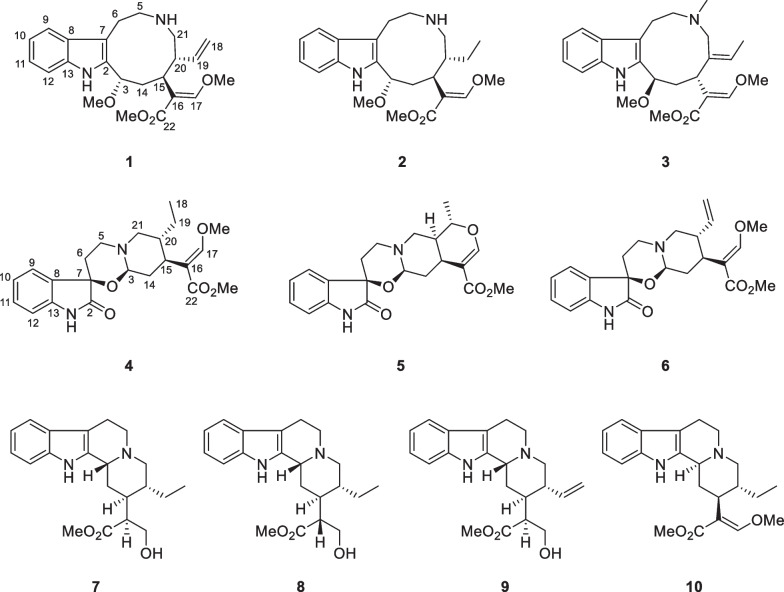
Molecular structures of compounds **1**–**10**

Compound **1** was isolated as a pale-yellow solid. The molecular formula of C_23_H_30_N_2_O_4_ was established by HRESIMS data (found: *m*/*z* 399.2273 [M + H]^+^, calcd for 399.2278). The observation of IR absorptions at 3423 and 1701 cm^−1^ implied the existence of amino and ester carbonyl, respectively. NMR spectral data (Tables 1 and 2) demonstrated the existence of an indole moiety (*δ*_C_ 134.6, 112.1, 127.5, 118.9, 119.2, 122.2, 110.9, 136.5), a *β*-methoxyacrylate methyl ester moiety [*δ*_H_ 7.11 (1H, H-17), 3.61 (3H, 17-OMe), 3.59 (3H, 22-OMe); *δ*_C_ 113.8 (C-16), 158.7 (C-17), 168.5 (C-22), 61.2 (17-OMe), 51.0 (22-OMe)], and a vinyl group [*δ*_H_ 4.82 (1H, H-18b), 4.89 (1H, H-18a), 5.49 (1H, H-19), 2.76 (1H, H-20); *δ*_C_ 114.2 (C-18), 140.2 (C-19), 46.9 (C-20)]. The key ^1^H-^1^H COSY cross-peaks of H-3/H-14, H-14/H-15, H-15/H-20, H-20/H-21, H-5/H-6 and HMBC correlations of H-21 (*δ*_H_ 2.59, 2.87) to C-5 (*δ*_C_ 45.8), H-6 (*δ*_H_ 2.94, 3.10) to C-2 (*δ*_C_ 134.6), H-3 (*δ*_H_ 4.79) to C-7 (*δ*_C_ 112.1) indicated the existence of indole-azecane fused heterocycles. Meanwhile, HMBC correlations from H-17 (*δ*_H_ 7.11) to C-15 (*δ*_C_ 31.2) implied that the *β*-methoxyacrylate methyl ester moiety was linked to C-15. The assignment of a vinyl moiety attached to C-20 was verified by ^1^H-^1^H COSY correlations of H-18/H-19/H-20. HMBC correlation of 3-OMe (*δ*_H_ 3.18) to C-3 (*δ*_C_ 75.0) implied that the methoxy group was attached to C-3. The planar structure of uncarialine A was thereby finally established (Fig. 1). The ROESY correlation of H-3 and H-20 indicated both protons were *β*-oriented, and thus H-15 took *α*-orientation due to the steric hindrance of the azecane ring. Moreover, the only ROESY correlations of H-17 with 17-OMe established (*E*)-configuration of the Δ^16(17)^ double bond (Fig. 3). The calculated ECD data of (3*S*, 15*S*, 20*R*)-**1** was compatible with its experimental data indicating the correct assignment of the absolute configuration of uncarialine A (**1**) (Fig. 4, Supplementary file).

**Table 1:** ^1^H NMR spectroscopic data for compounds **1–5** (*δ* in ppm, *J* in Hz)

NO	1^a^	2^a^	3^a^	4^b^	5^a^
3	4.79 (m)	4.78 (dd, 10.5, 6.5)	4.81 (d, 8.0)	4.74 (d, 9.0)	4.77 (dd, 10.0, 3.5)
5a	3.26 (td, 13.0, 5.0)	3.39 (m)	3.17 (m)	3.13 (m)	3.08 (td, 14.0, 2.5)
5b	2.89 (m)	2.93 (m)	2.33 (m)	2.88 (d, 5.4)	2.73 (m)
6a	3.10 (td, 13.0, 5.0)	3.10 (td, 14.0, 6.0)	3.10 (td, 6.5, 3.0)	2.50 (m)	2.38 (td,14.0, 5.0)
6b	2.94 (dd, 14.5, 5.0)	2.97 (m)	2.81 (m)	1.86 (d, 14.4)	1.81 (dt, 14.0, 2.5)
9	7.58 (d, 8.0)	7.56 (d, 8.0)	7.53 (d, 8.0)	7.38 (d, 7.8)	7.35 (d, 7.5)
10	7.12 (t, 8.0)	7.10 (t, 8.0)	7.10 (td, 8.0, 1.5)	7.05 (t, 7.8)	7.04 (td, 7.5, 1.0)
11	7.20 (t, 8.0)	7.19 (t, 8.0)	7.15 (td, 8.0, 1.5)	7.22 (t, 7.8)	7.23 (td, 7.5, 1.0)
12	7.36 (d, 8.0)	7.34 (d, 8.0)	7.34 (d, 8.0)	6.81 (d, 7.8)	6.80 (d, 7.5)
14a	2.84 (m)	2.86 (ddd, 14.0, 12.0, 6.5)	3.13 (m)	2.04 (m)	2.15 (t, 3.5)
14b	1.82 (t, 10.0)	1.78 (t, 12.0)	1.70 (ddd, 14.5, 8.0, 2.5)	1.62 (m)	1.42 (m)
15	2.61 (d, 10.0)	2.44 (m)	2.61 (dd, 12.0, 2.5)	2.61 (m)	2.70 (m)
17	7.11 (s)	7.18 (s)	7.27 (s)	7.32 (s)	7.54 (s)
18a	4.89 (d, 17.0)	0.86 (d, 3.5)	1.76 (d, 5.5)	0.84 (t, 7.8)	1.40 (m)
18b	4.82 (m)				
19a	5.49 (dt,17.0, 10.0)	1.35 (q, 8.5)	5.47 (q, 7.0)	1.39 (m)	4.56 (dq, 12.5, 6.0)
19b		0.85 (m)		0.99 (m)	
20	2.76 (m)	1.89 (m)		2.21 (d, 9.6)	1.61 (m)
21a	2.87 (m)	2.75 (m)	3.57 (d, 12.5)	2.99 (m)	2.91 (dd, 12.5, 2.0)
21b	2.59 (m)	2.55 (dd, 13.0, 7.0)	2.62 (d, 12.5)	2.04 (m)	2.65 (dd, 12.5, 3.5)
N-Me			2.16 (s)		
3-OMe	3.18 (s)	3.20 (s)	3.35 (s)		
17-OMe	3.61 (s)	3.60 (s)	3.73 (s)	3.77 (s)	
22-OMe	3.59 (s)	3.58 (s)	3.65 (s)	3.67 (s)	3.65 (s)

^a^500 MHz in CDCl_3_; ^b^600 MHz in CD_3_OD

**Table 2:** ^13^C NMR spectroscopic data for compounds **1–5** (*δ* in ppm)

No	1^a^	2^a^	3^a^	4^b^	5^a^
2	134.6, s	134.8, s	134.5, s	178.1, s	178.1, s
3	75.0, d	75.4, d	78.0, d	86.9, d	86.5, d
5	45.8, t	46.2, t	55.0, t	48.1, t	48.0, t
6	23.0, t	23.8, t	23.1, t	31.7, t	31.6, t
7	112.1, s	111.4, s	109.3, s	74.7, s	74.7, s
8	127.5, s	127.5, s	129.8, s	130.9, s	130.8, s
9	118.9, d	119.0, d	117.9, d	124.5, d	124.4, d
10	119.2, d	119.3, d	118.7, d	123.2, d	123.1, d
11	122.2, d	122.3, d	120.9, d	129.7, d	129.8, d
12	110.9, d	110.9, d	110.5, d	109.5, d	109.6, d
13	136.5, s	136.4, s	137.8, s	139.9, s	139.9, s
14	39.4, t	39.1, t	40.8, t	35.5, t	35.6, t
15	31.2, d	32.4, d	34.8, d	36.5, d	30.2, d
16	113.8, s	113.0, s	113.5, s	111.3, s	109.7, s
17	158.7, d	159.3, d	159.0, d	160.1, d	155.4, d
18	114.2, t	12.4, q	13.5, q	11.4, q	18.6, q
19	140.2, d	21.9, t	127.9, d	23.9, t	72.0, d
20	46.9, d	42.7, d	138.1, s	38.7, d	37.9, d
21	49.5, t	46.2, t	65.0, t	58.5, t	53.7, t
22	168.5, s	168.7, s	168.8, s	169.3, s	167.7, s
N-Me			40.5, q		
3-OMe	56.5, q	56.7, q	58.1, q		
17-OMe	61.2, q	61.3, q	61.5, q	61.5, q	61.5, q
22-OMe	51.0, q	51.2, q	51.2, q	51.3, q	51.0, q

^a^125 MHz in CDCl_3_; ^b^150 MHz in CD_3_OD

**Fig. 2:**
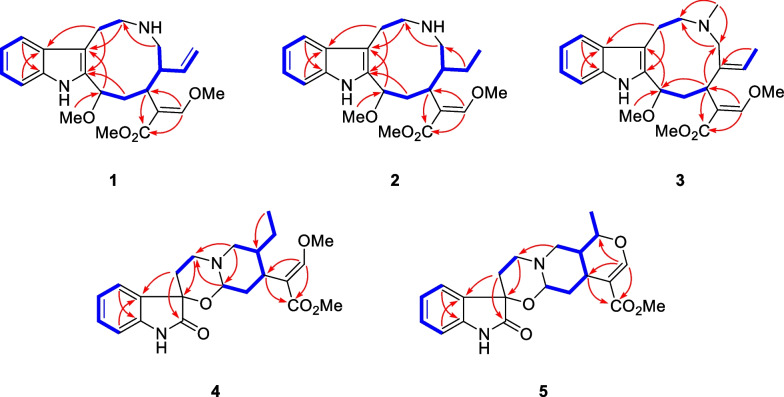
Key HMBC (arrow) and ^1^H-^1^H COSY (bold) correlations of uncarialines A-E (**1**–**5**)

**Fig. 3:**
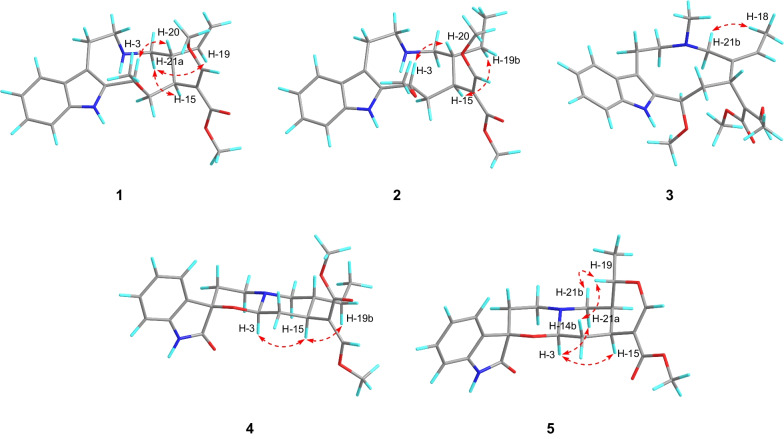
Key ROESY correlations of uncarialines A-E (**1**–**5**)

**Fig. 4:**
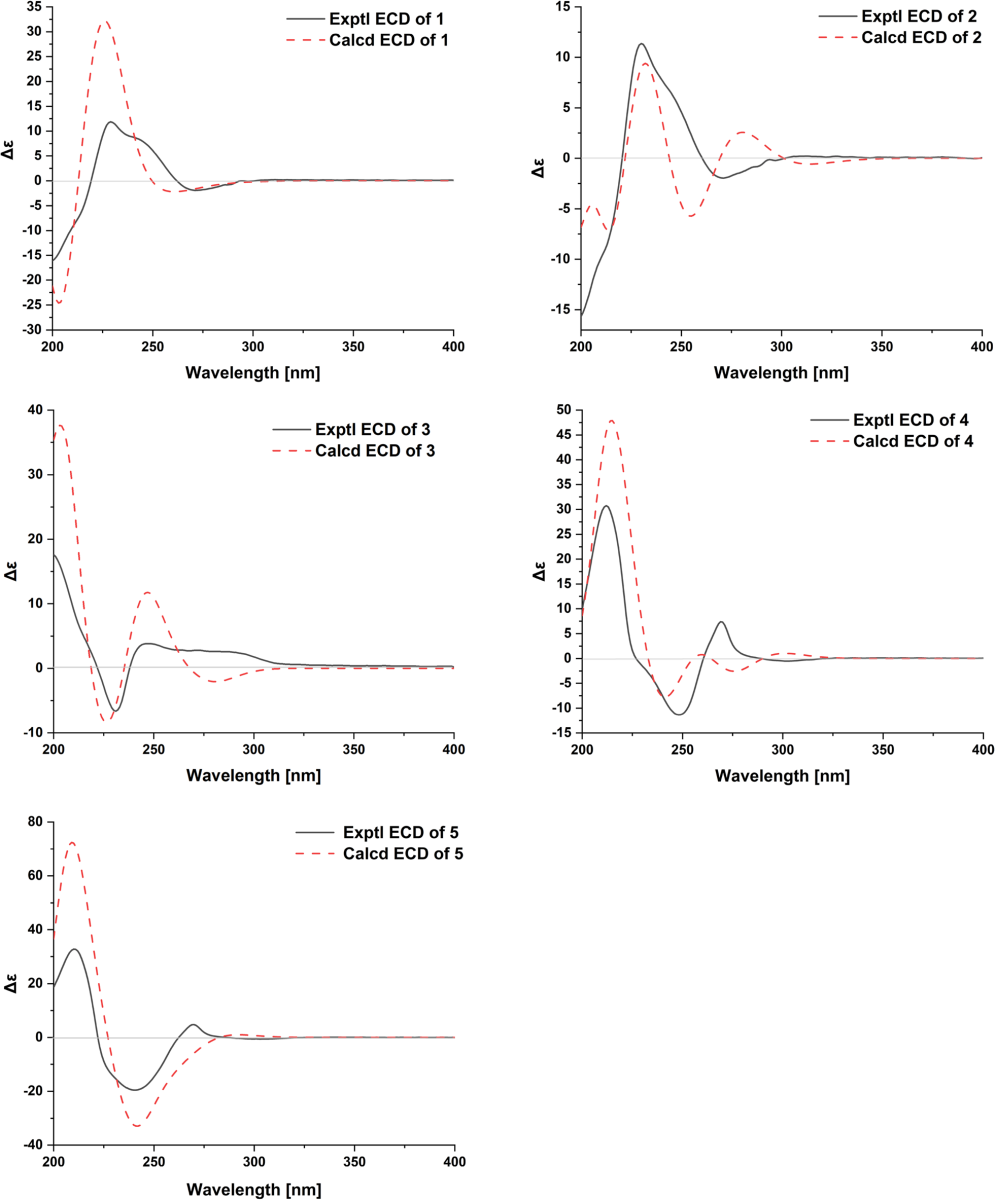
Experimental and calculated ECD of uncarialines A-E (**1**–**5**)

Compound **2** was isolated as a pale-yellow solid. It had a molecular formula of C_23_H_32_N_2_O_4_ in terms of HRESIMS ion at *m*/*z* 401.2441 ([M + H]^+^, calcd for 401.2435). The NMR data of **2** indicated that **2** had the same basic scaffold as that of **1** (Tables 1 and 2). The distinct observations were the presence of an ethyl group (*δ*_H_ 0.86, *δ*_C_ 12.4; *δ*_H_ 0.85, 1.35, *δ*_C_ 21.9) in **2**. The molecular weight of **2** has two more mass units and one less unsaturation than that of **1**, demonstrating **2** was the Δ^18(19)^ double bond reduction form of **1**. The structure of uncarialine B was thereby established (Fig. 1), which was further verified by HMBC and ^1^H-^1^H COSY spectra analysis (Fig. 2). The identical ROESY and ECD spectra of uncarialine B (**2**) and alkaloid **1** demonstrated both alkaloids had the same relative and absolute configurations (Figs. 3 and 4).

Compound **3** was isolated as a pale-yellow solid. It had the molecular formula of C_24_H_32_N_2_O_4_ by HRESIMS analysis (found: *m*/*z* 413.2443 [M + H]^+^, calcd for 413.2435) with 14 mass units larger than that of **1**. The NMR data of **3** indicated that** 3** had the same basic scaffold as that of **1** (Tables 1 and 2), with the existence of a unique signal with *N*-methyl [*δ*_H_ 2.16 (3H, s, *N*-Me); *δ*_C_ 40.5 (*N*-Me)], and an allyl group [*δ*_H_ 1.76 (3H, H-18), 5.47 (1H, H-19); *δ*_C_ 13.5 (C-18), 127.9 (C-19), 138.1 (C-20)]. HMBC correlation of *N*-Me (*δ*_H_ 2.16) with C-5 (*δ*_C_ 55.0) and C-21 (*δ*_C_ 65.0) indicated that the methyl group was linked to *N*-4. Moreover, the allyl attached to C-20 due to the HMBC correlations of H-18 (*δ*_H_ 1.76) to C-20 (*δ*_C_ 138.1). Thus, the structure of uncarialine C was thereby established (Fig. 1). The ROESY cross-peaks of H-18 with H-21b and of H-17 with 17-OMe, confirmed (*Z*)- and (*E*)-configurations of Δ^19(20)^ and Δ^16(17)^ double bonds, respectively. The deficiency of ROESY correlation of H-15 with H-3 confirmed that the methoxy group (C-3) and the *β*-methoxyacrylate methyl ester moiety (C-15) were opposite. Thus, there are two possible stereoisomers (3R*,15R*)-**3** or (3S*,15S*)-**3**. The absolute stereochemistry of (3*R*,15*R*)-**3** was assigned finally by the compatible calculated and experimental ECD spectra of uncarialine C (**3**) (Fig. 4).

Compound **4** was isolated as a white solid. It had the molecular formula of C_22_H_28_N_2_O_5_, as evidenced by HRESIMS ion at *m/z* 401.2076 ([M + H]^+^, calcd for 401.2071). The maxima UV absorptions at 207, 242, and 295 nm demonstrated an oxindole chromophore [22]. IR absorptions showed the existence of amide carbonyl (1625 cm^−1^), ester carbonyl (1708 cm^−1^), and amino group (3423 cm^−1^). ^13^C NMR spectroscopy suggested that **4** had 22 carbons and had a high similarity with uncarialin D [20], except for the terminal vinyl group in uncarialin D was reduced to ethyl group in **4**. Meanwhile, the ROESY correlations from H-15 to H-3 and H-19b indicated H-3 and H-15 were *α*-oriented while H-20 was *β*-oriented. Moreover, the only ROESY correlation of H-17 with 17-OMe in ROESY spectrum indicated the Δ^16(17)^ double bond took (*E*)-configuration. The Cotton effects at 212, 269, and 248 nm suggested the absolute configuration of (3*R*,7*R*,15*S*,20*R*)-**4** [23, 24], which was confirmed by the ECD calculation of uncarialine D (**4**) (Fig. 4).

Compound **5** was isolated as a white solid. It had a molecular formula of C_21_H_24_N_2_O_5_, as given by HRESIMS analysis (found: m/z 385.1758 [M + H]^+^; calcd for 385.1758). The IR absorptions implied the existence of amino group (3422 cm^−1^), ester carbonyl (1709 cm^−1^) and amide carbonyl (1626 cm^−1^). Interpretation of its NMR data suggested **5** had a similarity with melodinoxanine [25]. The major difference was that **5** lacked two aromatic methoxy groups, which was further verified by the key ^1^H-^1^H COSY cross-peaks of H-9/H-10, H-10/H-11, and H-11/H-12. The ROESY correlations of H-3 with H-15, and H-21a confirmed that they were assigned as *α*-oriented. Thus, the ROESY cross-peaks of H-19 with H-14 and H-21b implied H-19 took *β*-orientation. The coupling constant (*J*_19,20_ = 12.5 Hz) between H-19 and H-20 in the ^1^H NMR spectrum confirmed H-20 took *α*-orientation. Additionally, the only ROESY cross-peaks of H-17 with 17-OMe indicated (*E*)-configuration of the Δ^16(17)^ double bond. The absolute stereochemistry of uncarialine E (**5**) was finally characterized by the ECD calculation result of (3*R*,7*R*,15*S*,19*S*,20*S*)-**5** identical with the corresponding experimental ECD data (Fig. 4).

**Scheme 1.**
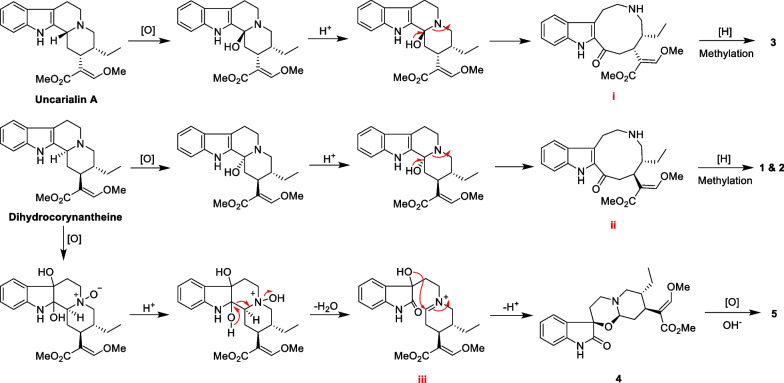
Hypothesis biogenetic pathway for uncarialines A-E (**1**–**5**)

The possible biogenetic routes for **1–5** is presented in Scheme 1. Biogenetically, the H-3 of uncarialin A or dihydrocorynantheine (**10**) was initially oxidized to hydroxyl group, and then the hydroxyl derivatives undergo hydrolysis of tertiary amine under acidic conditions to yield intermediates **i** and **ii**, which eventually undergoes reduction, oxidation, and methylation to form compounds **1–3**, respectively. Likewise, dihydrocorynantheine (**10**) was oxidized to 2,7-dihydroxy-dihydrocorynantheine *N*-oxide, followed by hydrolysis of quaternary ammonium under acidic conditions to form the key intermediate **iii**, which finally undergoes rearrangement reaction to form compounds **4** and **5**.

### Anticoagulant activity

The anticoagulant activity of the new isolates is represented by the following parameters: thrombin time (TT), prothrombin time (PT) and activated partial thromboplastin time (APTT) [26]. Compounds **1**–**4** were inactive on TT, PT and APTT (*p* > 0.05), while compound **5** had a slightly prolongation effect on both TT and APTT (*p* < 0.001) (Table 3).

**Table 3:** Determination of the effects of compounds on blood clotting times of human plasma

Compounds	Concentration	TT (s)	PT (s)	APTT (s)
Control plasma	–	13.6 ± 0.40	13.1 ± 0.17	44.9 ± 0.84
**1**	200 μM	13.6 ± 0.36	13.3 ± 0.17	44.9 ± 0.55
**2**	200 μM	13.9 ± 0.64	13.5 ± 0.32	46.0 ± 0.47
**3**	200 μM	13.6 ± 026	13.3 ± 0.17	45.3 ± 0.32
**4**	200 μM	13.7 ± 0.65	13.1 ± 0.21	45.1 ± 0.38
**5**	200 μM	19.5 ± 0.91***	14.5 ± 0.21	53.0 ± 0.62***
HEP^a^	16 μg/mL	–	24.5 ± 0.49***	–
LMWH^*b*^	0.89 μM	47.5 ± 2.28***	–	183.3 ± 2.49***

***p < 0.001; n = 3

^a^Positive control of PT; ^b^Positive control of APTT and TT

## Experimental

### General experimental procedures

The experimental apparatus is as previously reported [2, 3]

### Plant material

The stems of *U. rhynchophylla* were obtained in Jianhe, Guizhou Province, China, on May 2020 and identified by Prof. Hongping He, one of our co-authors. The sample specimen (No. Z20200520) was deposited at Kunming Institute of Botany.

### Extraction and isolation

The crushed stems of *U. rhynchophylla* were cold soaked 3 times in methanol (MeOH) to obtain the extract. The crude alkaloids (2030 g) were obtained by the previously procedures [2, 3], which were divided to six fractions (A-F) using silica gel column chromatography (DCM/MeOH, 49:1, 29:1, 9:1, 1:1, v/v). Among them, fraction B (18 g) was divided into three fractions (B_1_–B_3_) by a silica gel column (300–400 mesh, DCM/MeOH, 49:1, 29:1, 9:1, 1:1, v/v). Fraction B_2_ (1.8 g) was separated by HPLC with MeCN/H_2_O (60:40, 0.01% Et_2_NH, 3 mL/min) to give **5** (13 mg, t_*R*_ 15.0 min) and **10** (47 mg, t_*R*_ 21.0 min). Fraction C (96 g) was divided to seven fractions (C_1_-C_7_) by a silica gel column (DCM/MeOH, 49:1, 19:1, 9:1, 1:1, v/v). Fraction C_3_ (4.2 g) was separated by RP-C18 (MeOH/H_2_O, 30:70, 50:50, 100:0, v/v) and HPLC with MeCN/H_2_O (52:48, 0.01% Et_2_NH, 3 mL/min) to give **4** (5 mg, t_*R*_ 11.0 min) and **6** (7 mg, t_*R*_ 28.0 min). Fraction E (184 g) was divided to nine fractions (E_1_-E_9_) by silica gel column chromatography (DCM/MeOH, 19:1, 9:1, 1:1, v/v). Fraction E_2_ (520 mg) was separated by Sephadex LH-20 (MeOH) and HPLC with MeCN/H_2_O (45:55, 0.01% Et_2_NH, 3 mL/min) to obtain **1** (9 mg, t_*R*_ 9.0 min) and **2** (11 mg, t_*R*_ 18.0 min). Fraction E_3_ (210 mg) was further separated by HPLC with MeCN/H_2_O (36:64, 0.01% Et_2_NH, 3 mL/min) to give **7** (26 mg, t_*R*_ 13.0 min), **8** (34 mg, t_*R*_ 20.5 min) and **9** (33 mg, t_*R*_ 27.0 min). Fraction E_5_ (2.5 g) was separated by Sephadex LH-20 and subsequent HPLC separation with MeCN/H_2_O (30:70, 0.01% Et_2_NH, 3 mL/min) to obtain **3** (43 mg, t_*R*_ 38.0 min).

### Uncarialine A (1)

Uncarialine A (**1**): pale-yellow solid; ${[\alpha]^{22}_{\text{D}}}$ − 8 (*c* 0.3, MeOH); UV (MeOH) *λ*max (log *ε*): 223 (3.4) nm; ECD (0.0034 M, MeOH) λmax (∆*ε*) 230 (+ 11.9), 271 (− 1.9); IR (KBr) *v*max 3423, 2922, 2852, 1701, 1634, 1461, 1244, 1116 cm^−1^; ^1^H and ^13^C NMR data (CDCl3, 500 and 125 MHz) see Tables 1 and 2; HRESIMS *m/z* 399.2273 [M + H]^+^ (calcd for C23H31N2O4, 399.2278).

### Uncarialine B (2)

Uncarialine B (**2**): pale-yellow solid; ${[\alpha]^{22}_{\text{D}}}$ − 7 (*c* 0.3, MeOH); UV (MeOH) *λ*max (log *ε*): 223 (3.3) nm; ECD (0.0048 M, MeOH) λmax (∆*ε*) 230 (+ 11.4), 271 (− 2.0); IR (KBr) *v*max 3429, 2922, 2852, 1701, 1632, 1461, 1244, 1106 cm^−1^; ^1^H and ^13^C NMR data (CDCl3, 500 and 125 MHz) see Tables 1 and 2; HRESIMS *m/z* 401.2441 [M + H]^+^ (calcd for C23H33N2O4, 401.2435).

### Uncarialine C (3)

Uncarialine C (**3**): pale-yellow solid; ${[\alpha]^{22}_{\text{D}}}$+ 184 (*c* 0.1, MeOH); UV (MeOH) *λ*max (log *ε*): 228 (3.6) nm; ECD (0.0023 M, MeOH) λmax (∆*ε*) 199 (+ 17.6), 230 (− 6.7), 247 (+ 3.8), 287 (+ 2.6); IR (KBr) *v*max 3420, 2938, 1705, 1634, 1461, 1236, 1093 cm^−1^; ^1^H and ^13^C NMR data (CDCl3, 500 and 125 MHz) see Tables 1 and 2; HRESIMS *m/z* 413.2443 [M + H]^+^ (calcd for C24H33N2O4, 413.2435).

### Uncarialine D (4)

Uncarialine D (**4**): white solid; ${[\alpha]^{22}_{\text{D}}}$ + 7 (*c* 0.2, MeOH); UV (MeOH) *λ*max (log *ε*): 207 (3.6) nm; ECD (0.0021 M, MeOH) λmax (∆*ε*) 212 (+ 30.8), 248 (− 11.4), 269 (+ 7.4); IR (KBr) *v*max 3423, 2924, 2853, 1708, 1625, 1470, 1247, 1108 cm^−1^; ^1^H and ^13^C NMR data (CD_3_OD, 600 and 150 MHz) see Tables 1 and 2; HRESIMS *m/z* 401.2076 [M + H]^+^ (calcd for C22H29N2O5, 401.2071).

### Uncarialine E (5)

Uncarialine E (**5**): white solid; ${[\alpha]^{22}_{\text{D}}}$ − 32 (*c* 0.1, MeOH); UV (MeOH) *λ*max (log *ε*): 208 (4.3) nm; ECD (0.0003 M, MeOH) λmax (∆*ε*) 210 (+ 32.8), 241 (− 19.6), 270 (+ 4.8); IR (KBr) *v*max 3422, 2927, 2854, 1709, 1626, 1472, 1211, 1100 cm^−1^; ^1^H and ^13^C NMR data (CDCl3, 500 and 125 MHz) see Tables 1 and 2; HRESIMS *m/z* 385.1758 [M + H]^+^ (calcd for C21H25N2O5, 385.1758).

### Blood clotting times

Chemicals. Reagents of TT, PT and APTT, CaCl_2_ and coagulation control plasma were produced in TECO (Germany). Tris–HCl was purchased from Amresco (USA). Reference anticoagulant drug (heparin, HEP; low molecular weight heparin, LMWH) and DMSO were produced in Sigma-Aldrich (USA).

The measurements were taken using the MC-4000 Optic coagulometer (Germany). Prior to the detection, coagulation control plasma were pre-incubated with the examined compounds (15 min, 37 °C) at the final concentrations of 200 μM.

## Concluding remarks

In summary, 10 alkaloids including five new ones were obtained from the stems of *U. rhynchophylla*. Among them, uncarialines A-C (**1**–**3**) were unique 3,4-seco-tricyclic MIAs with a 6/5/10 ring system, while uncarialines D (**4**) and E (**5**) possessed a rare rearranged skeleton derived from corynantheine-type alkaloids with C-2/C-7 oxidation. It is noteworthy that the stereochemistry of **3** at C-3 and C-15 were opposite to those of **1** and **2** indicating the possibility of specific enzyme catalyze the formation of the corresponding chiral centers. The findings not only enrich the diversity of secondary metabolisms of *U. rhynchophylla*, but only provide insight into the complex biosynthetic mechanism of such alkaloids category.

## Supplementary Information

**Additional file 1.** HRESIMS, NMR, ECD, and IR spectra of compounds 1–5.
