# Correction: Uncarialines A-E, new alkaloids from *Uncaria rhynchophylla* and their anticoagulant activity

**DOI:** 10.1007/s13659-023-00378-z

**Published:** 2023-05-12

**Authors:** Ke‑Pu Huang, Li‑Li Xu, Sheng Li, Yin‑Ling Wei, Lian Yang, Xiao‑Jiang Hao, Hong‑Ping He, Yu Zhang

**Affiliations:** 1 School of Chinese Materia Medica, Yunnan University of Chinese Medicine, Kunming, 650500 People’s Republic of China; 2 State Key Laboratory of Phytochemistry and Plant Resources in West China, Kunming Institute of Botany, Chinese Academy of Sciences, Kunming, 650201 People’s Republic of China

**Correction** **:** **Natural Products and Bioprospecting (2023) 13: 13** **https://doi.org/10.1007/s13659-023-00377-0**

Following publication of the original article [1], it was reported that the original version of this article unfortunately contained a mistake. Prof. Hong-Ping He is also one of the corresponding authors.

The correct corresponding information should read:

Hong‑Ping He^1,*^

E-mail address: hehongping@yahoo.com

Yu Zhang^2,*^

E-mail address: zhangyu@mail.kib.ac.cn

The original article [1] has been updated.
