# Supplementary material for: Uncarialines A-E, new alkaloids from Uncaria rhynchophylla and their anticoagulant activity
Source: Nat Prod Bioprospect. 2023 Apr 12;13(1):13. doi: 10.1007/s13659-023-00377-0 (PMC10097851; doi:10.1007/s13659-023-00377-0)
Supplement: Supplementary file 1 — Additional file 1. HRESIMS, NMR, ECD, and IR spectra of compounds 1–5. [file 13659_2023_377_MOESM1_ESM.pdf]

Uncarialines A-E, new alkaloids from *Uncaria rhynchophylla* and their anticoagulant activity

Ke-Pu Huang [a,b], Li-Li Xu [b], Sheng Li [b], Yin-Ling Wei [b], Lian Yang [b], Xiao-Jiang Hao [b], Hong-Ping He [a,] * and Yu Zhang [b,] *

[a] *School of Chinese Materia Medica, Yunnan University of Chinese Medicine, Kunming 650500, PR China*

[b] *State Key Laboratory of Phytochemistry and Plant Resources in West China, Kunming Institute of Botany, Chinese Academy of Sciences, Kunming 650201, PR China*

*Corresponding authors. Tel./fax: +86-871-65223263.

E-mail addresses: zhangyu@mail.kib.ac.cn (Y. Zhang).

Supporting Information

Contents

1. Spectra of physico-chemical properties of **1-5**

Figure S1 ^{1}H NMR spectrum of uncarialine A (**1**) in $CDCl_3$

Figure S2 ^{13}C NMR spectrum of uncarialine A (**1**) in $CDCl_3$

Figure S3 HSQC spectrum of uncarialine A (**1**) in $CDCl_3$

Figure S4 ^{1}H-^{1}H COSY spectrum of uncarialine A (**1**) in $CDCl_3$

Figure S5 HMBC spectrum of uncarialine A (**1**) in $CDCl_3$

Figure S6 ROESY spectrum of uncarialine A (**1**) in $CDCl_3$

Figure S7 HRESIMS spectrum of uncarialine A (**1**)

Figure S8 IR spectrum of uncarialine A (**1**)

Figure S9 ECD spectrum of uncarialine A (**1**)

Figure S10 ^{1}H NMR spectrum of uncarialine B (**2**) in $CDCl_3$

Figure S11 ^{13}C NMR spectrum of uncarialine B (**2**) in $CDCl_3$

Figure S12 HSQC spectrum of uncarialine B (**2**) in $CDCl_3$

Figure S13 ^{1}H-^{1}H COSY spectrum of uncarialine B (**2**) in $CDCl_3$

Figure S14 HMBC spectrum of uncarialine B (**2**) in $CDCl_3$

Figure S15 ROESY spectrum of uncarialine B (**2**) in $CDCl_3$

Figure S16 HRESIMS spectrum of uncarialine B (**2**)

Figure S17 IR spectrum of uncarialine B (**2**)

Figure S18 ECD spectrum of uncarialine B (**2**)

Figure S19 ^{1}H NMR spectrum of uncarialine C (**3**) in $CDCl_3$

Figure S20 ^{13}C NMR spectrum of uncarialine C (**3**) in $CDCl_3$

Figure S21 HSQC spectrum of uncarialine C (**3**) in $CDCl_3$

Figure S22 ^{1}H-^{1}H COSY spectrum of uncarialine C (**3**) in $CDCl_3$

Figure S23 HMBC spectrum of uncarialine C (**3**) in $CDCl_3$

Figure S24 ROESY spectrum of uncarialine C (**3**) in $CDCl_3$

Figure S25 HRESIMS spectrum of uncarialine C (**3**)

Figure S26 IR spectrum of uncarialine C (**3**)

Figure S27 ECD spectrum of uncarialine C (**3**)

Figure S28 ^{1}H NMR spectrum of uncarialine D (**4**) in CD_3OD

Figure S29 ^{13}C NMR spectrum of uncarialine D (**4**) in CD_3OD

Figure S30 HSQC spectrum of uncarialine D (**4**) in CD_3OD

Figure S31 ^{1}H-^{1}H COSY spectrum of uncarialine D (**4**) in CD_3OD

Figure S32 HMBC spectrum of uncarialine D (**4**) in CD_3OD

Figure S33 ROESY spectrum of uncarialine D (**4**) in CD_3OD

Figure S34 HRESIMS spectrum of uncarialine D (**4**)

Figure S35 IR spectrum of uncarialine D (**4**)

Figure S36 ECD spectrum of uncarialine D (**4**)

Figure S37 ^{1}H NMR spectrum of uncarialine E (**5**) in $CDCl_3$

Figure S38 ^{13}C NMR spectrum of uncarialine E (**5**) in $CDCl_3$

Figure S39 HSQC spectrum of uncarialine E (**5**) in $CDCl_3$

Figure S40 ^{1}H-^{1}H COSY spectrum of uncarialine E (**5**) in $CDCl_3$

Figure S41 HMBC spectrum of uncarialine E (**5**) in $CDCl_3$

Figure S42 ROESY spectrum of uncarialine E (**5**) in $CDCl_3$

Figure S43 HRESIMS spectrum of uncarialine E (**5**)

Figure S44 IR spectrum of uncarialine E (**5**)

Figure S45 ECD spectrum of uncarialine E (**5**)

2. Computational methods for ECD calculation of **1-5**

Figure S1 ^{1}H NMR spectrum of uncarialine A (**1**) in $CDCl_3$

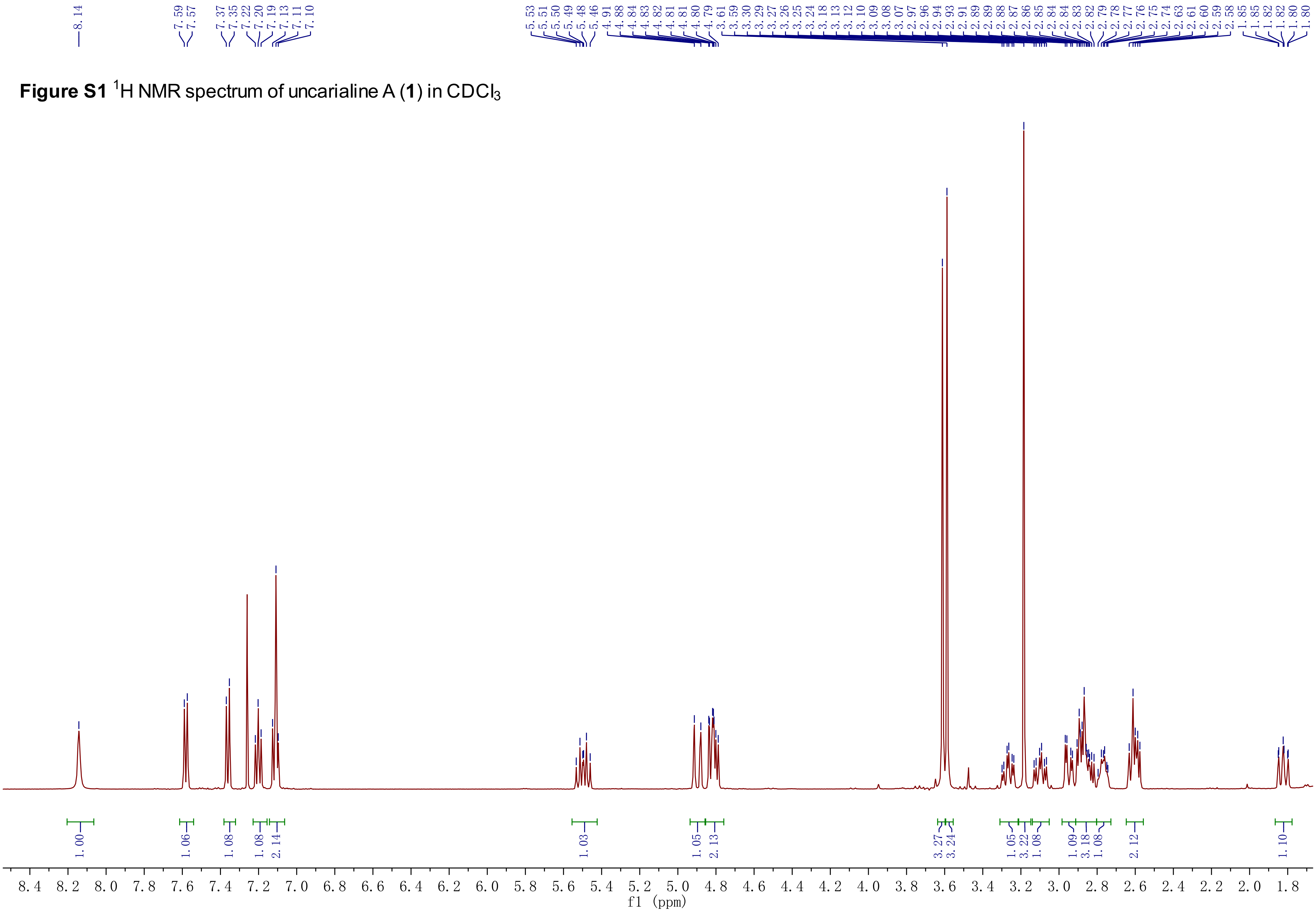

Figure S2 ^{13}C NMR spectrum of uncarialine A (**1**) in $CDCl_3$

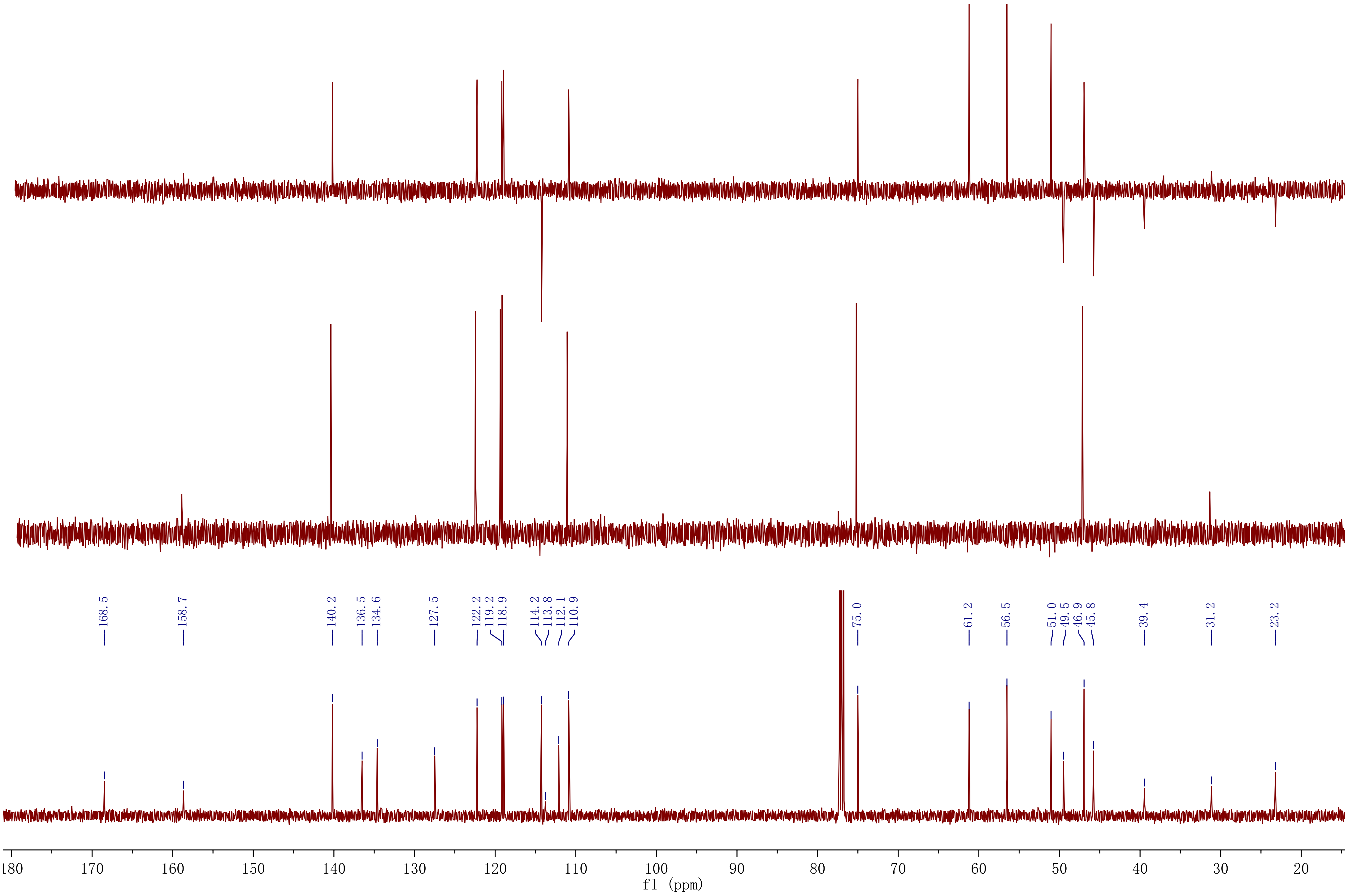

Figure S3 HSQC spectrum of uncarialine A (**1**) in $CDCl_3$

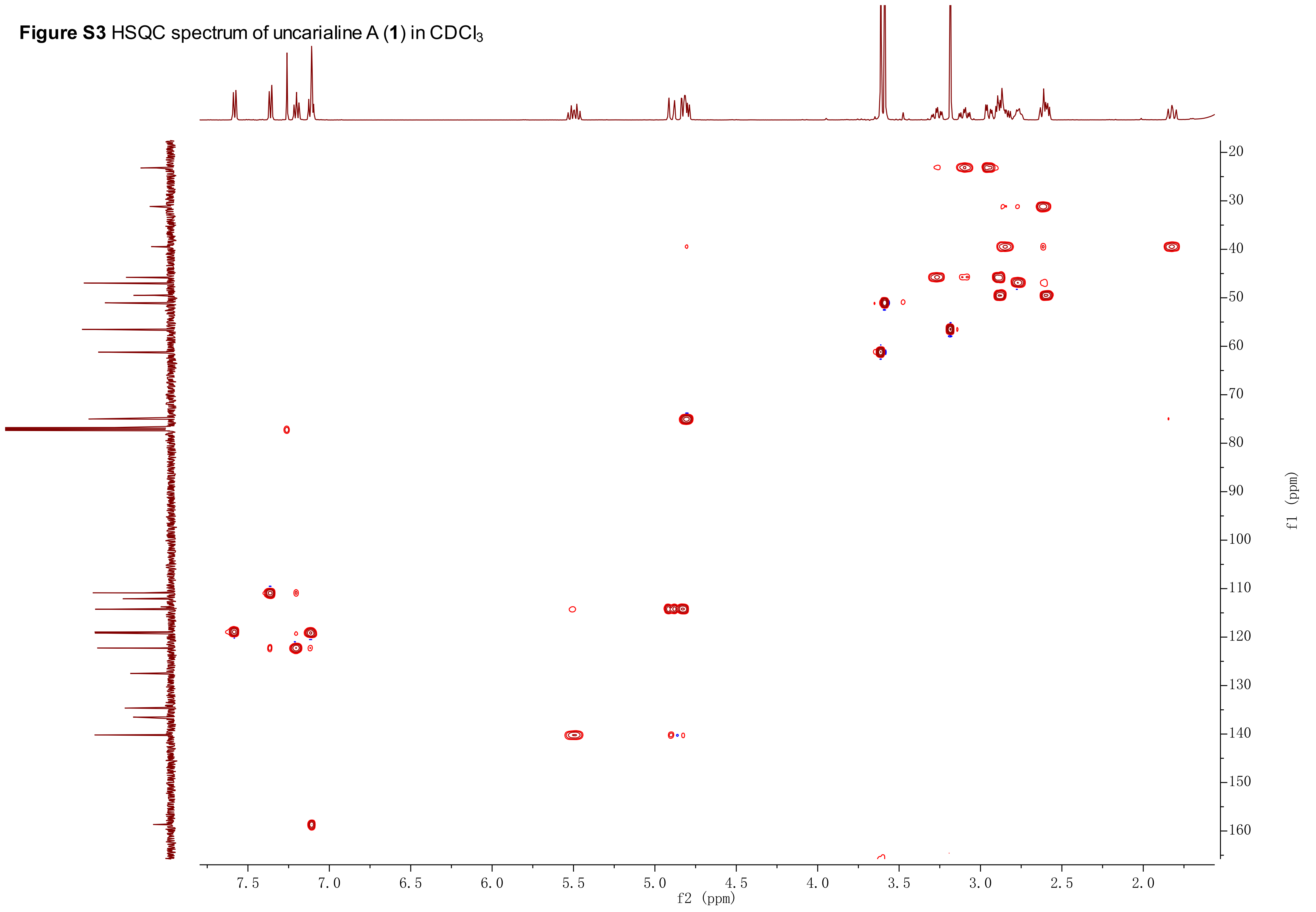

Figure S4 ^{1}H-^{1}H COSY spectrum of uncarialine A (**1**) in $CDCl_3$

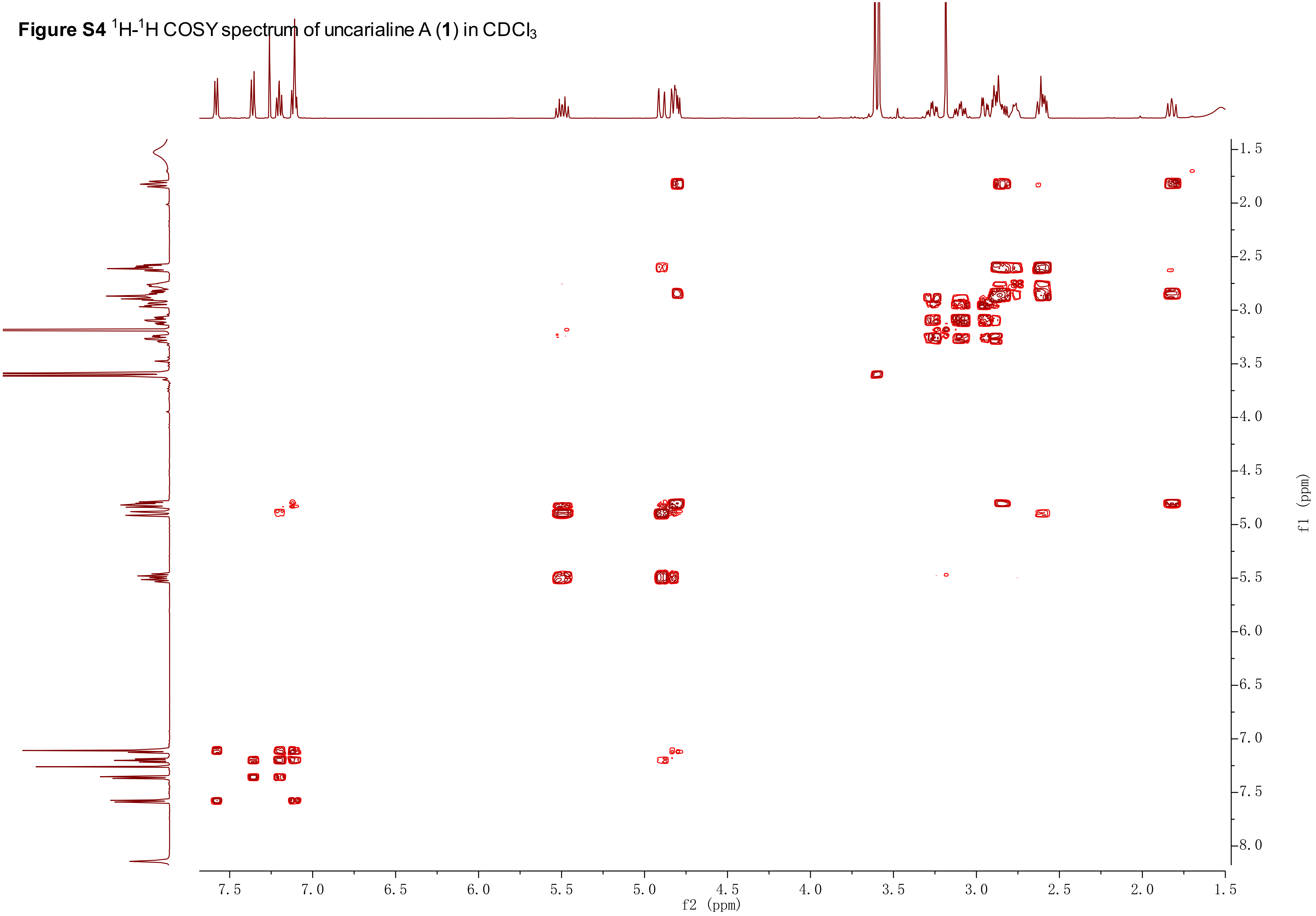

Figure S5 HMBC spectrum of uncarialine A (**1**) in $CDCl_3$

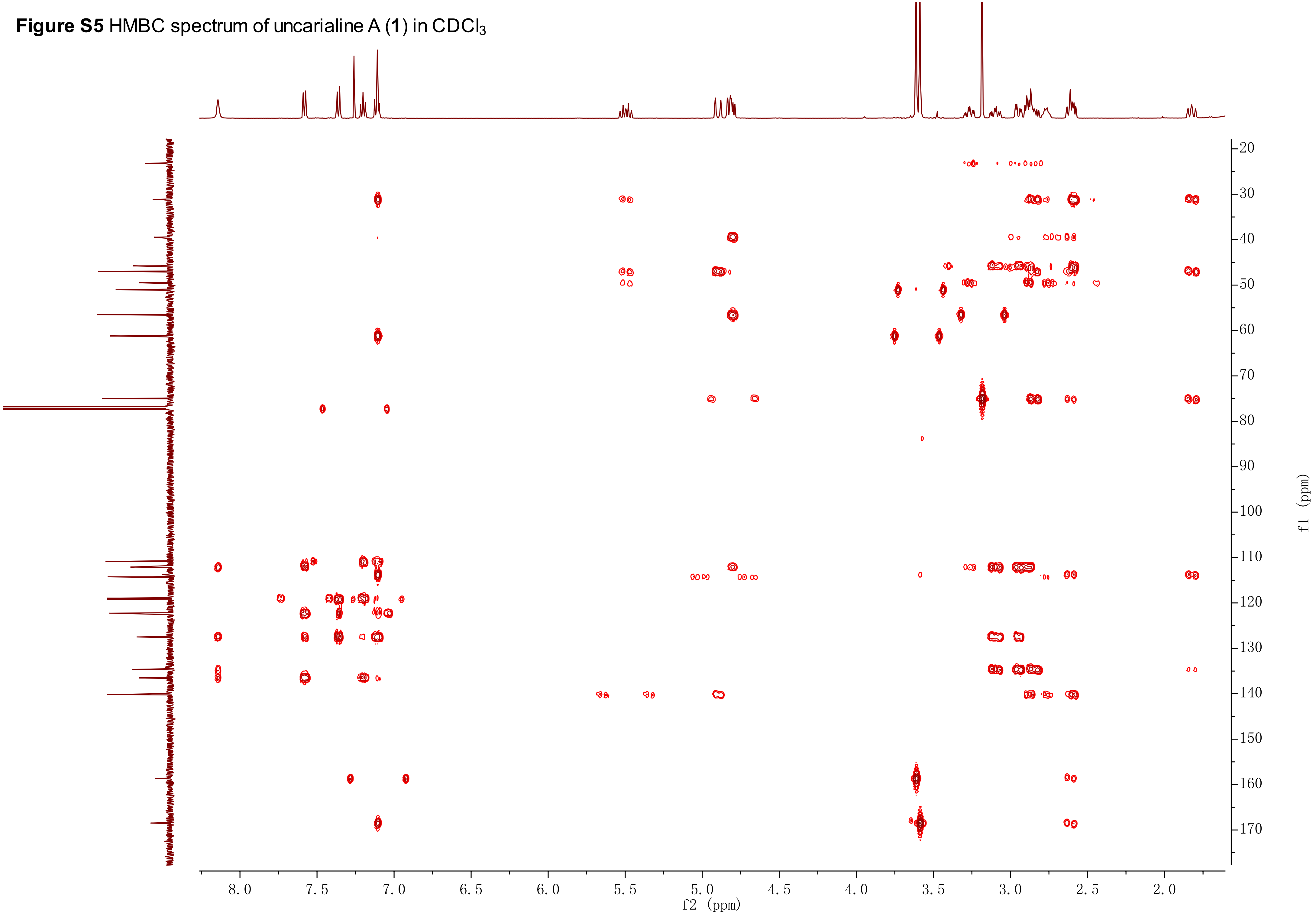

Figure S6 ROESY spectrum of uncarialine A (**1**) in $CDCl_3$

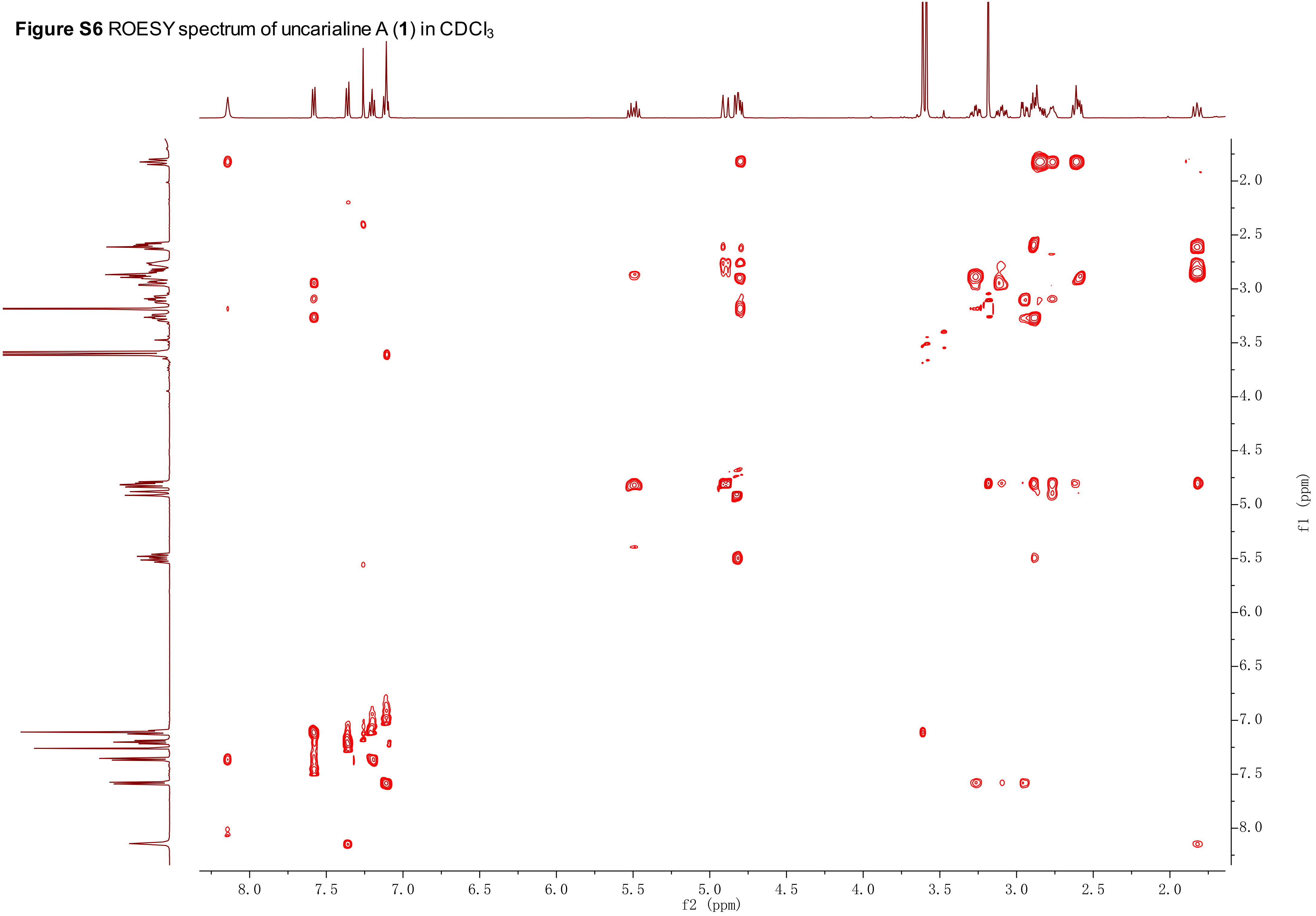

Figure S7 HRESIMS spectrum of uncarialine A (**1**)

Qualitative Analysis Report

**Data Filename**	HKP-22b.d	**Sample Name**	HKP-22b
**Sample Type**	Sample	**Position**	P1-A1
**Instrument Name**	Instrument 1	**User Name**	
**Acq Method**	s.m	**Acquired Time**	4/24/2022 9:29:46 AM
**IRM Calibration Status**	Success	**DA Method**	PCDL.m
**Comment**			

**Sample Group**		**Info.**
**Acquisition SW Version**	6200 series TOF/6500 series Q-TOF B.05.01 (B5125.2)	

User Spectra

Fragmentor Voltage	Collision Energy	Ionization Mode
135	0	ESI

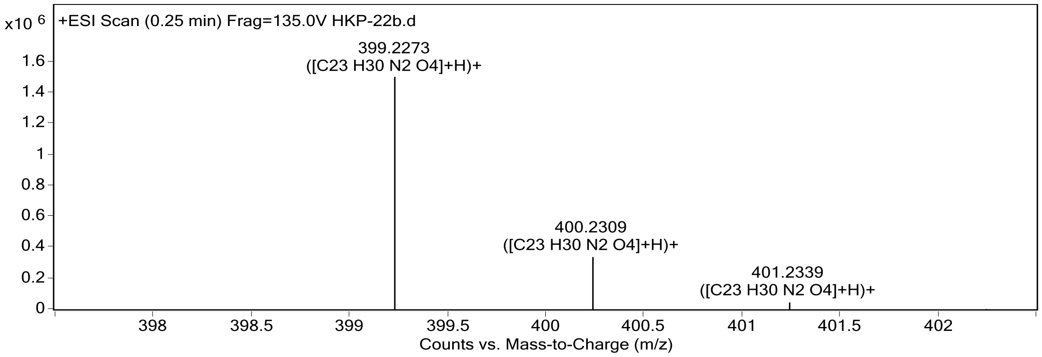

Peak List

m/z	z	Abund	Formula	Ion
84.9595	1	107531.23		
102.1272	1	712021.5		
103.1307	1	90982.04		
125.9858	1	111846.98		
158.0021	1	70535.51		
214.917	1	76117.55		
367.2012	1	47036.38		
399.2273	1	1502405.13	C23 H30 N2 O4	(M+H)+
400.2309	1	344794.5	C23 H30 N2 O4	(M+H)+
401.2339	1	52454.1	C23 H30 N2 O4	(M+H)+

Formula Calculator Element Limits

Element	Min	Max
C	3	60
H	0	120
O	0	30
N	0	5

Formula Calculator Results

Formula	CalculatedMass	CalculatedMz	Mz	Diff. (mDa)	Diff. (ppm)	DBE
C23 H30 N2 O4	398.2206	399.2278	399.2273	0.50	1.25	10.0000

--- End Of Report ---

Figure S8 IR spectrum of uncarialine A (**1**)

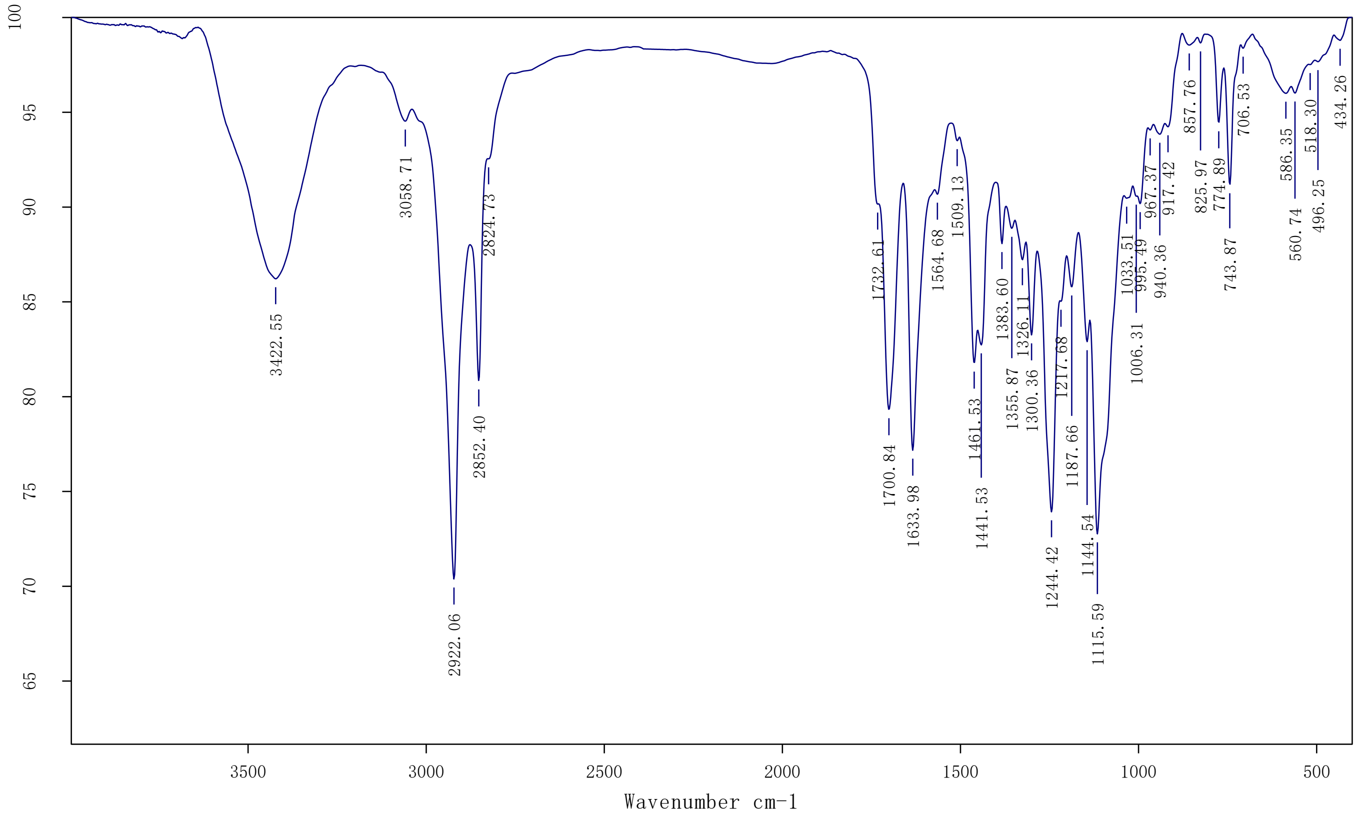

Sample Name: HKP 22b
Sample Form: KBr
Path of File: E:\data
Date of Measurement: 2022/5/31

Resolution: 4
Aperture Setting: 6 mm
Number of Background Scans: 16
Number of Sample Scans: 16

Beamsplitter Setting: KBr
Source Setting: MIR
Instrument Type: BRUKER VERTEX 70
Soft Version: OPUS8.1

Figure S9 ECD spectrum of uncarialine A (**1**)

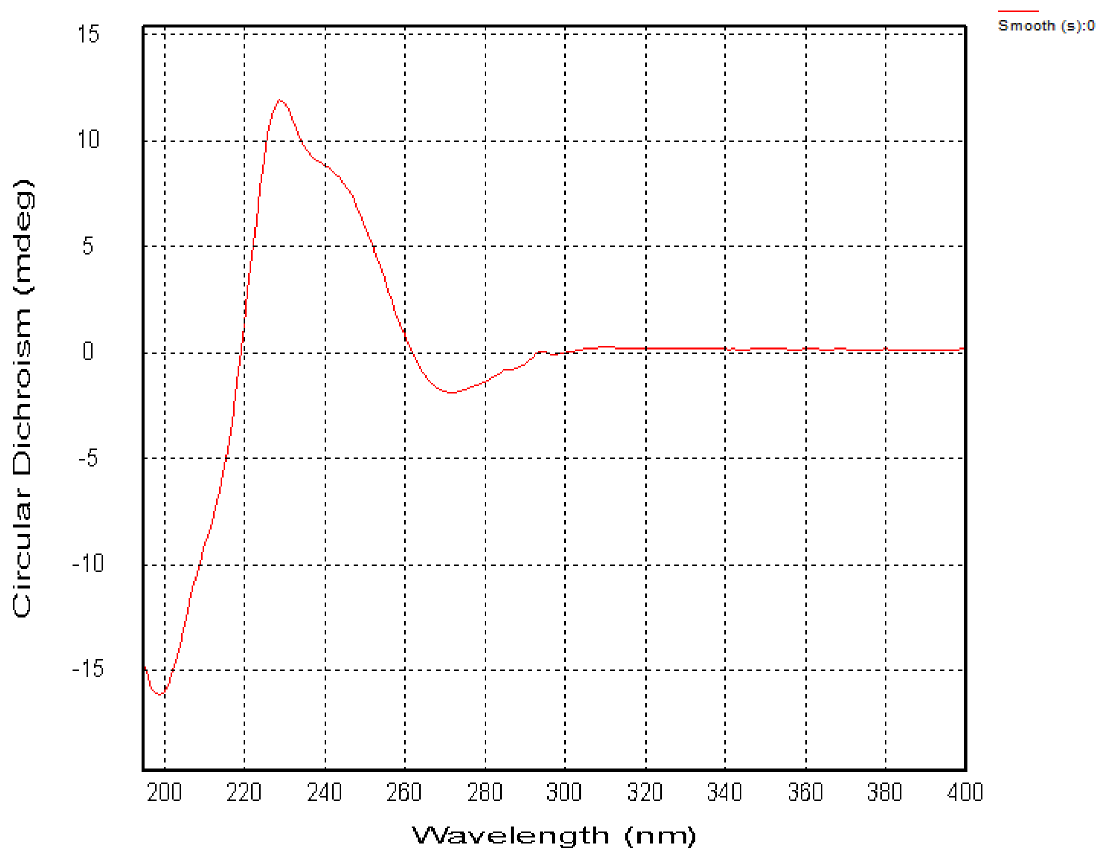

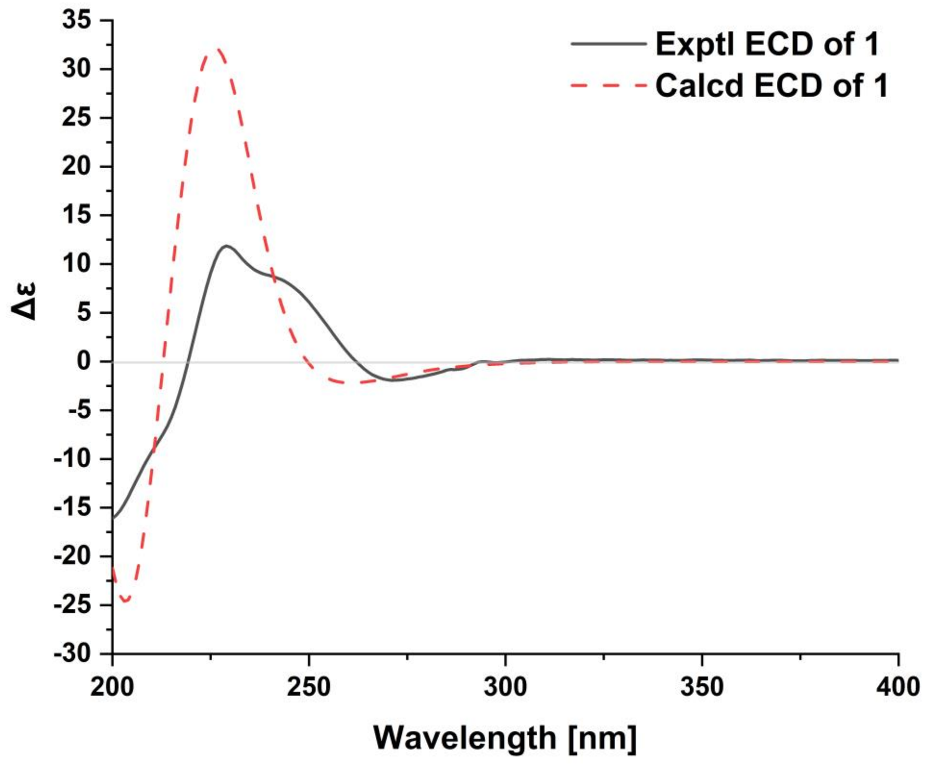

Figure S10 ^{1}H NMR spectrum of uncarialine B (**2**) in $CDCl_3$

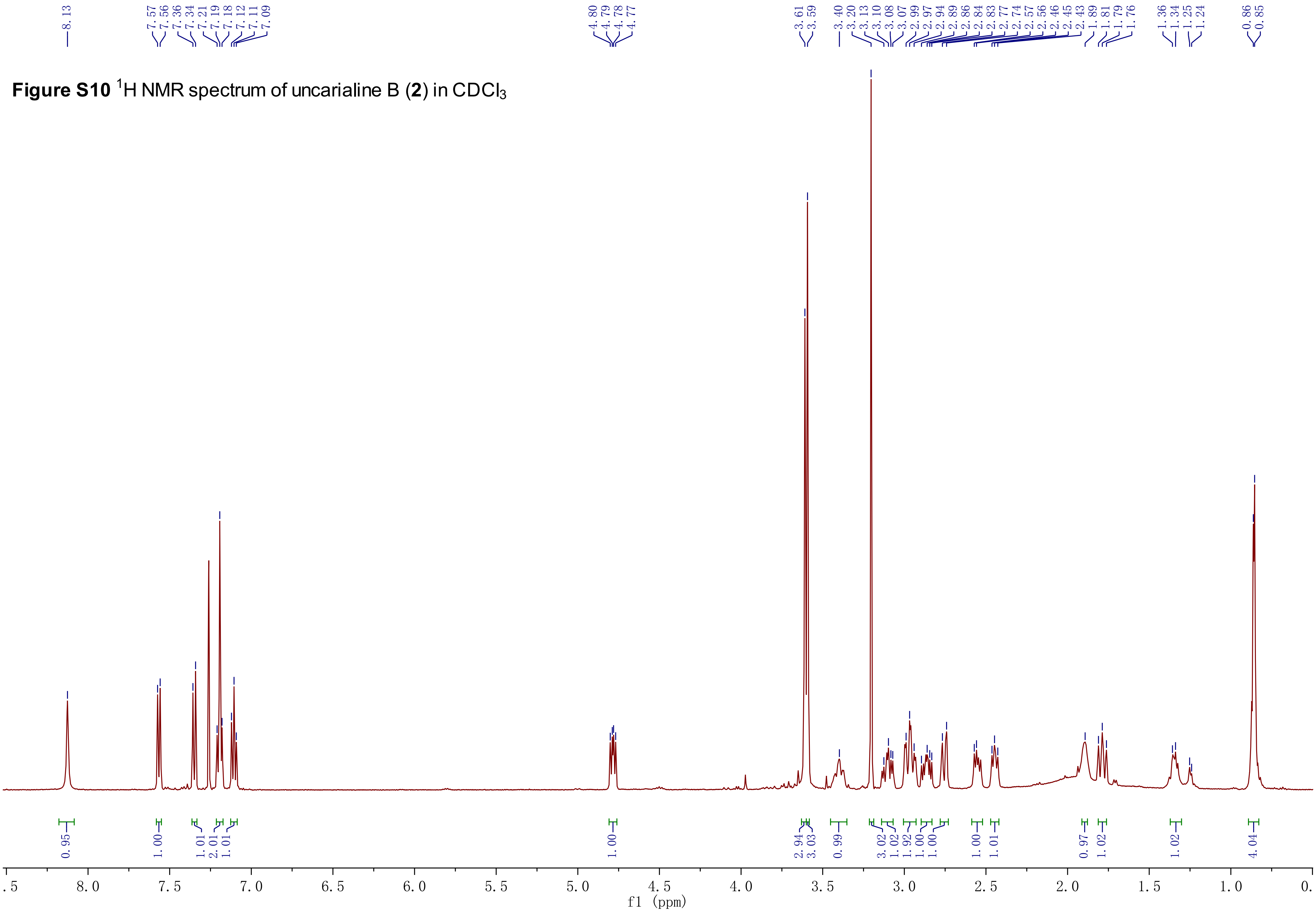

Figure S11 ^{13}C NMR spectrum of uncarialine B (**2**) in $CDCl_3$

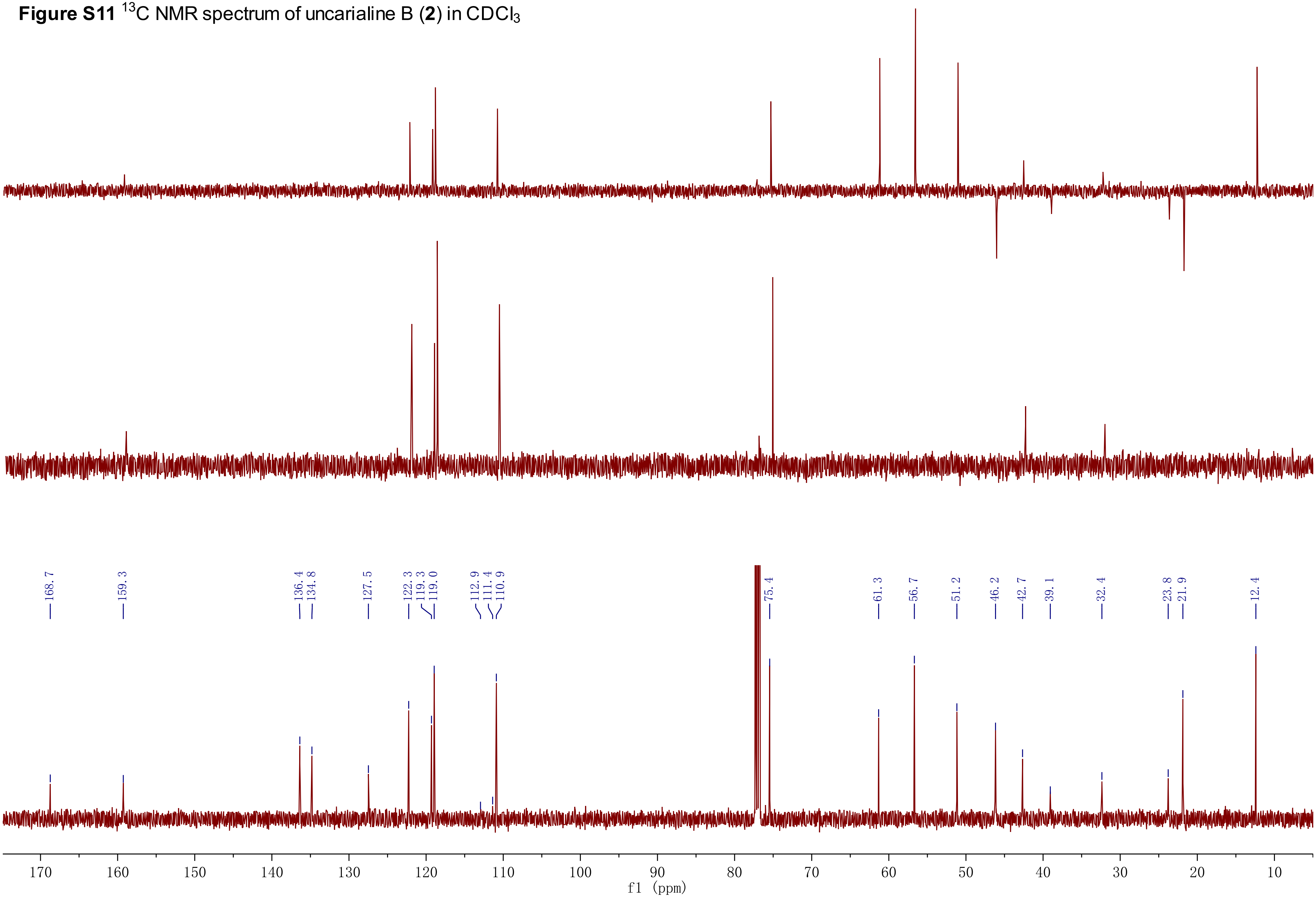

Figure S12 HSQC spectrum of uncarialine B (**2**) in $CDCl_3$

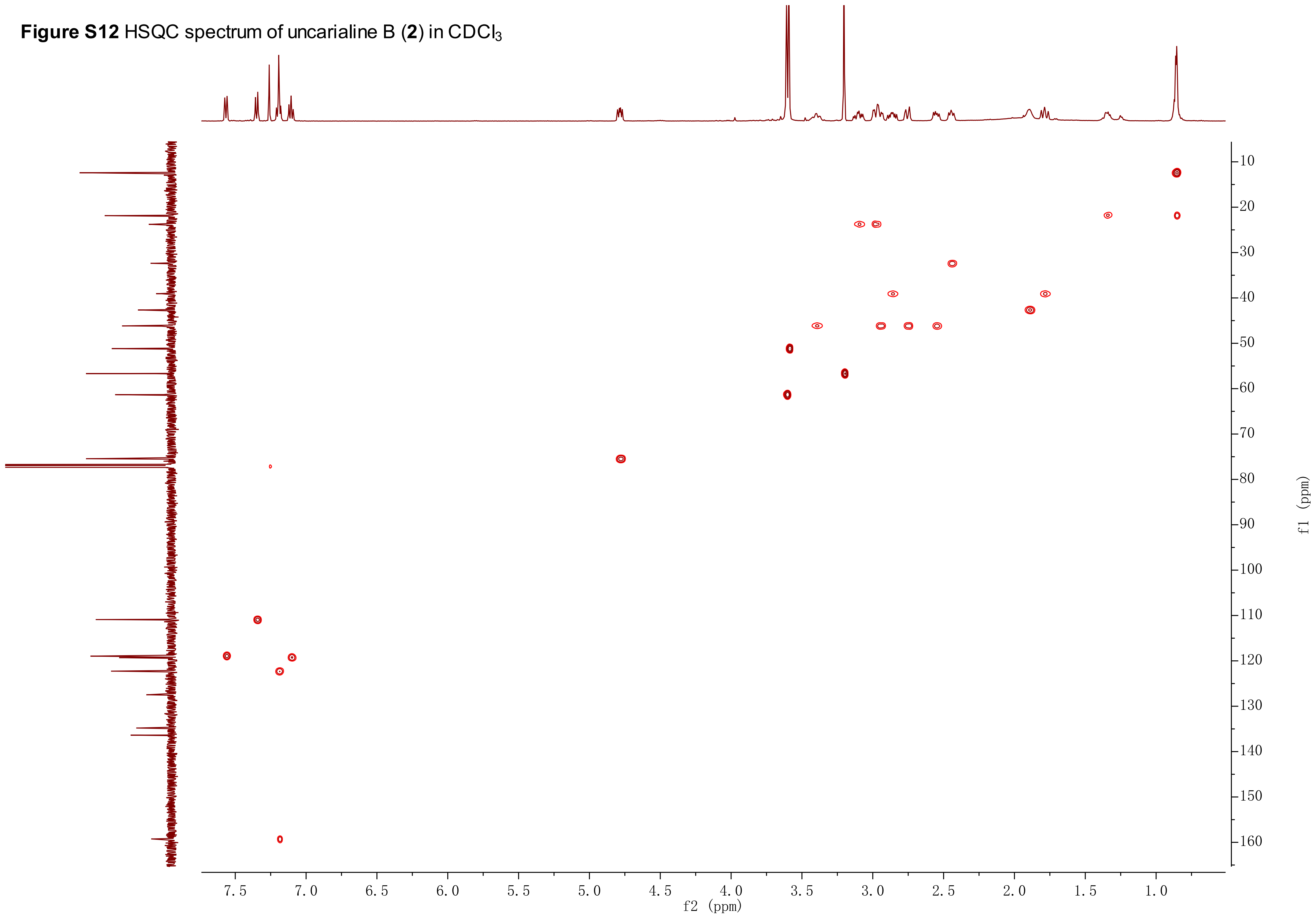

Figure S13 ^{1}H-^{1}H COSY spectrum of uncarialine B (**2**) in $CDCl_3$

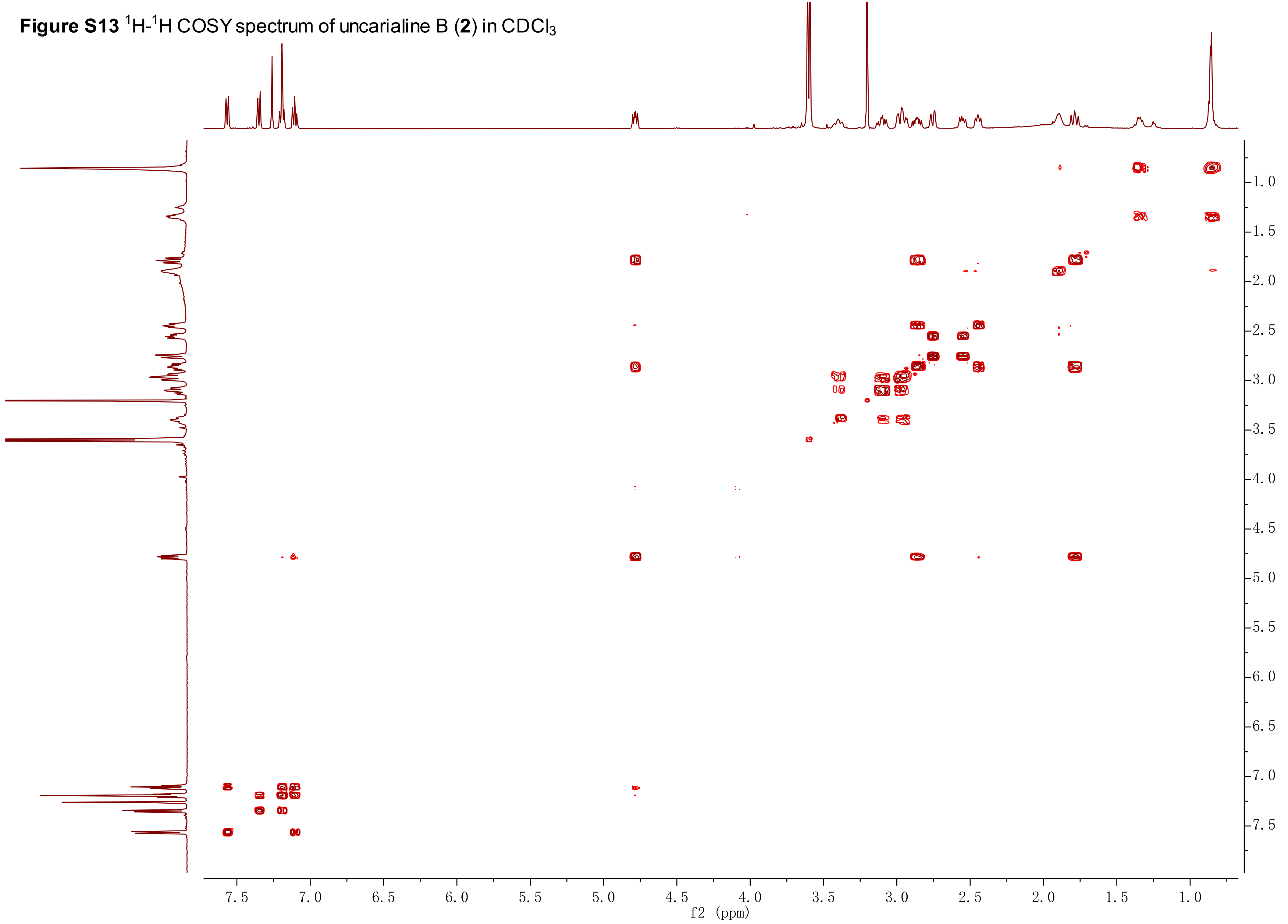

Figure S14 HMBC spectrum of uncarialine B (**2**) in $CDCl_3$

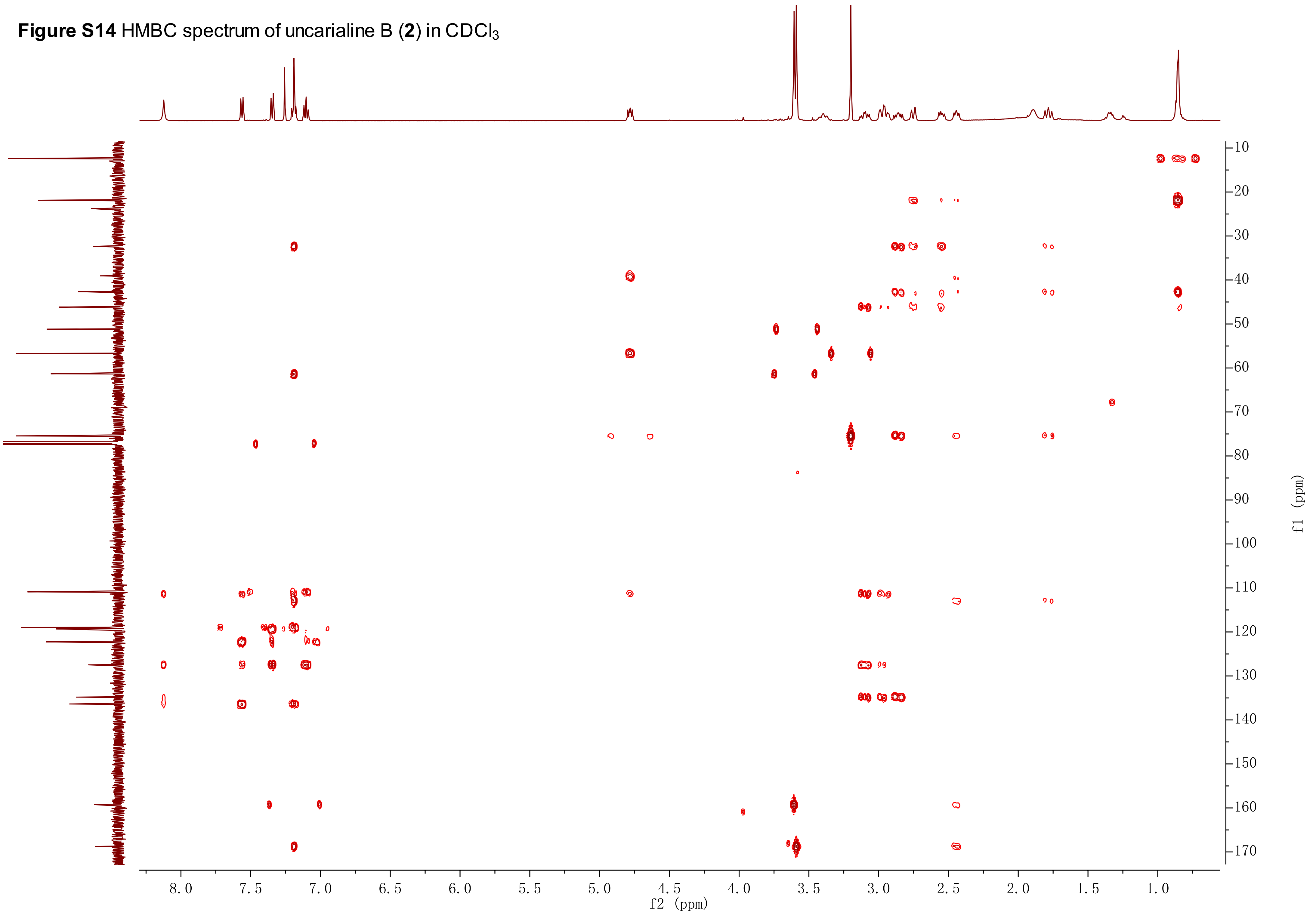

Figure S15 ROESY spectrum of uncarialine B (**2**) in $CDCl_3$

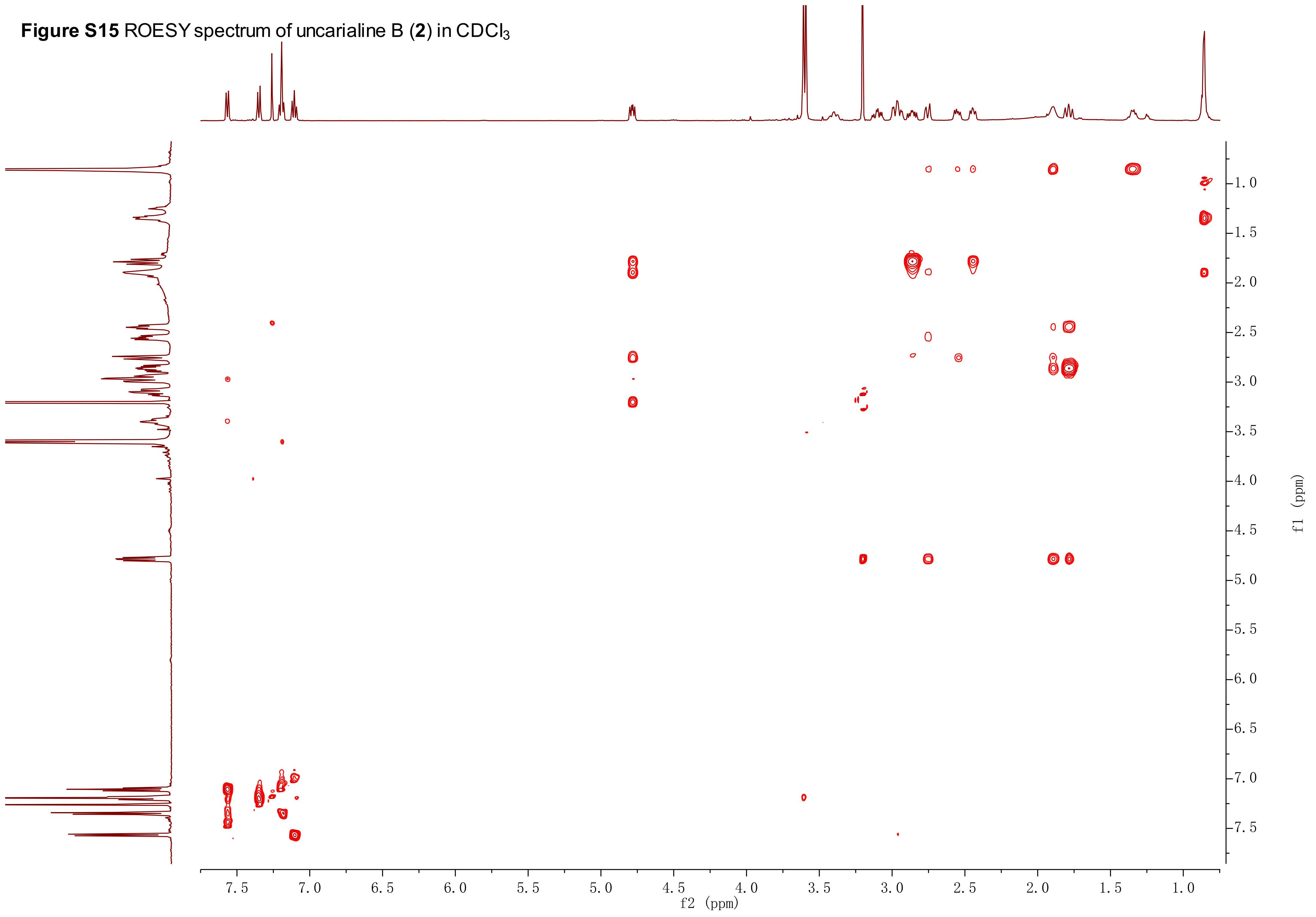

Figure S16 HRESIMS spectrum of uncarialine B (**2**)

Qualitative Analysis Report

**Data Filename**	HKP-24b.d	**Sample Name**	HKP-24b
**Sample Type**	Sample	**Position**	P1-D1
**Instrument Name**	Instrument 1	**User Name**	
**Acq Method**	s.m	**Acquired Time**	4/24/2022 10:19:06 AM
**IRM Calibration Status**	Success	**DA Method**	PCDL.m
**Comment**			

Sample Group **Info.**

Acquisition SW Version 6200 series TOF/6500 series Q-TOF B.05.01 (B5125.2)

User Spectra

Fragmentor Voltage	Collision Energy	Ionization Mode
135	0	ESI

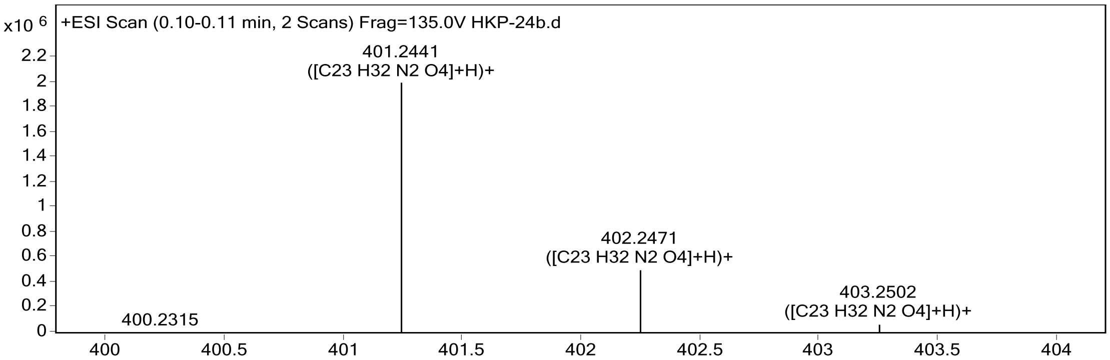

Peak List

m/z	z	Abund	Formula	Ion
102.1281	1	702155.13		
103.1314	1	94227.34		
317.241	1	21873.19		
369.2175	1	70690.55		
401.2441	1	1996076.88	C23 H32 N2 O4	(M+H)+
402.2471	1	504384.06	C23 H32 N2 O4	(M+H)+
403.2502	1	72827.05	C23 H32 N2 O4	(M+H)+
411.2282	1	73058.95		
801.4797	1	44975.65		
802.4827	1	24185.73		

Formula Calculator Element Limits

Element	Min	Max
C	3	60
H	0	120
O	0	30
N	0	5

Formula Calculator Results

Formula	CalculatedMass	CalculatedMz	Mz	Diff. (mDa)	Diff. (ppm)	DBE
C23 H32 N2 O4	400.2362	401.2435	401.2441	-0.60	-1.50	9.0000

--- End Of Report ---

Figure S17 IR spectrum of uncarialine B (**2**)

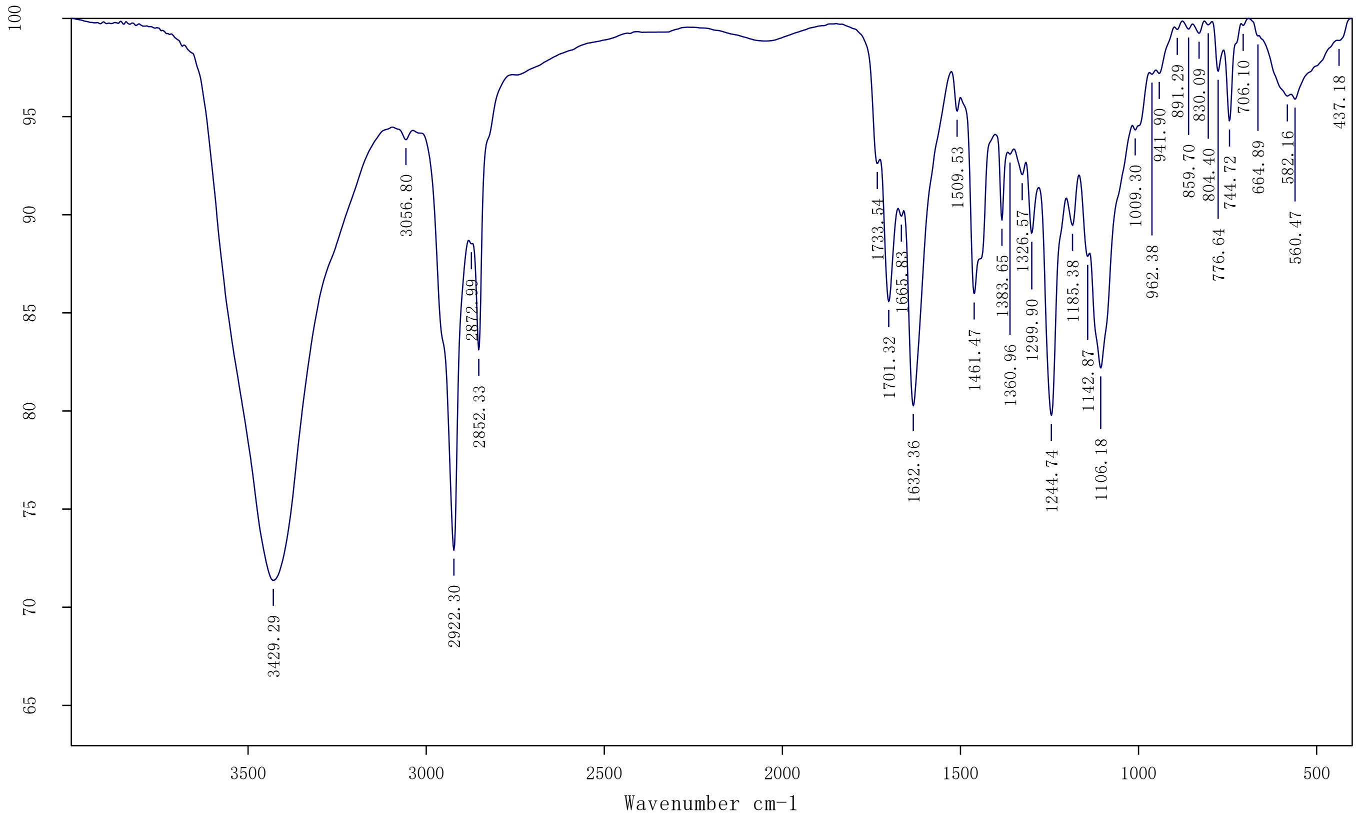

Sample Name: HKP 24b
Sample Form: KBr
Path of File: E:\data
Date of Measurement: 2022/5/31

Resolution: 4
Aperture Setting: 6 mm
Number of Background Scans: 16
Number of Sample Scans: 16

Beamsplitter Setting: KBr
Source Setting: MIR
Instrument Type: BRUKER VERTEX 70
Soft Version: OPUS8.1

Figure S18 ECD spectrum of uncarialine B (**2**)

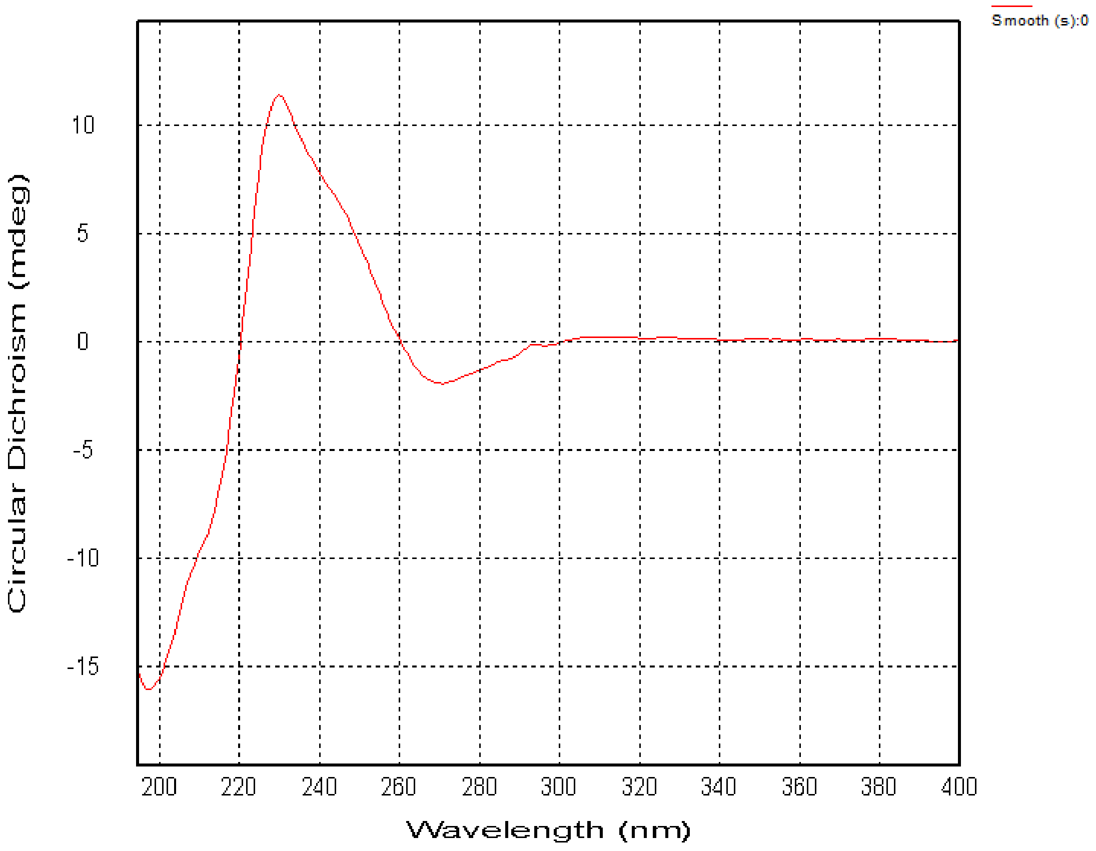

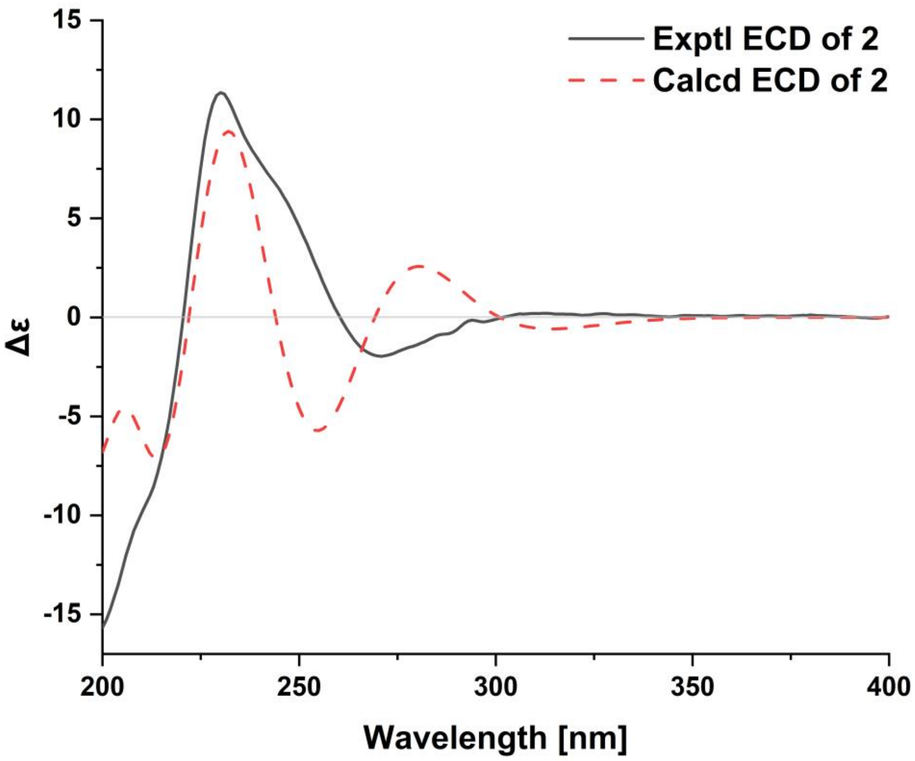

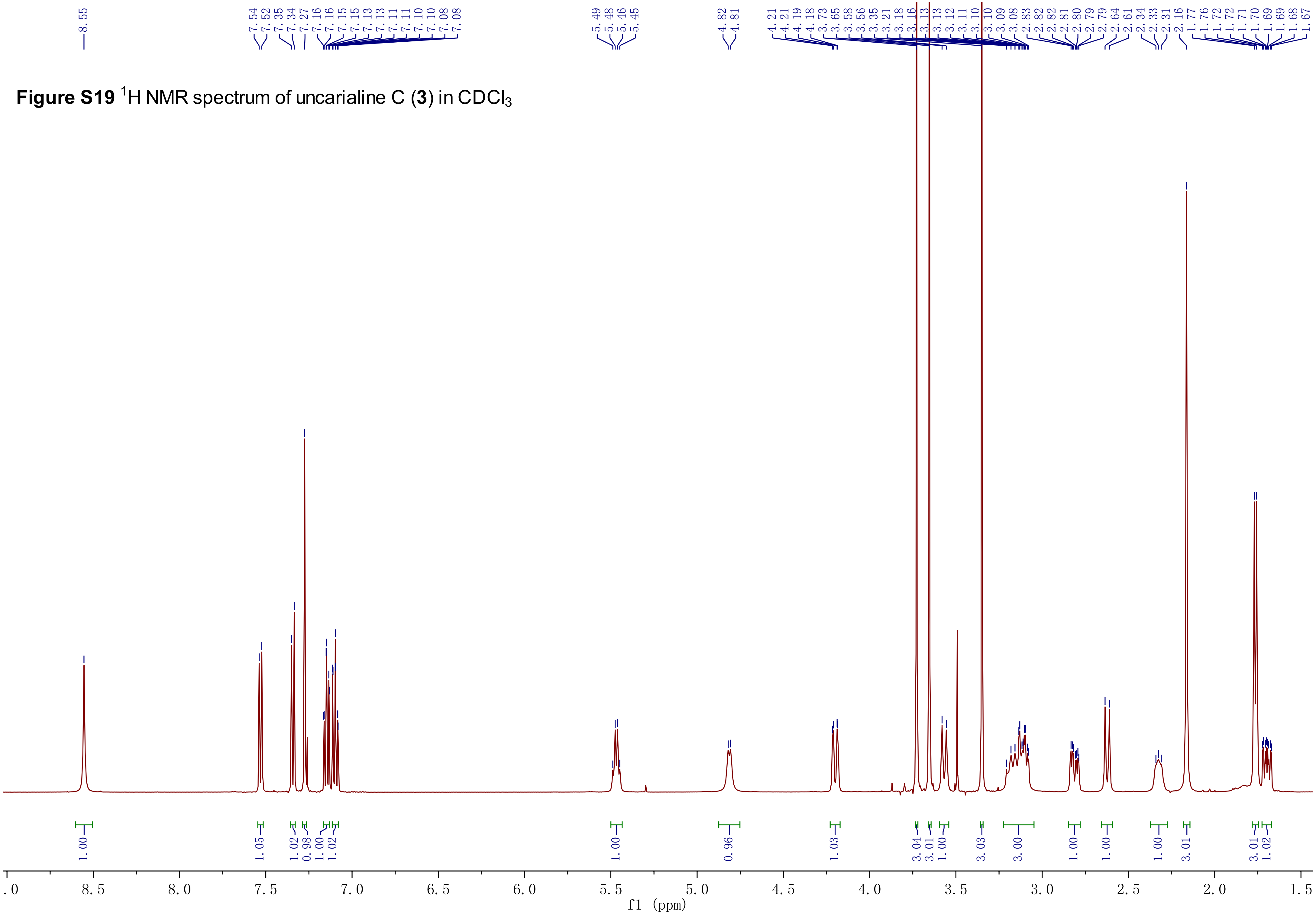

Figure S19 ^1H NMR spectrum of uncarialine C (**3**) in $CDCl_3$

Figure S20 ^{13}C NMR spectrum of uncarialine C (**3**) in $CDCl_3$

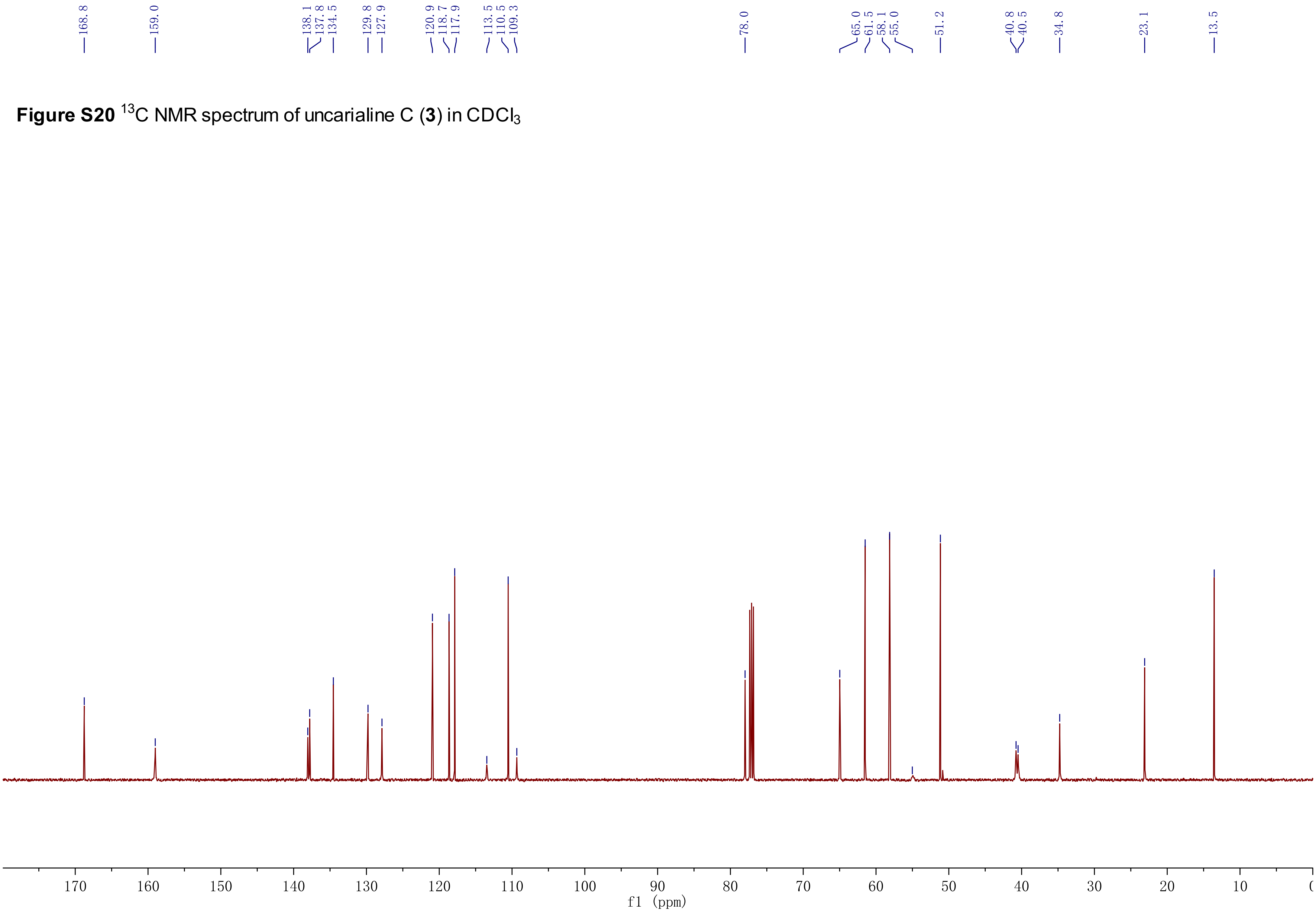

Figure S21 HSQC spectrum of uncarialine C (**3**) in $CDCl_3$

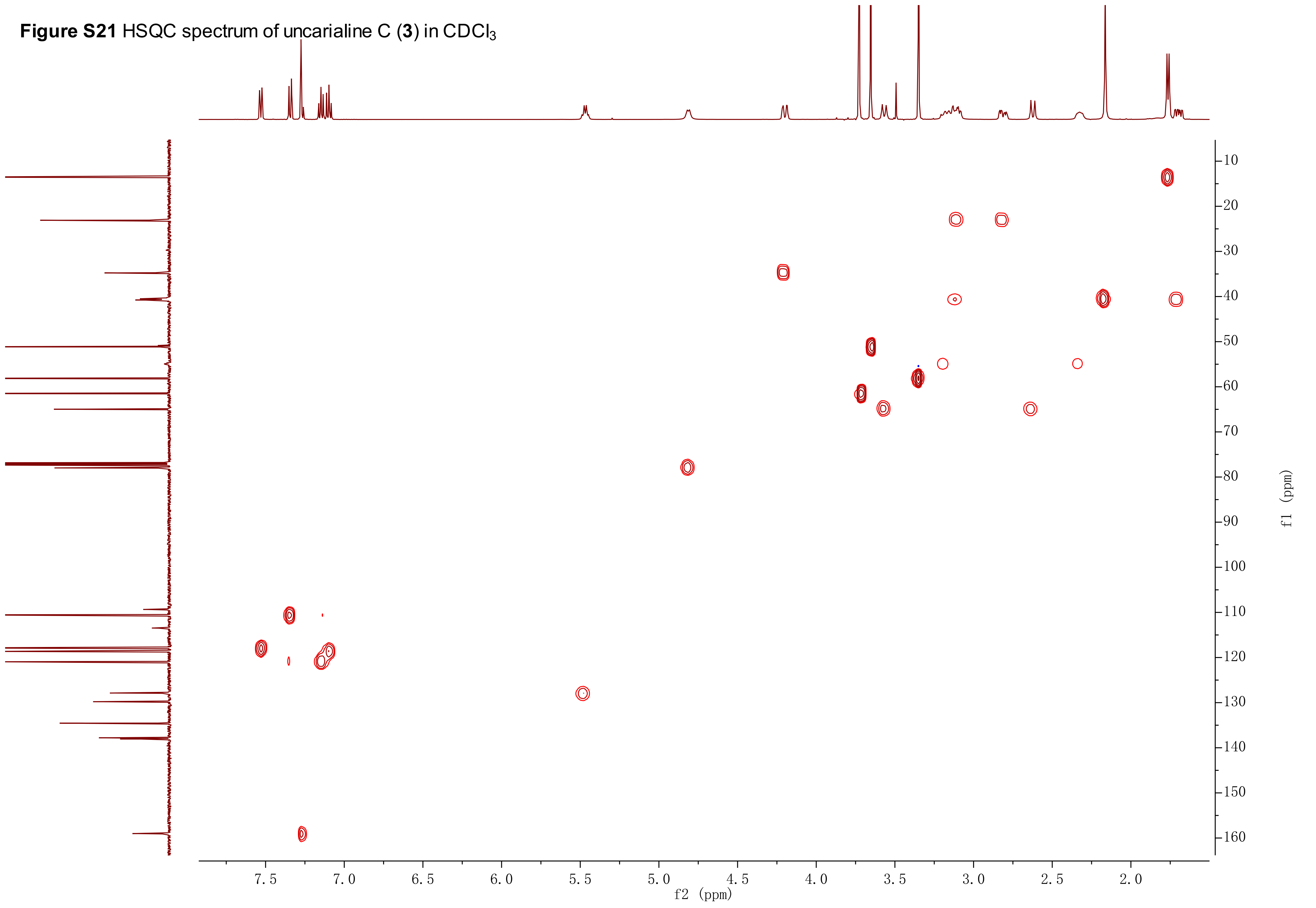

Figure S22 ^{1}H-^{1}H COSY spectrum of uncarialine C (**3**) in $CDCl_3$

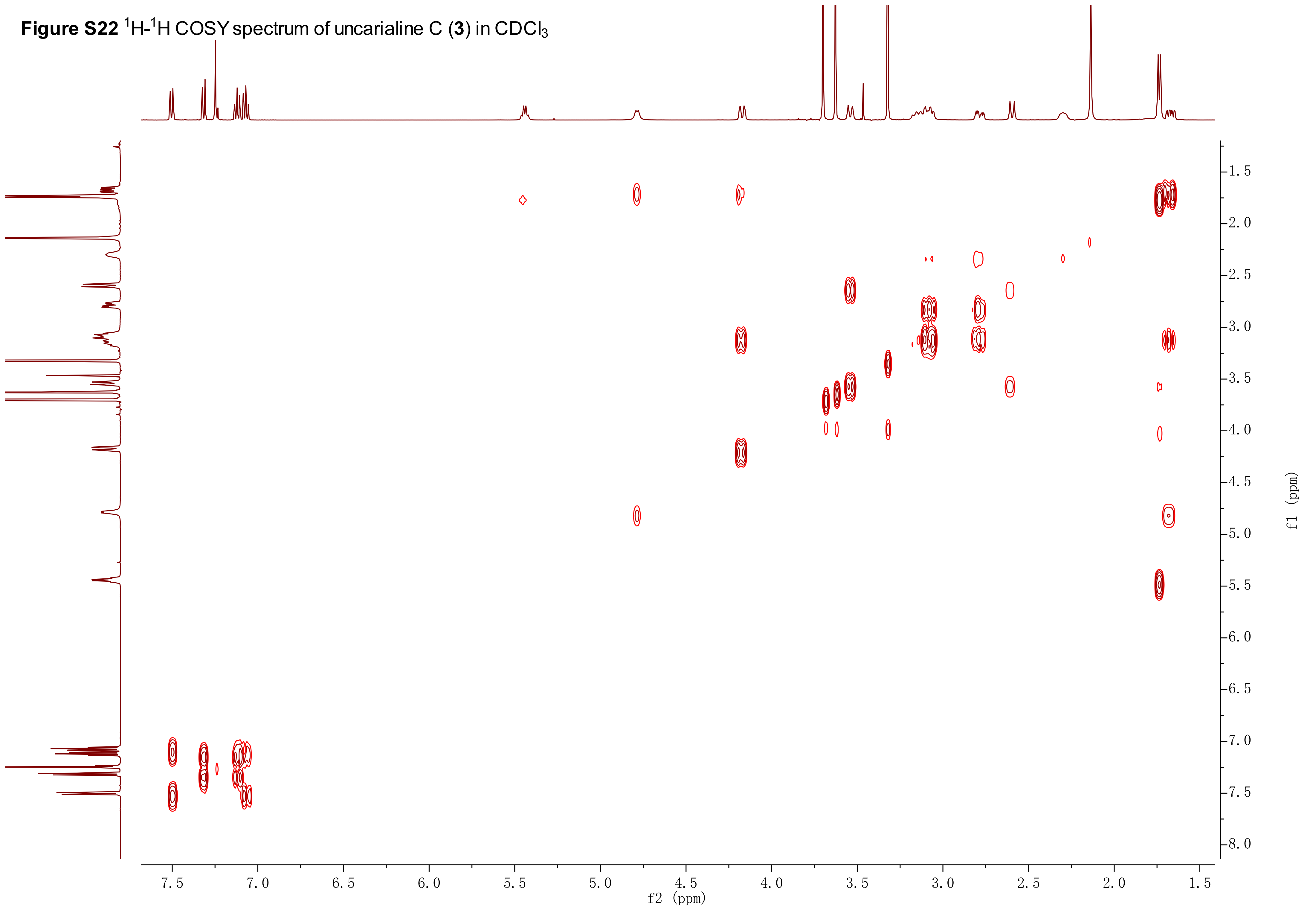

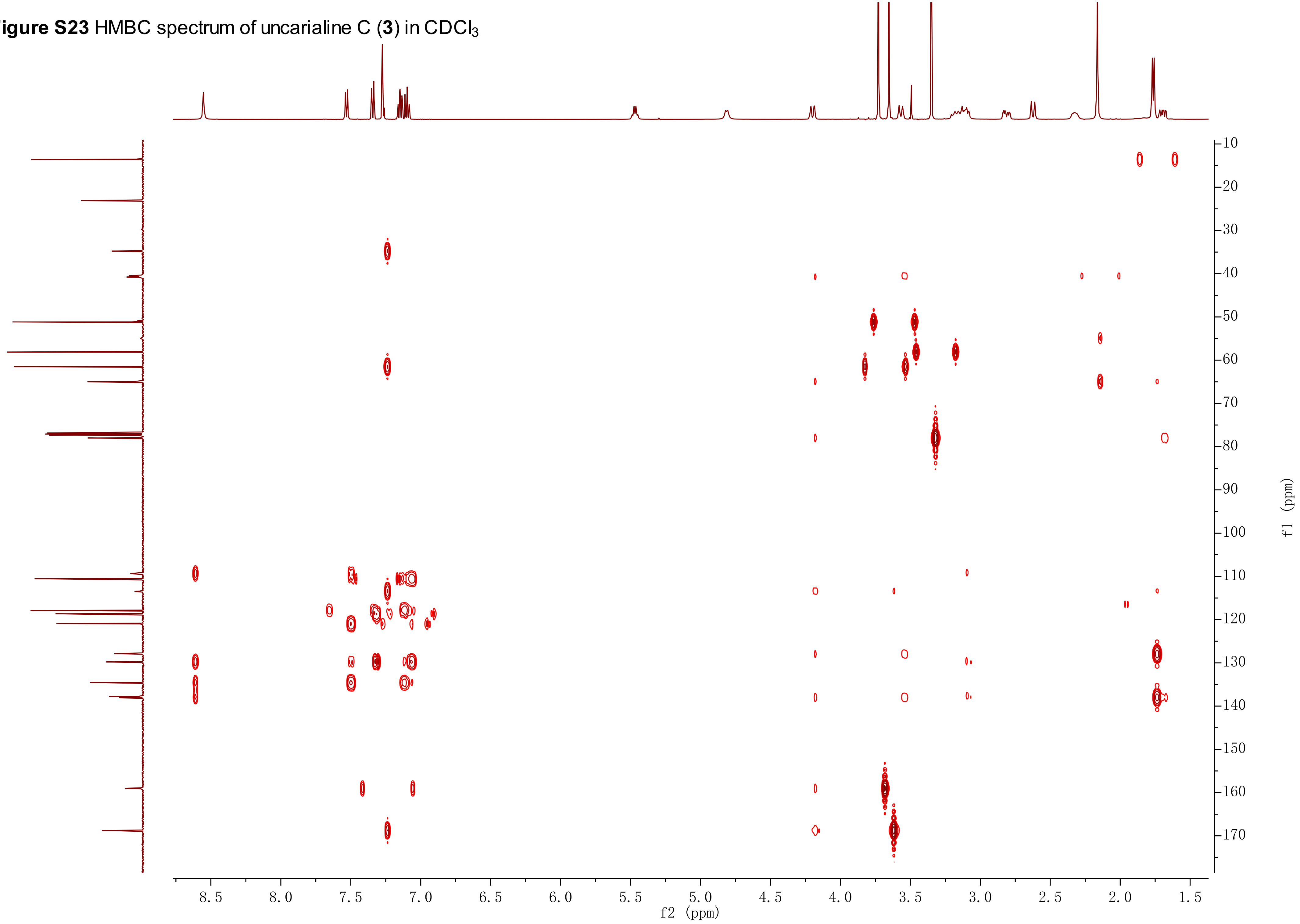

Figure S23 HMBC spectrum of uncarialine C (**3**) in $CDCl_3$

Figure S24 ROESY spectrum of uncarialine C (**3**) in $CDCl_3$

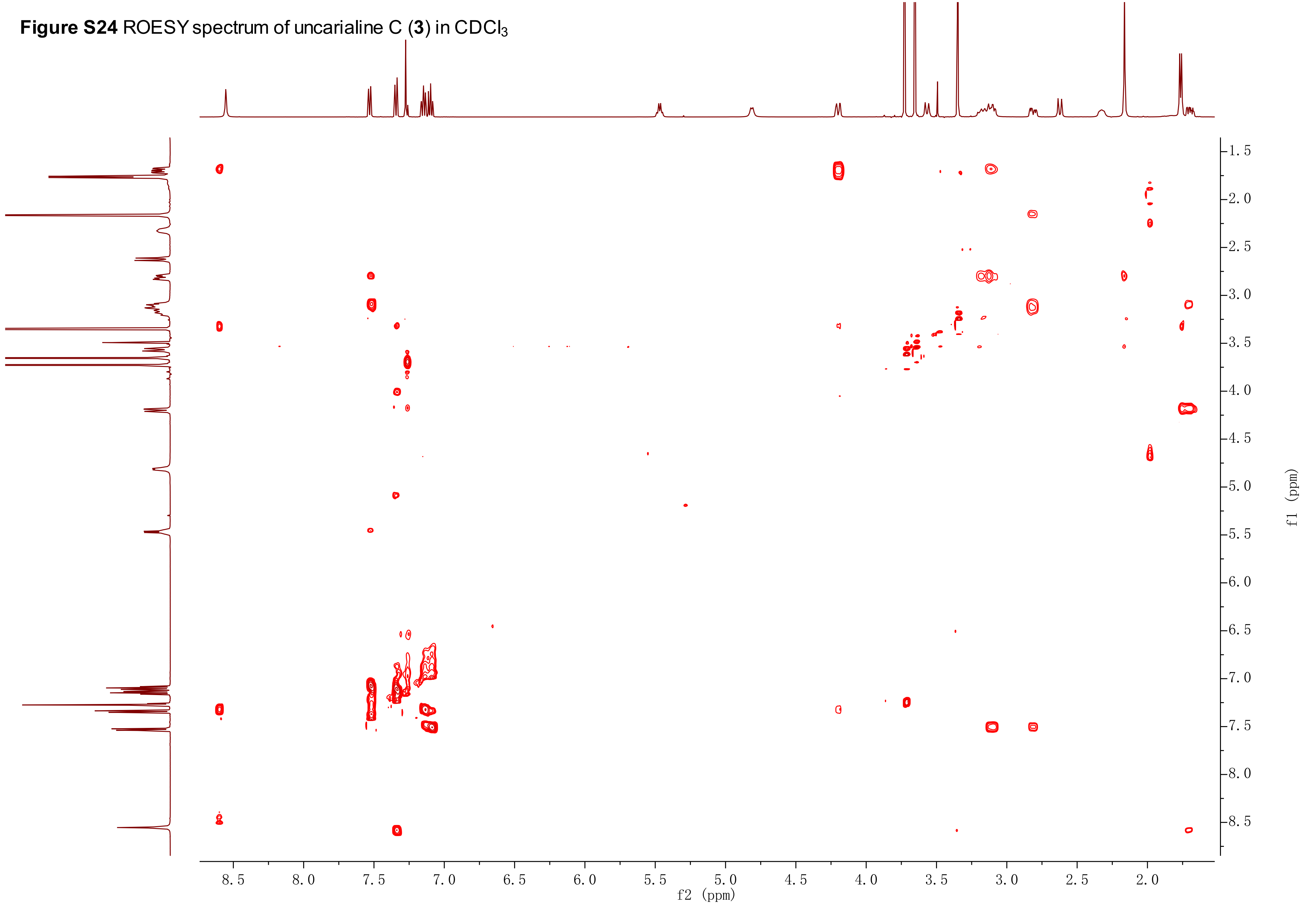

Figure S25 HRESIMS spectrum of uncarialine C (**3**)

Qualitative Analysis Report

**Data Filename**	HKP-40b.d	**Sample Name**	HKP-40b
**Sample Type**	Sample	**Position**	P1-C8
**Instrument Name**	Instrument 1	**User Name**	
**Acq Method**	s.m	**Acquired Time**	12/26/2022 3:16:02 PM
**IRM Calibration Status**	Success	**DA Method**	PCDL.m
**Comment**			

Sample Group **Info.**

Acquisition SW 6200 series TOF/6500 series
Version Q-TOF B.05.01 (B5125.2)

User Spectra

Fragmentor Voltage	Collision Energy	Ionization Mode
135	0	ESI

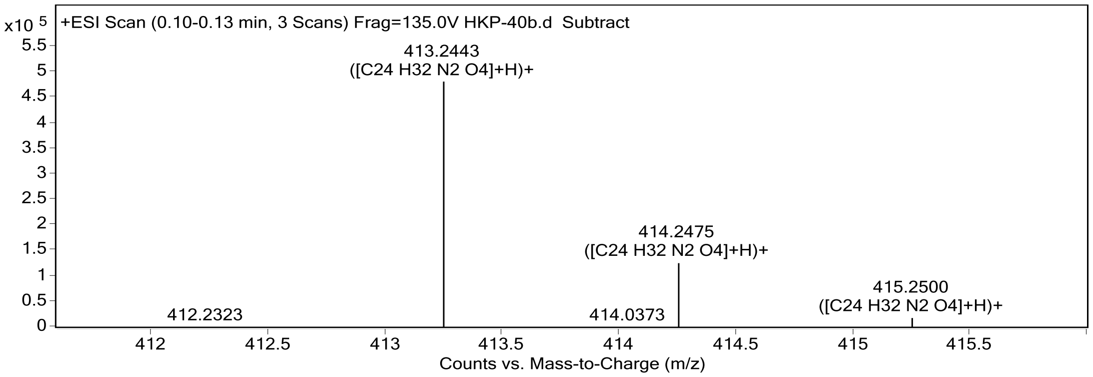

Peak List

m/z	z	Abund	Formula	Ion
381.2176	1	980.02		
413.2443	1	480915.72	C24 H32 N2 O4	(M+H)+
414.2475	1	126737.84	C24 H32 N2 O4	(M+H)+
415.25	1	18345.72	C24 H32 N2 O4	(M+H)+
416.2525	1	2360.06	C24 H32 N2 O4	(M+H)+
427.2237	1	856.42		
429.2381	1	3125.48		
430.2434	1	839.35		
435.2252	1	1039.14		
922.0098	1	2157.16		

Formula Calculator Element Limits

Element	Min	Max
C	3	60
H	0	150
O	0	10
N	0	8
S	0	1

Formula Calculator Results

Formula	CalculatedMass	CalculatedMz	Mz	Diff. (mDa)	Diff. (ppm)	DBE
C24 H32 N2 O4	412.2362	413.2435	413.2443	-0.80	-1.94	10.0000

--- End Of Report ---

Figure S26 IR spectrum of uncarialine C (**3**)

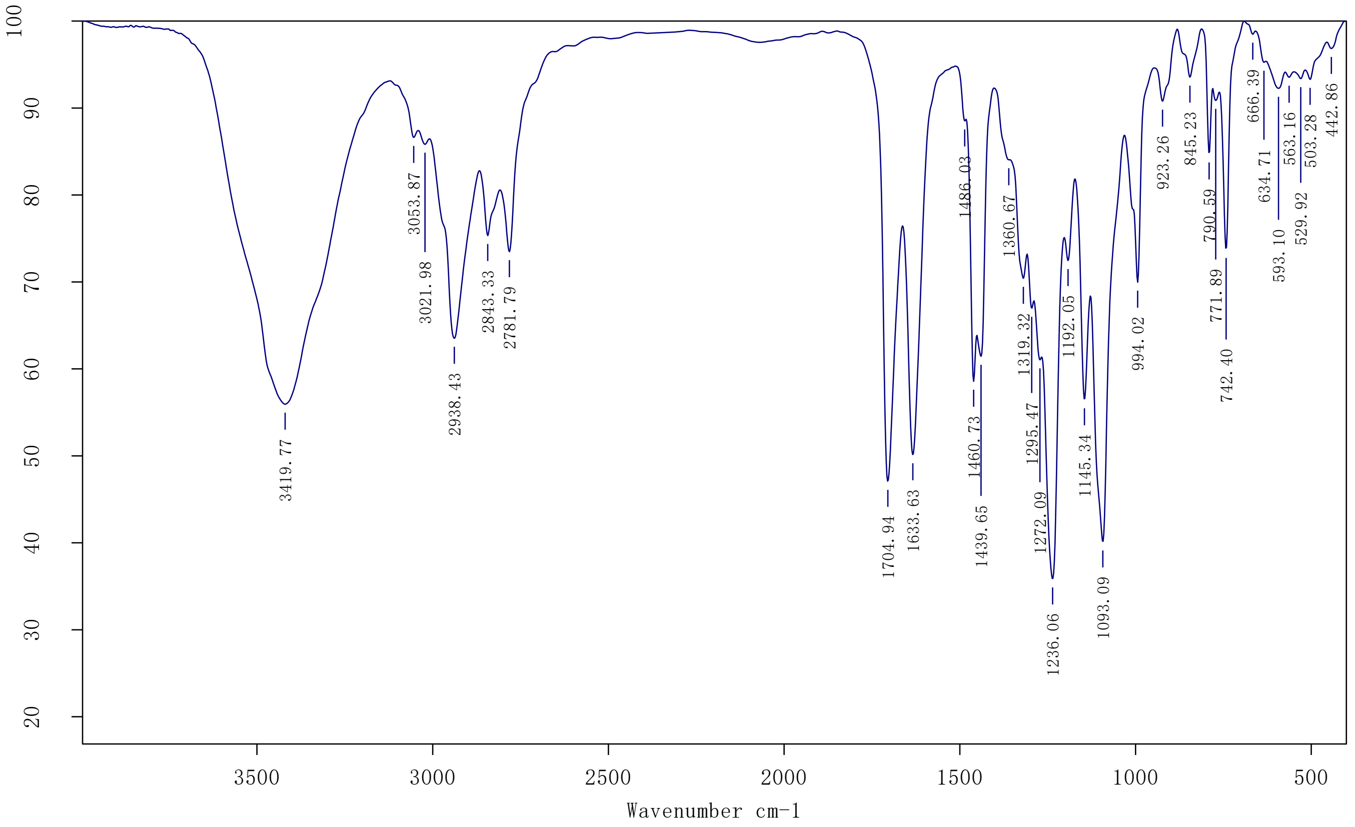

Sample Name: HKP-40b
Sample Form: KBr
Path of File: E:\data
Date of Measurement: 2023/1/17

Resolution: 4
Aperture Setting: 6 mm
Number of Background Scans: 16
Number of Sample Scans: 16

Beamsplitter Setting: KBr
Source Setting: MIR
Instrument Type: BRUKER VERTEX 70
Soft Version: OPUS8.1

Figure S27 ECD spectrum of uncarialine C (**3**)

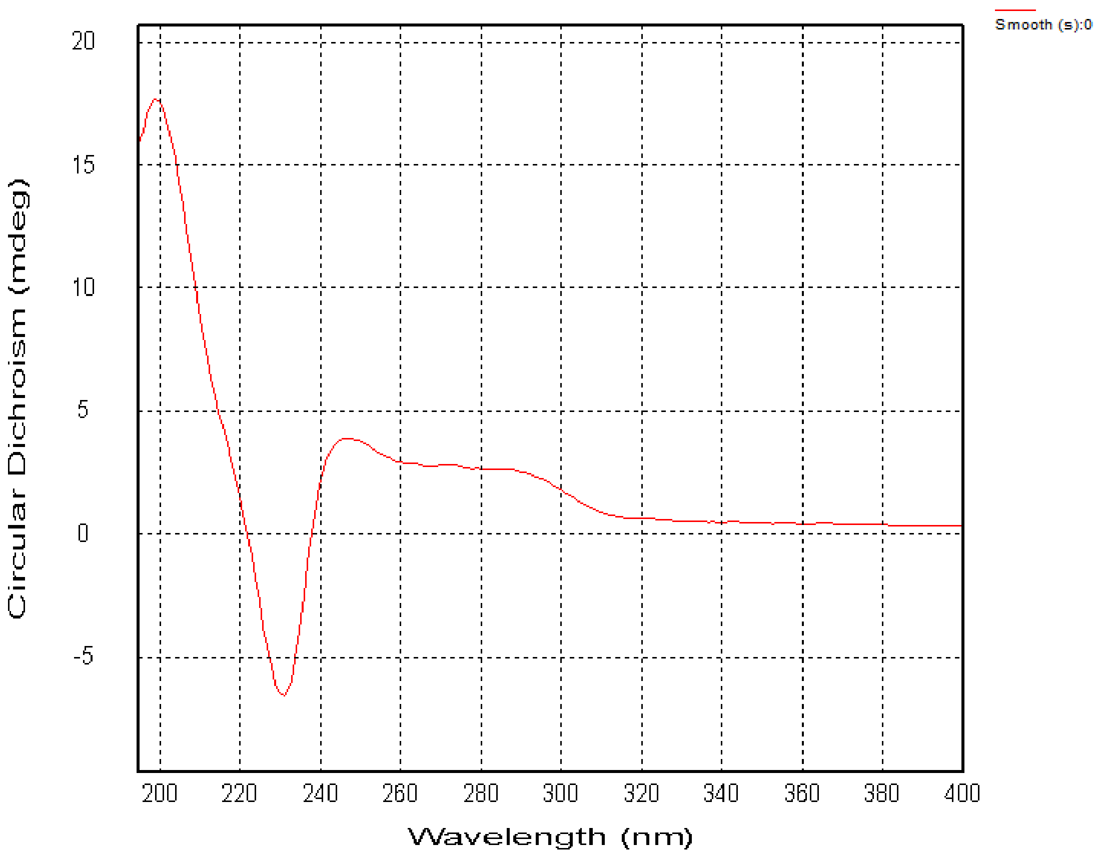

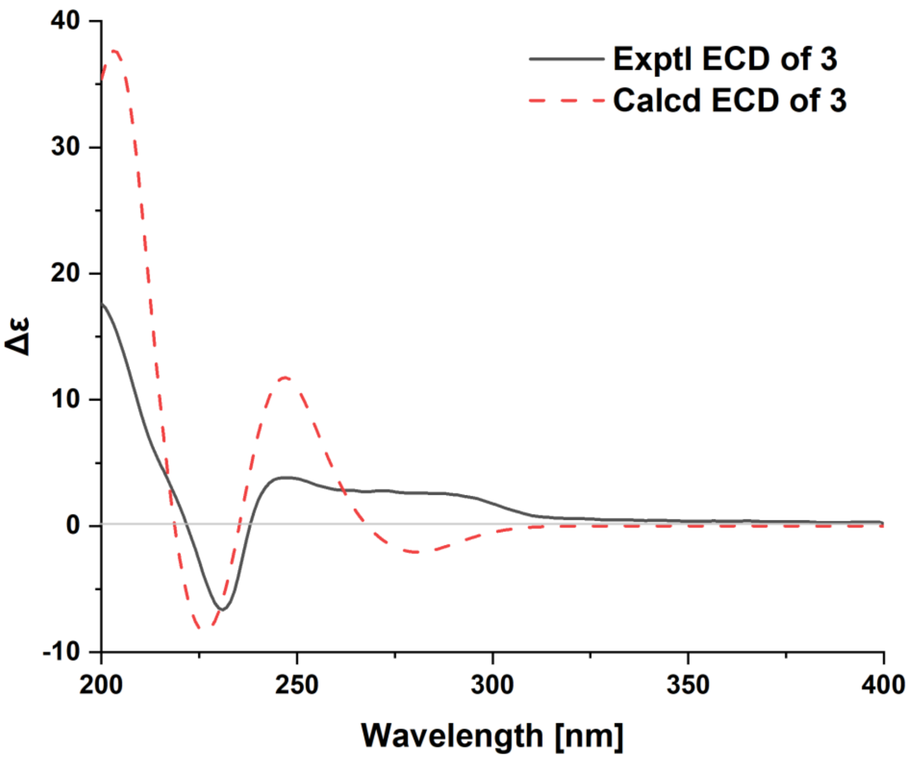

Figure S28 ^{1}H NMR spectrum of uncarialine D (**4**) in CD_3OD

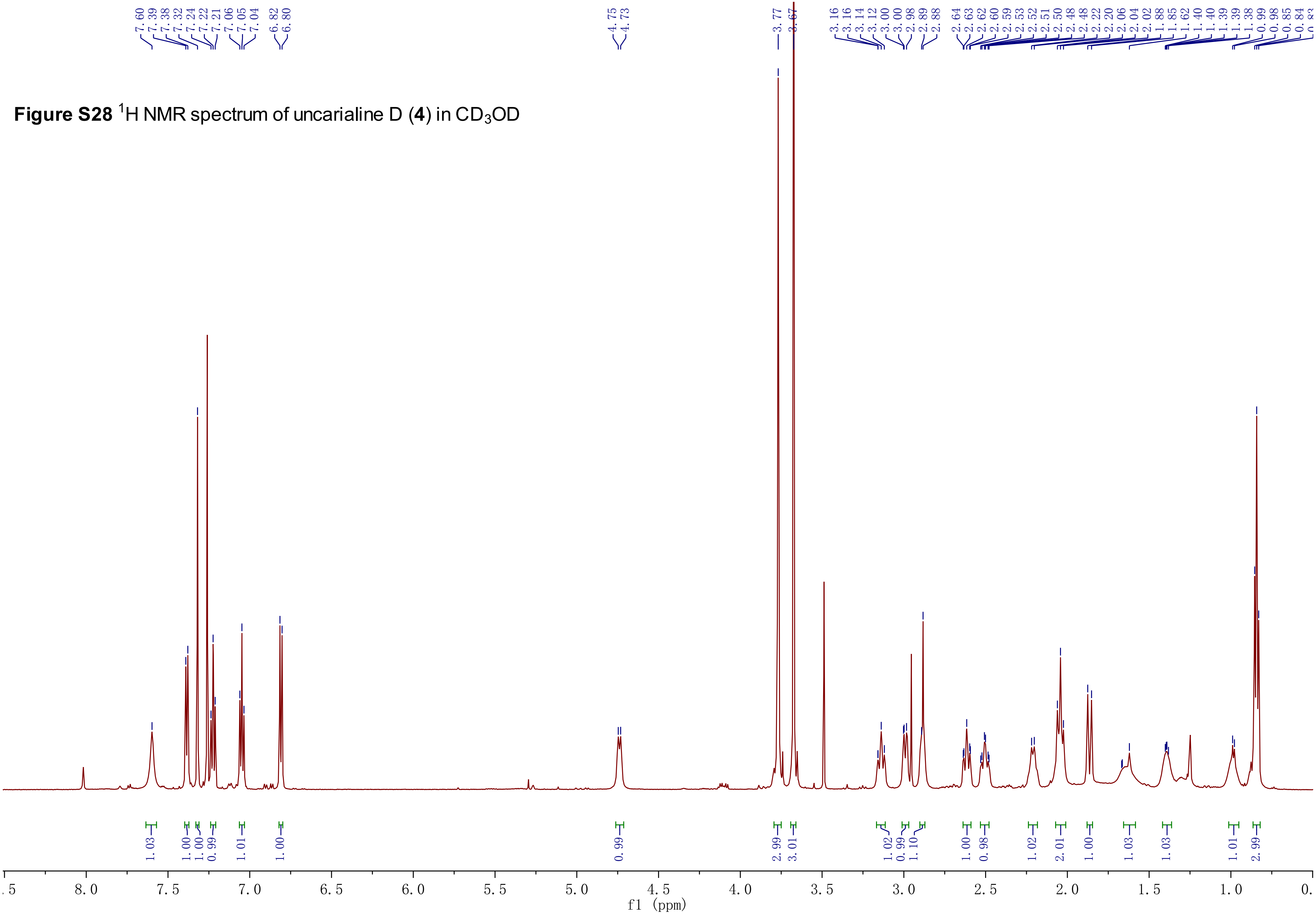

Figure S29 ^{13}C NMR spectrum of uncarialine D (**4**) in CD_3OD

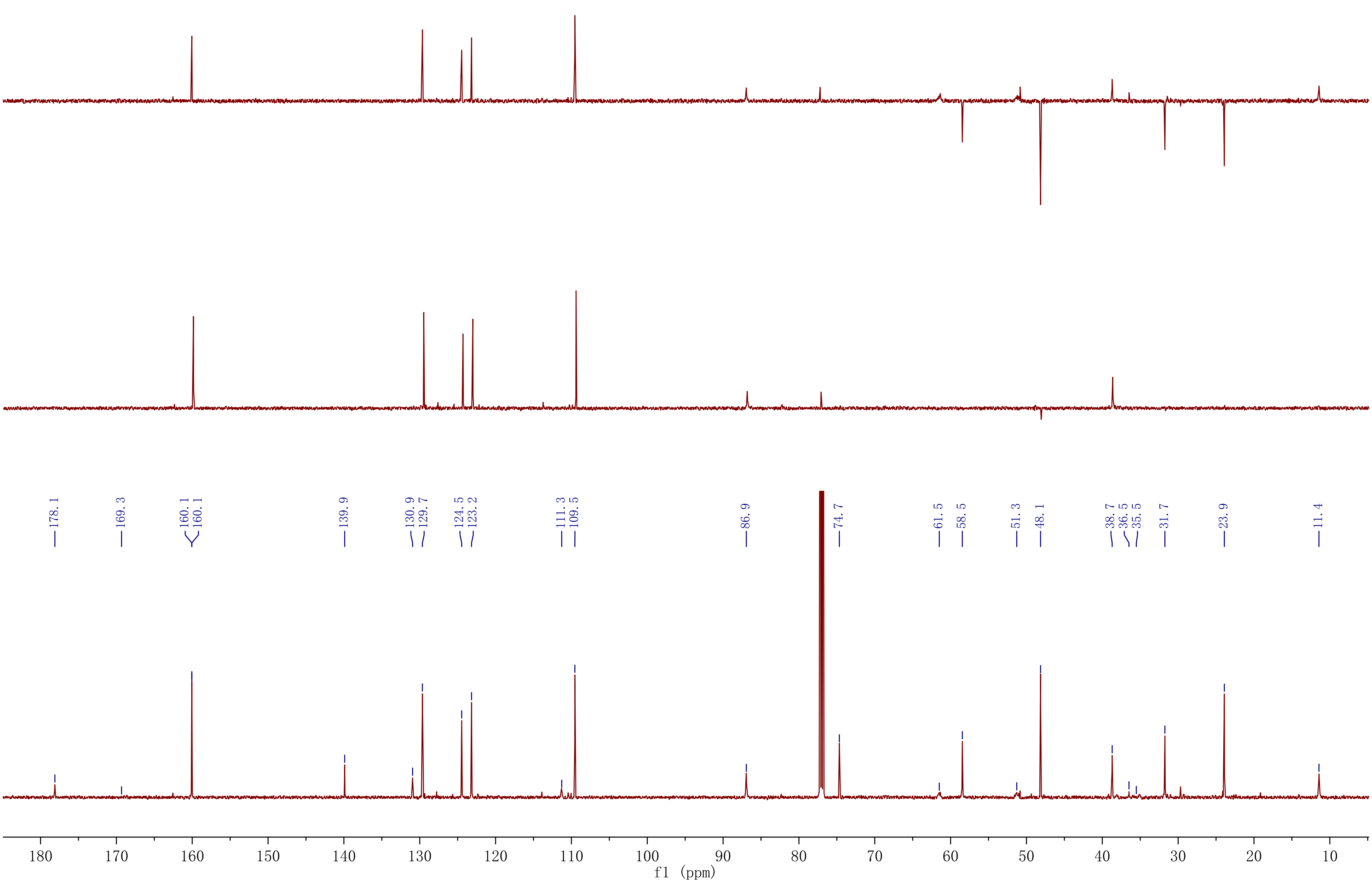

Figure S30 HSQC spectrum of uncarialine D (**4**) in CD_3OD

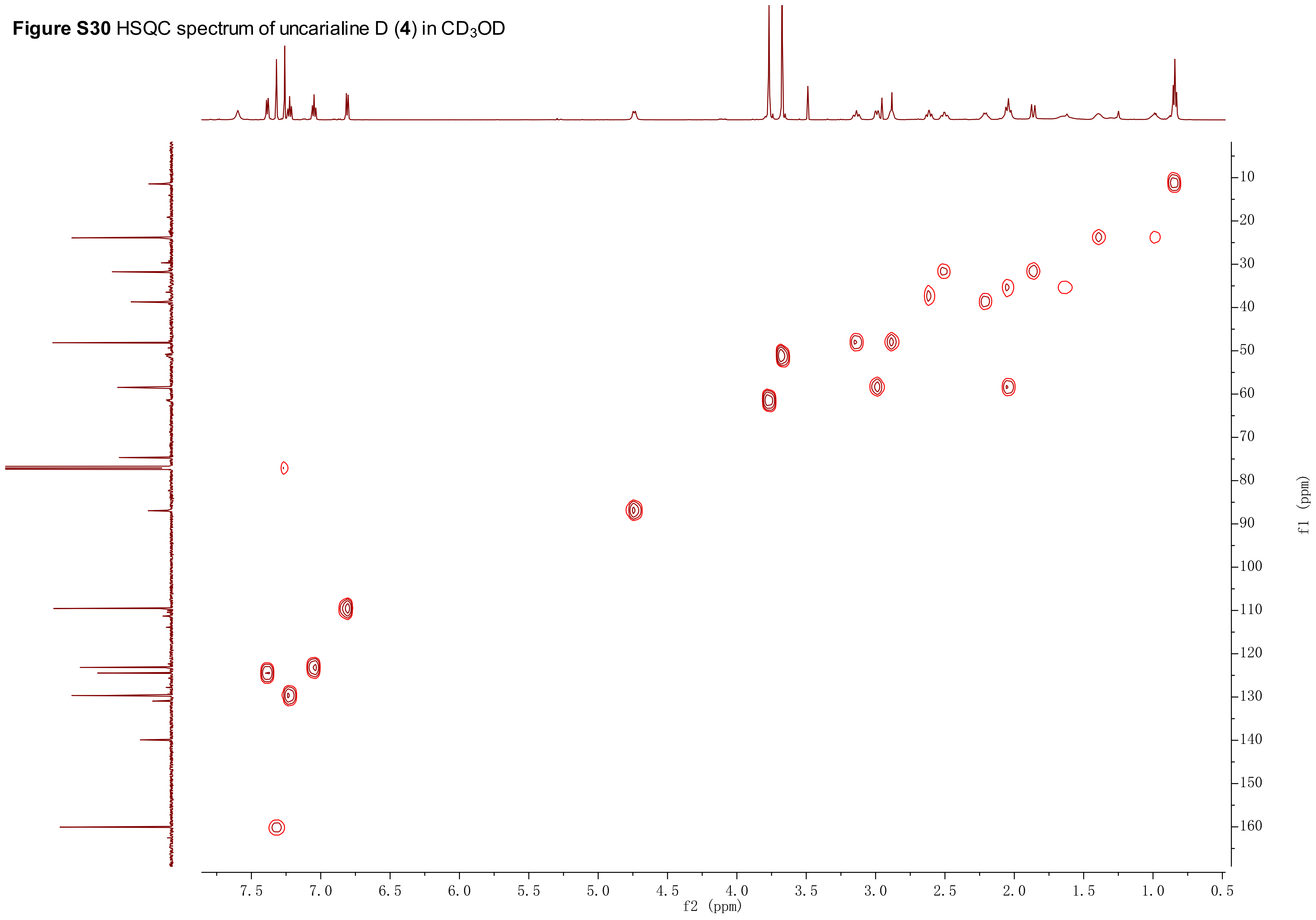

Figure S31 ^1H-^1H COSY spectrum of uncarialine D (**4**) in CD_3OD

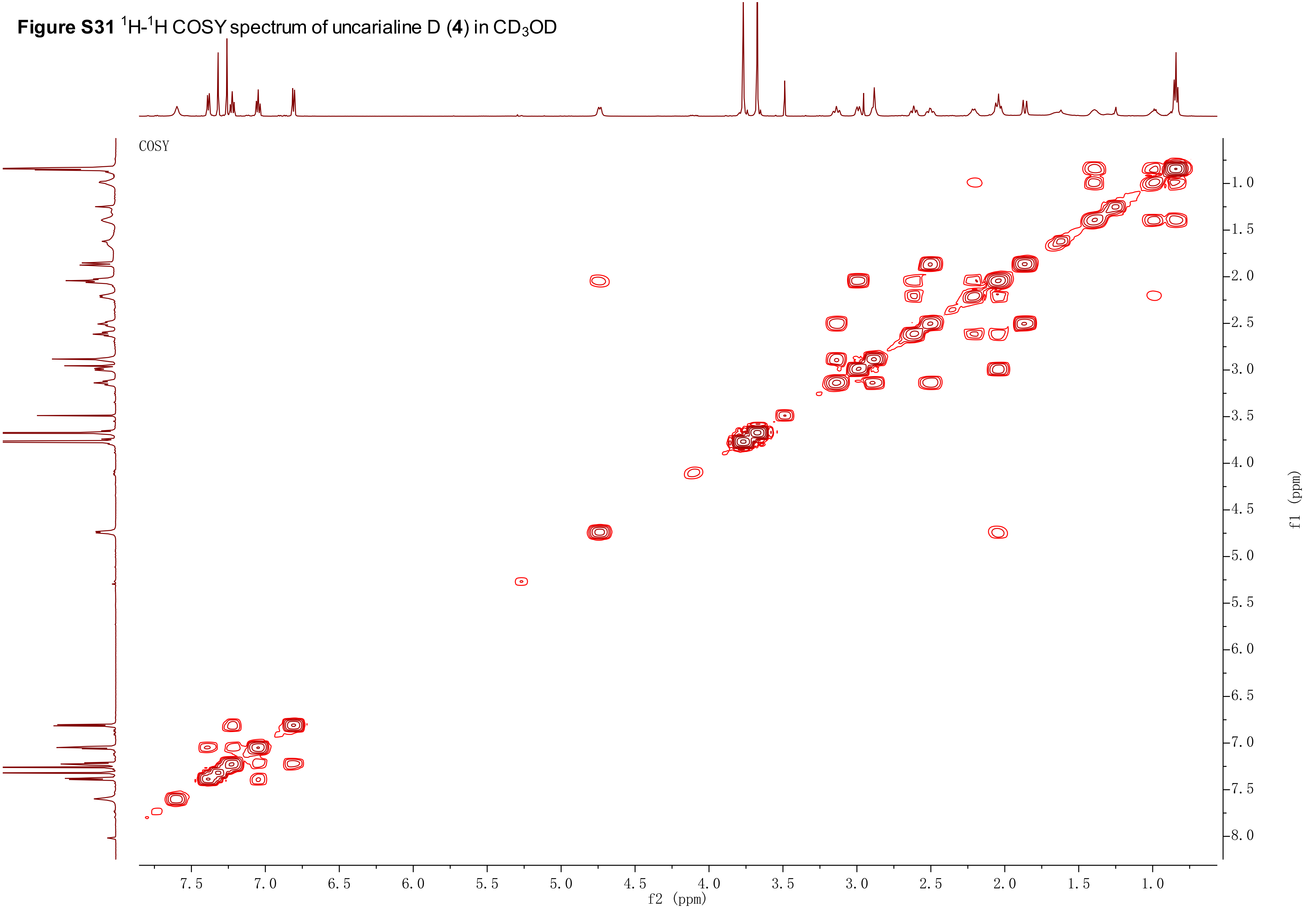

Figure S32 HMBC spectrum of uncarialine D (**4**) in CD_3OD

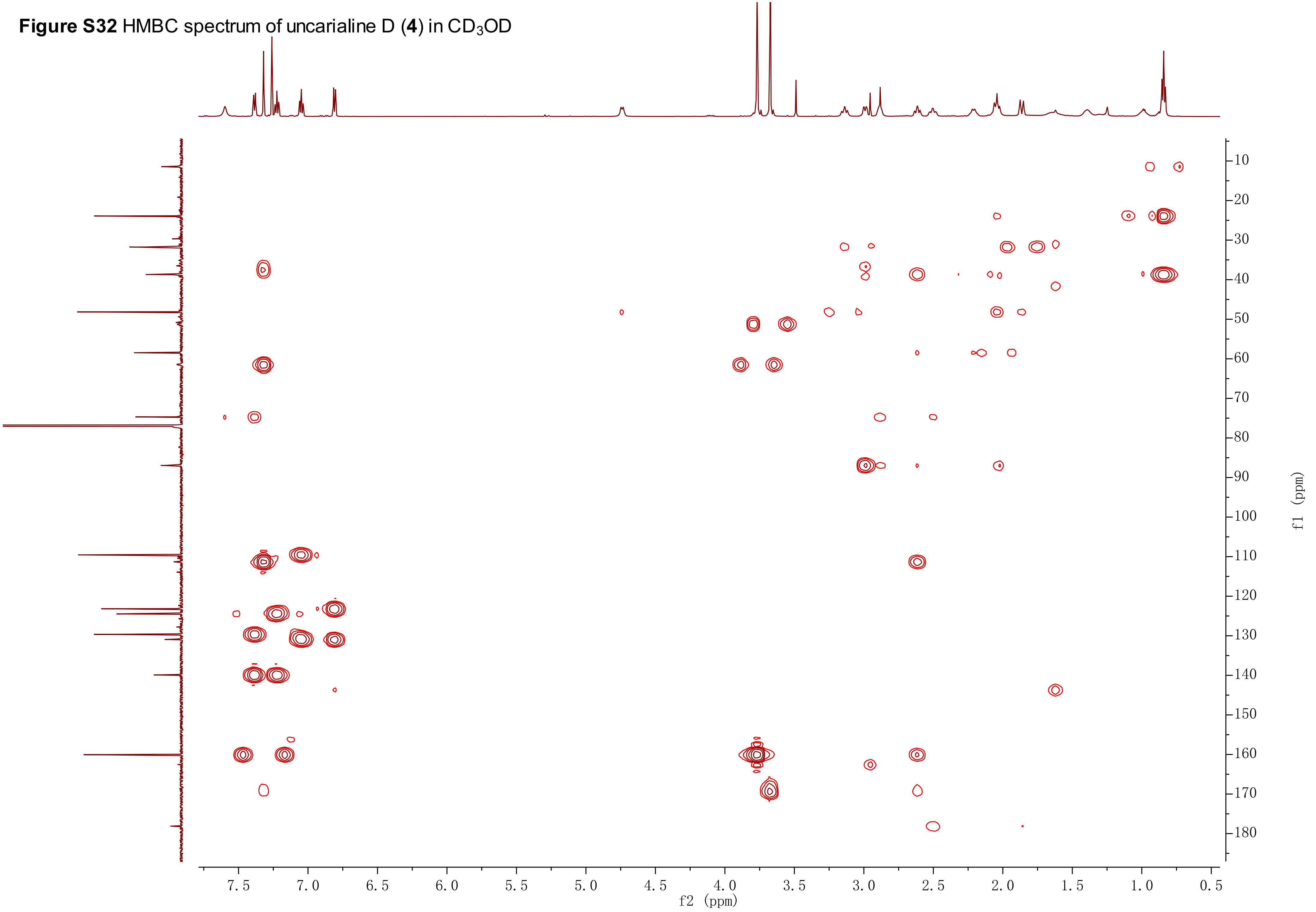

Figure S33 ROESY spectrum of uncarialine D (**4**) in CD_3OD

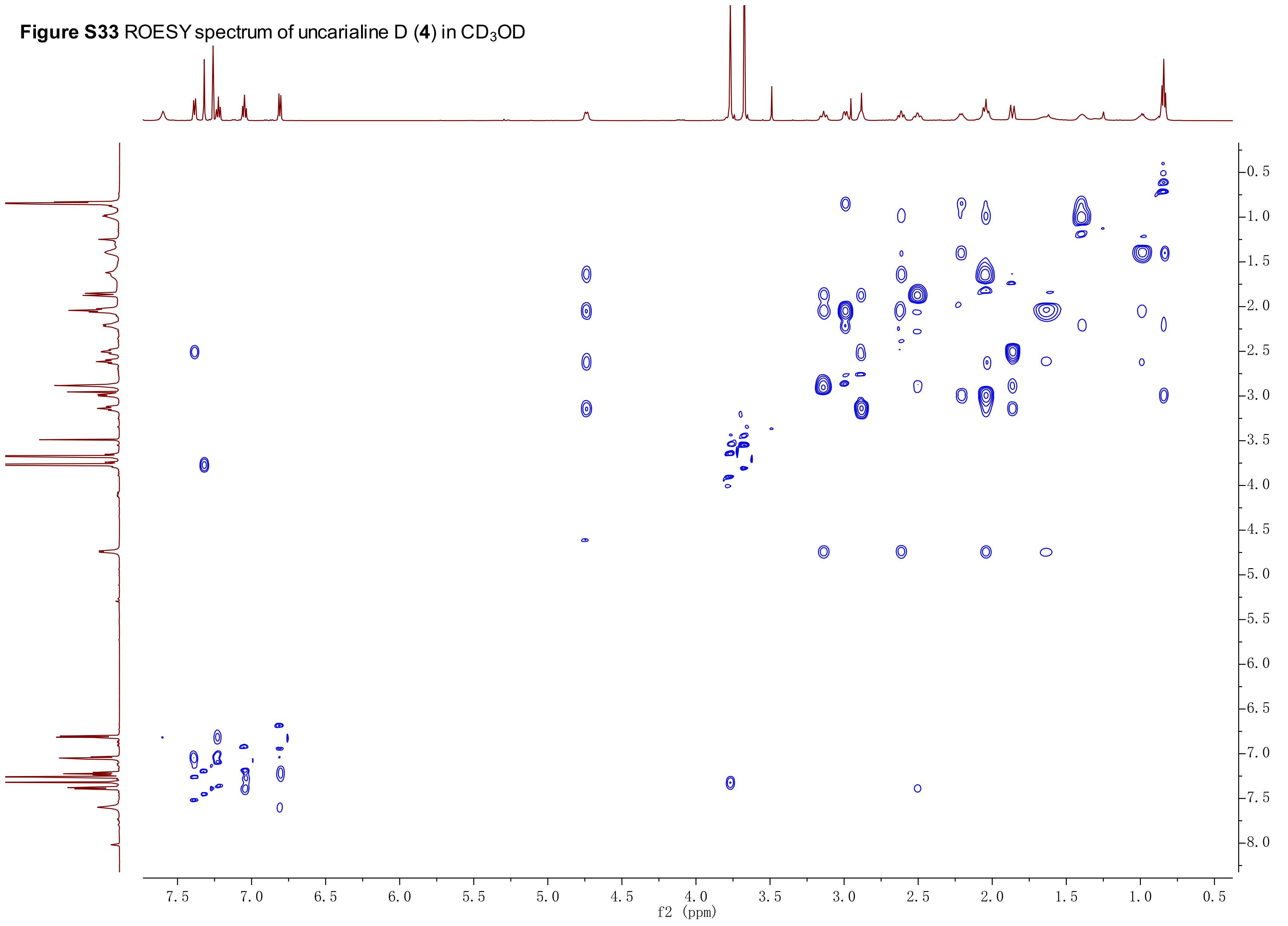

Figure S34 HRESIMS spectrum of uncarialine D (4)

Qualitative Analysis Report

**Data Filename**	HKP-18.d	**Sample Name**	HKP-18
**Sample Type**	Sample	**Position**	P1-E4
**Instrument Name**	Instrument 1	**User Name**	
**Acq Method**	s.m	**Acquired Time**	3/2/2022 2:47:04 PM
**IRM Calibration Status**	Success	**DA Method**	PCDL.m
**Comment**			

**Sample Group**		**Info.**
**Acquisition SW Version**	6200 series TOF/6500 series Q-TOF B.05.01 (B5125.2)	

User Spectra

Fragmentor Voltage	Collision Energy	Ionization Mode
135	0	ESI

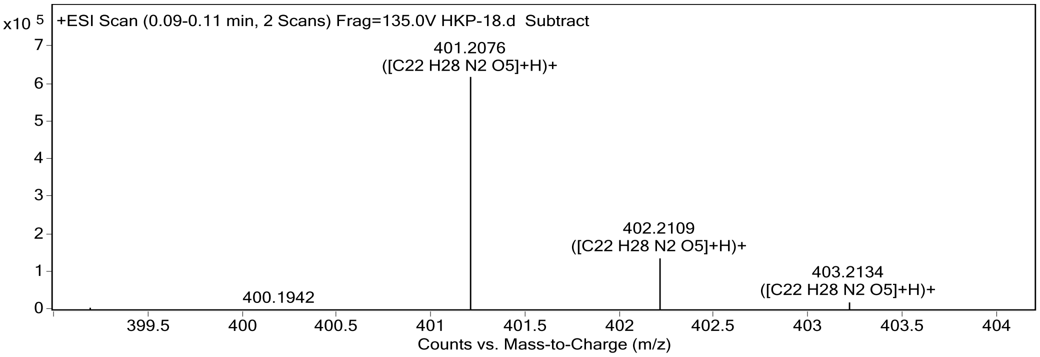

Peak List

m/z	z	Abund	Formula	Ion
102.1278	1	3538.57		
121.0509	1	1544.46		
399.1917	1	6119.96		
400.1942	1	1623.26		
401.2076	1	619883.56	C22 H28 N2 O5	(M+H)+
402.2109	1	139138.36	C22 H28 N2 O5	(M+H)+
403.2134	1	22415.71	C22 H28 N2 O5	(M+H)+
404.2162	1	2511.22	C22 H28 N2 O5	(M+H)+
423.1889	1	8010.07		
424.1932	1	1861.24		

Formula Calculator Element Limits

Element	Min	Max
C	3	60
H	0	120
O	0	30
N	0	5

Formula Calculator Results

Formula	CalculatedMass	CalculatedMz	Mz	Diff. (mDa)	Diff. (ppm)	DBE
C22 H28 N2 O5	400.1998	401.2071	401.2076	-0.50	-1.25	10.0000

--- End Of Report ---

Figure S35 IR spectrum of uncarialine D (**4**)

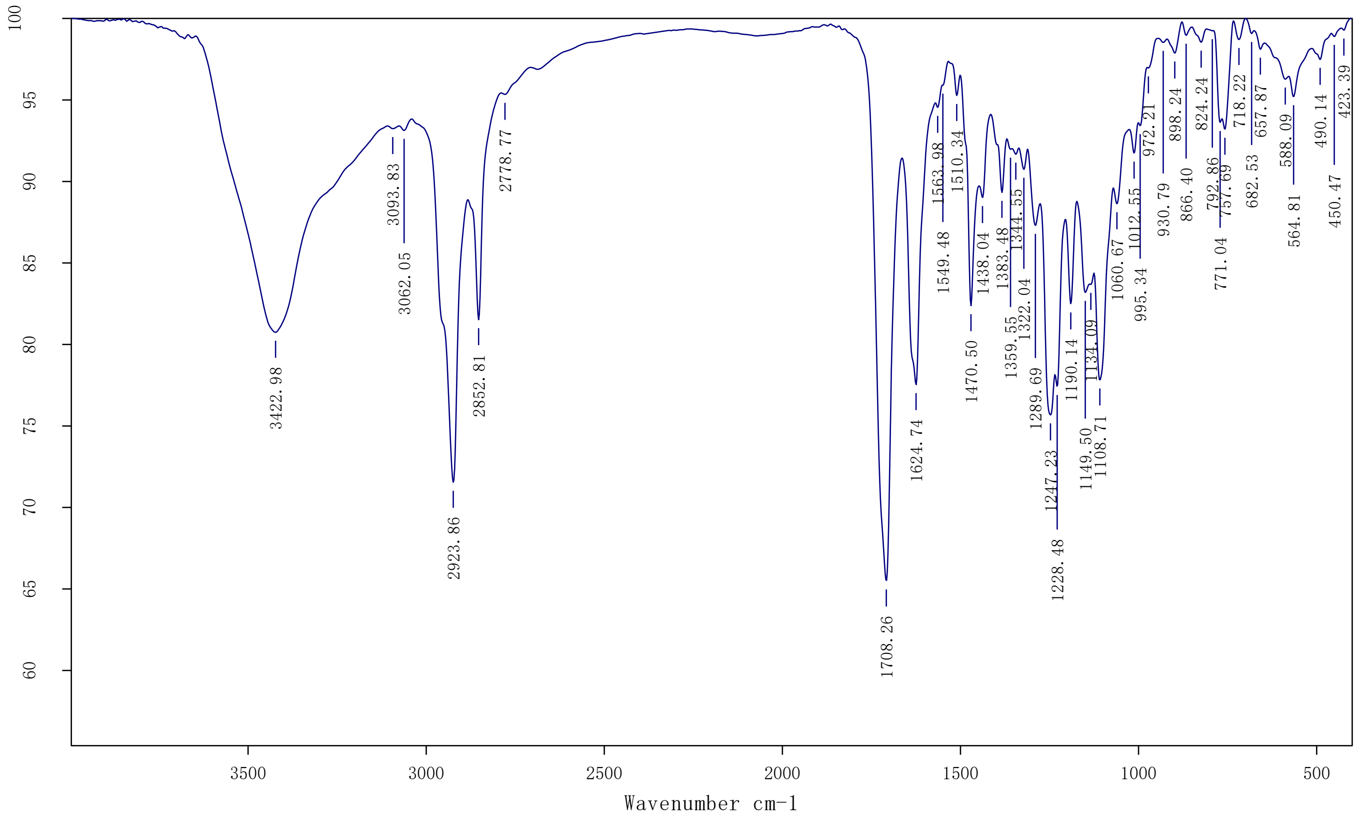

Sample Name: HKP 18a
Sample Form: KBr
Path of File: E:\data
Date of Measurement: 2022/5/31

Resolution: 4
Aperture Setting: 6 mm
Number of Background Scans: 16
Number of Sample Scans: 16

Beamsplitter Setting: KBr
Source Setting: MIR
Instrument Type: BRUKER VERTEX 70
Soft Version: OPUS8.1

Figure S36 ECD spectrum of uncarialine D (**4**)

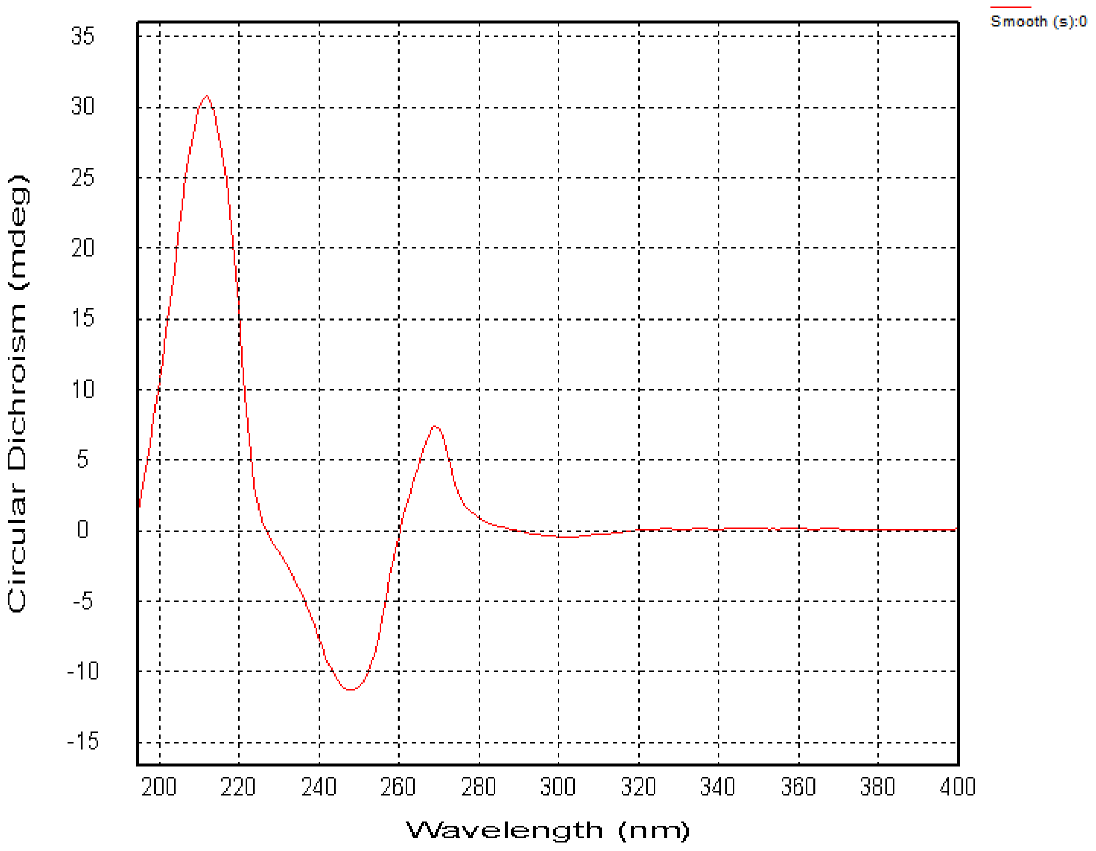

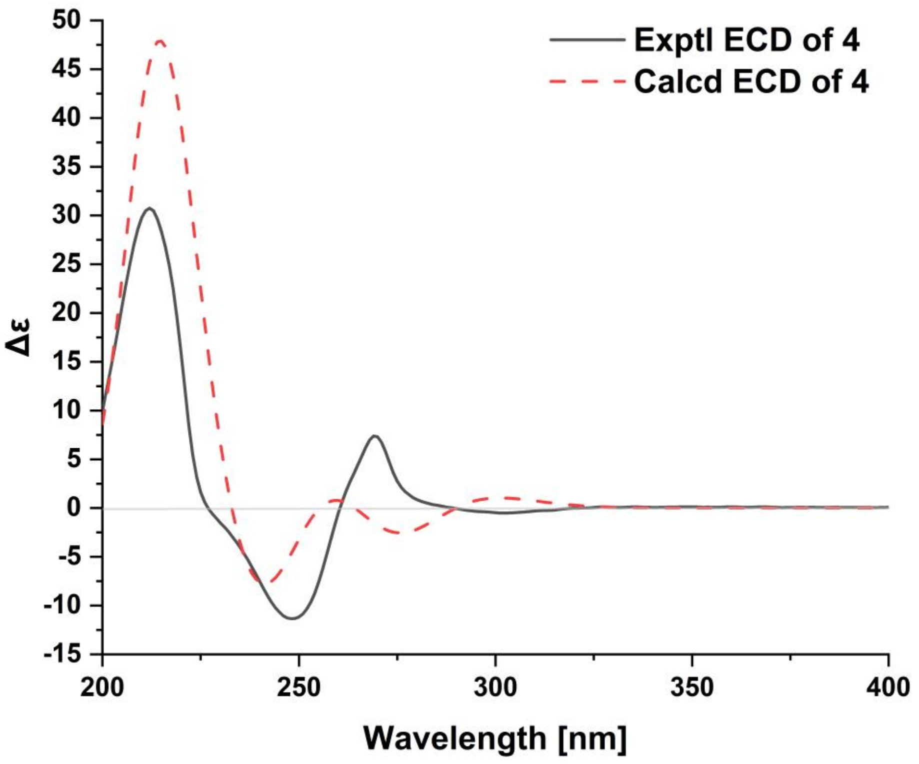

Figure S37 ^{1}H NMR spectrum of uncarialine E (**5**) in $CDCl_3$

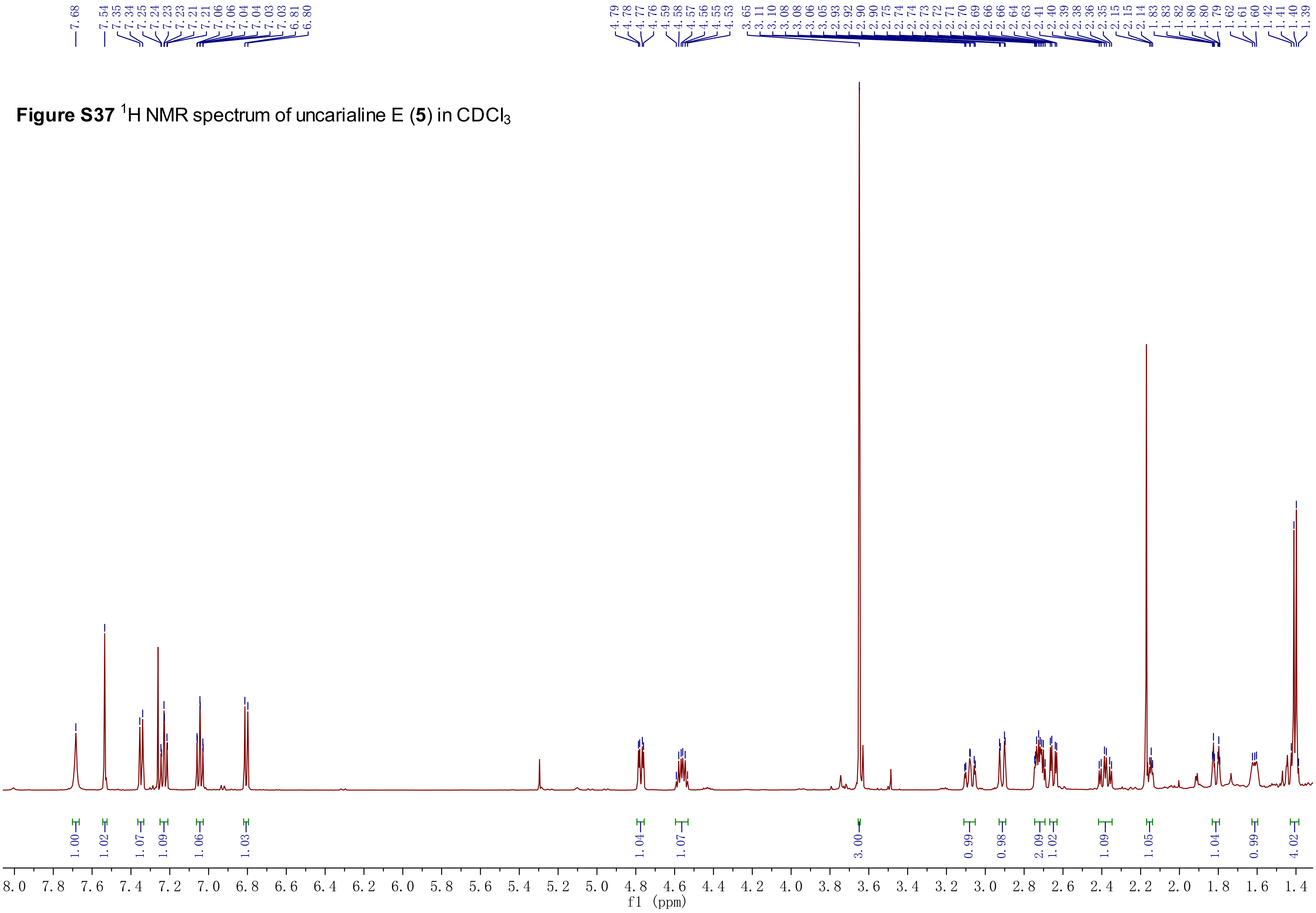

Figure S38 ^{13}C NMR spectrum of uncarialine E (**5**) in $CDCl_3$

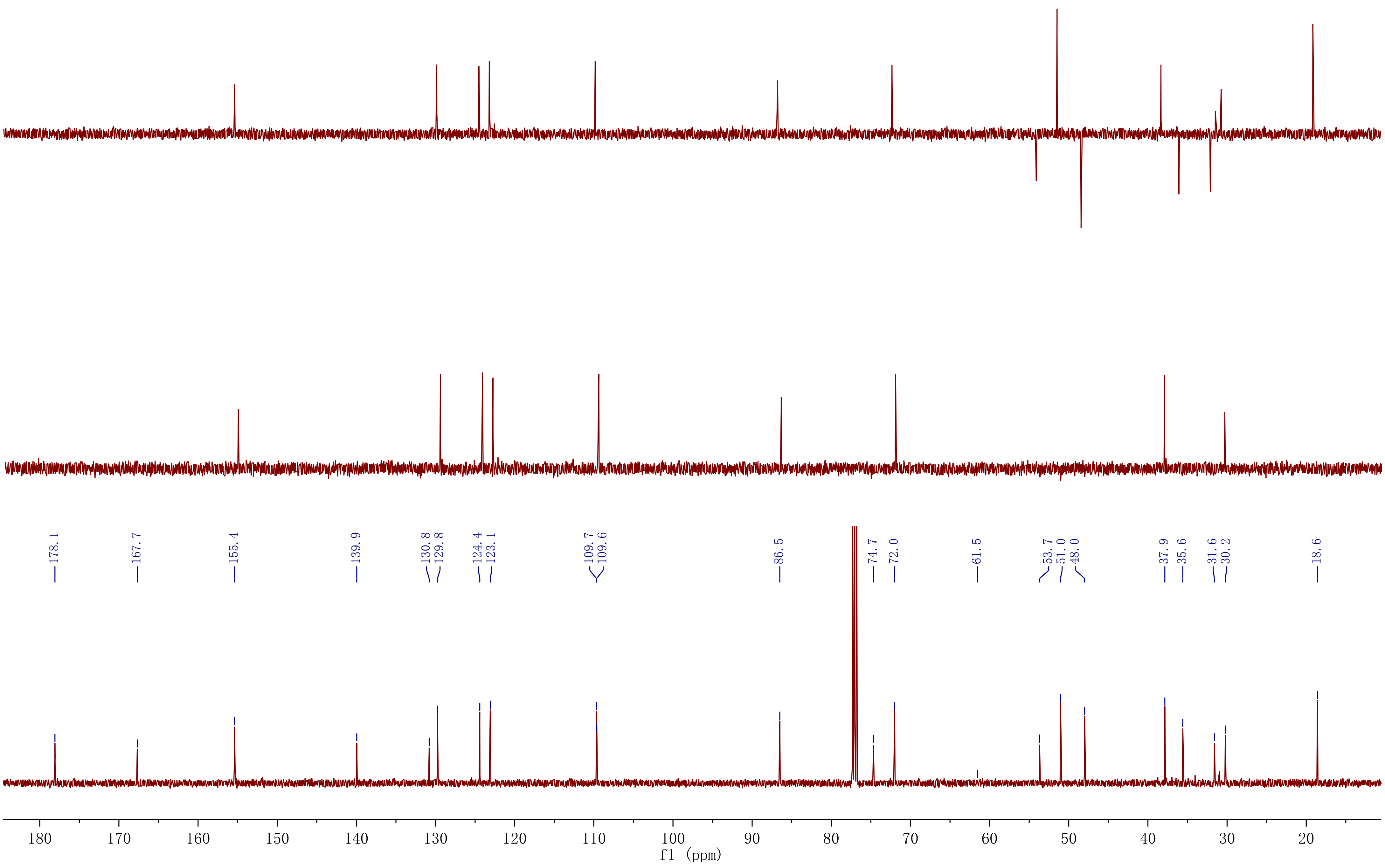

Figure S39 HSQC spectrum of uncarialine E (**5**) in $CDCl_3$

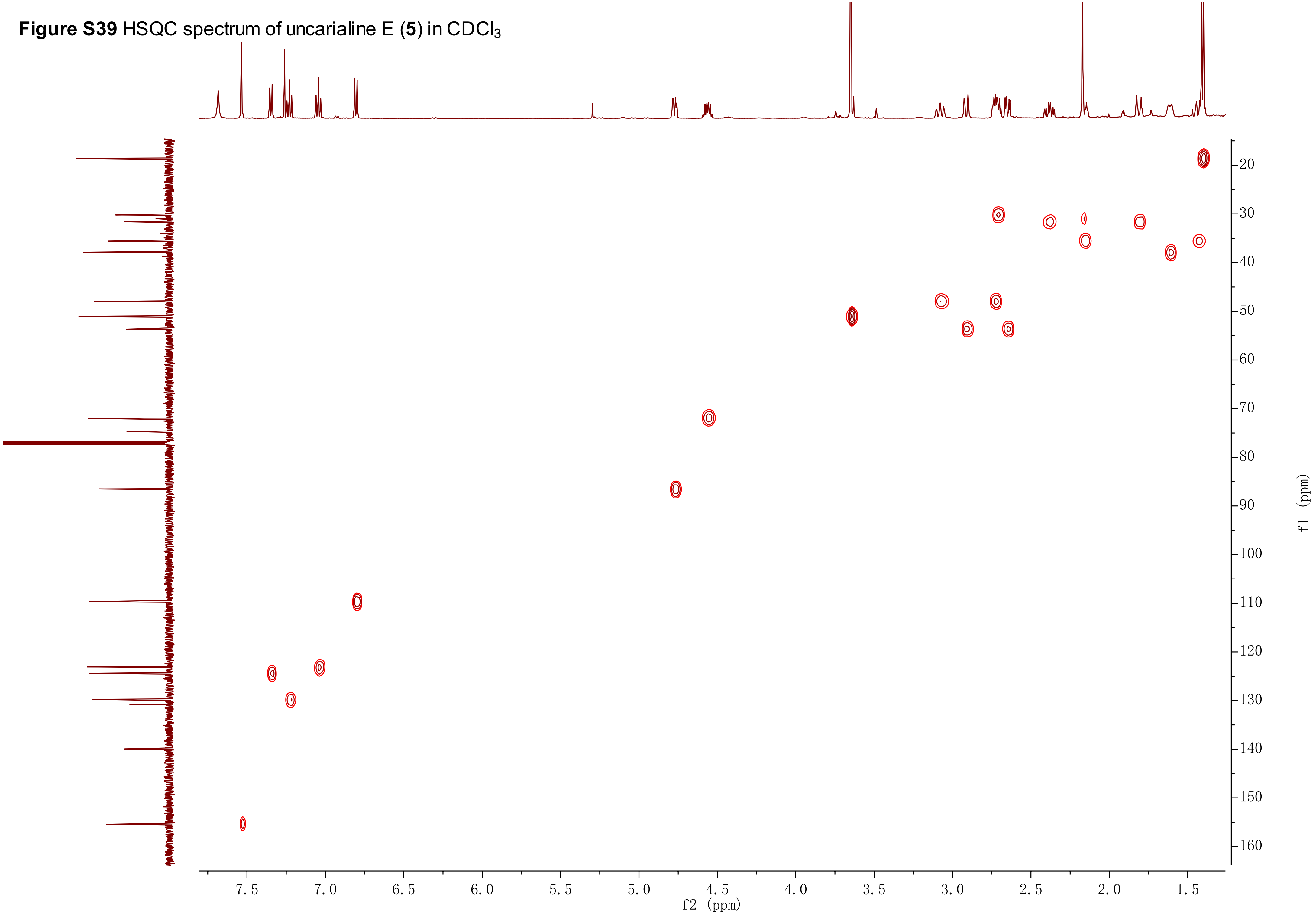

Figure S40 ^{1}H-^{1}H COSY spectrum of uncarialine E (**5**) in $CDCl_3$

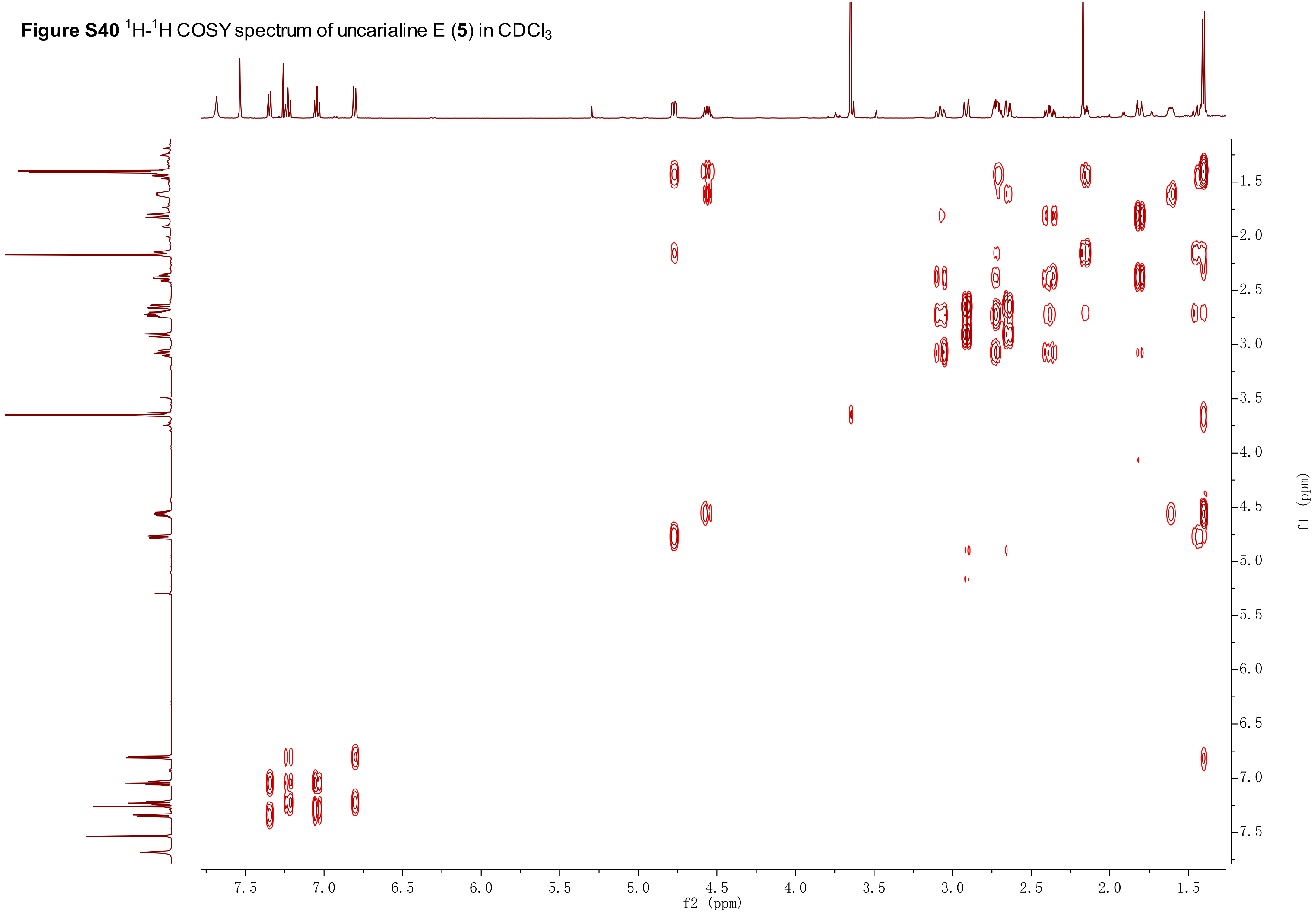

Figure S41 HMBC spectrum of uncarialine E (**5**) in $CDCl_3$

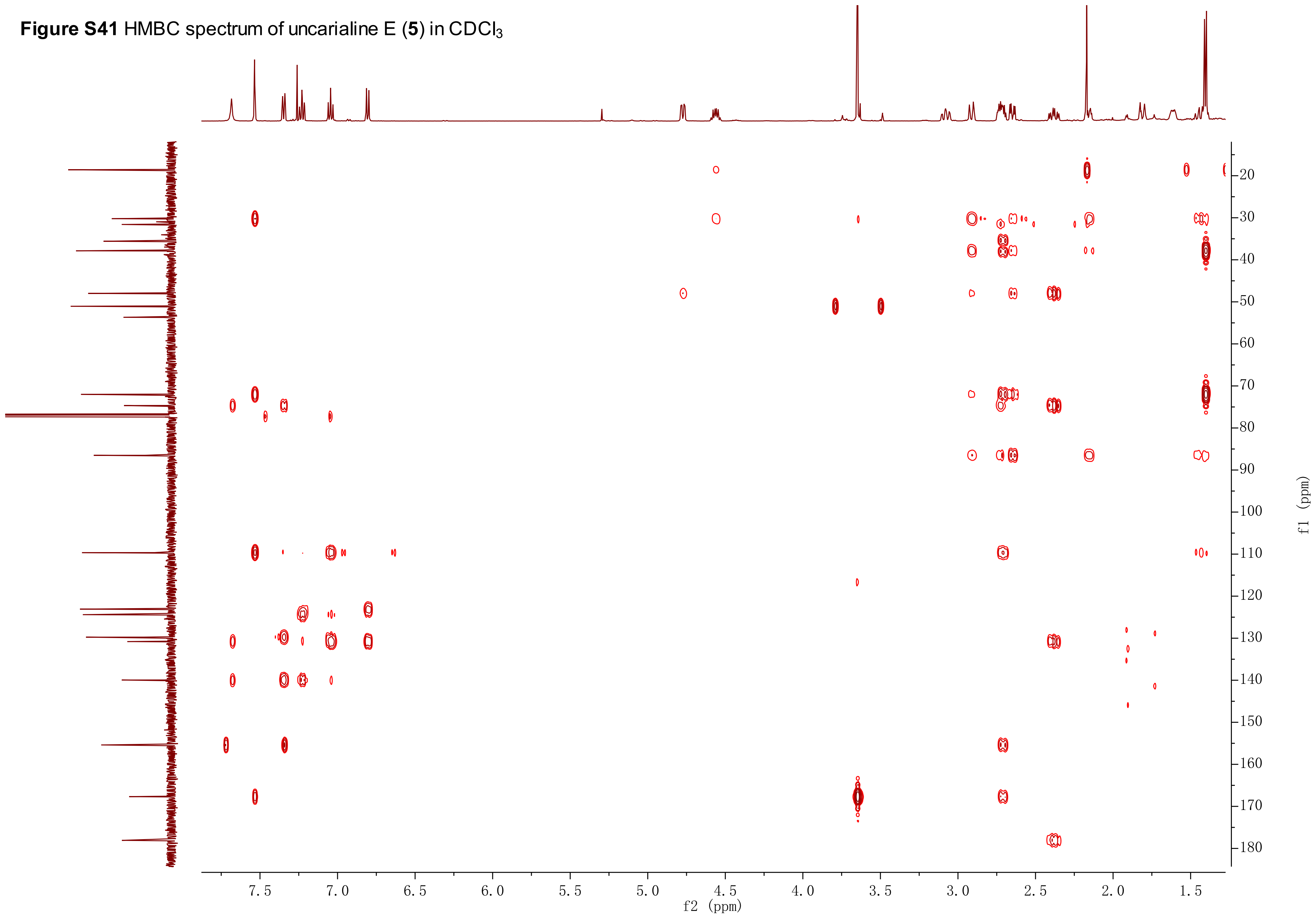

Figure S42 ROESY spectrum of uncarialine E (**5**) in $CDCl_3$

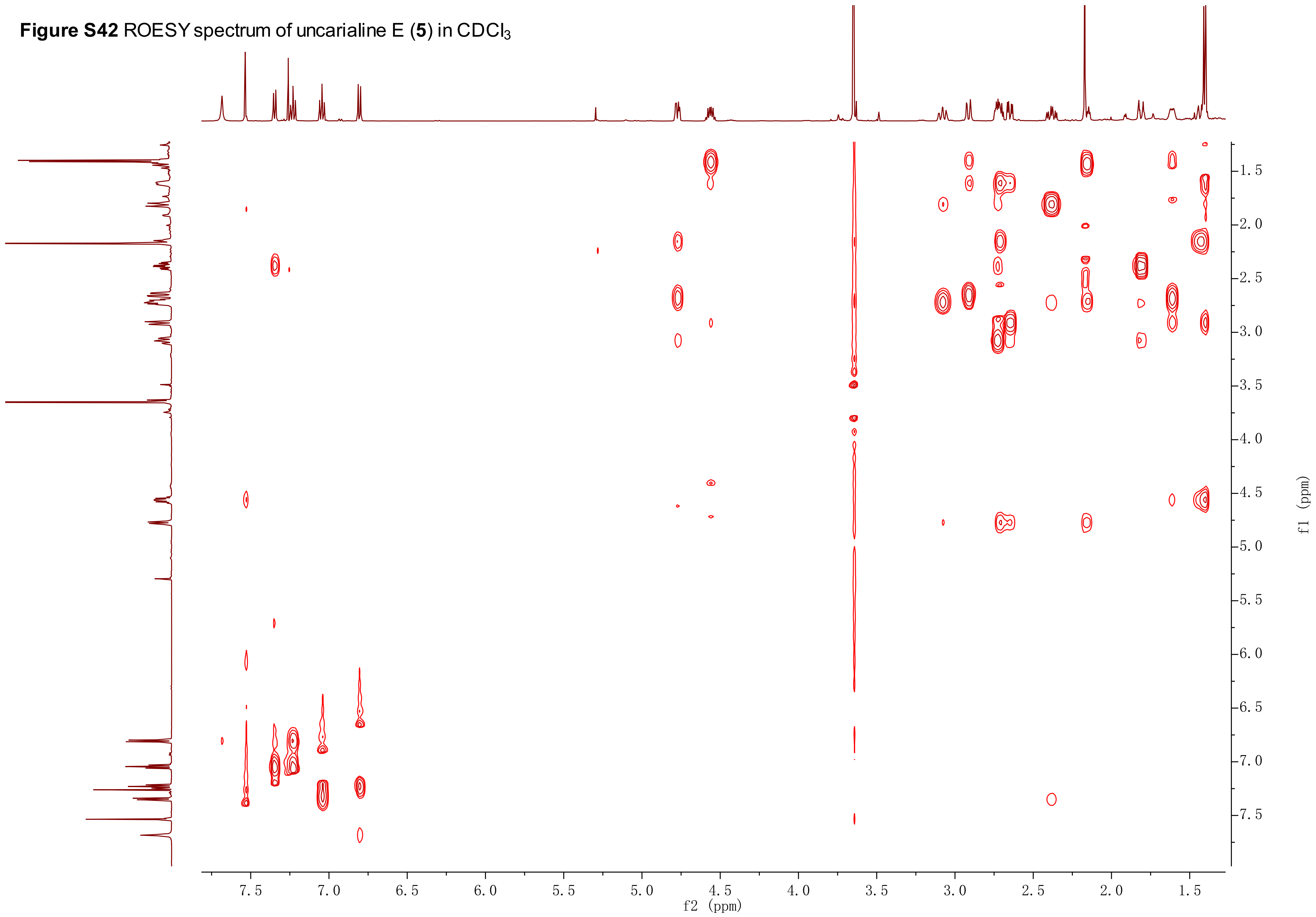

Figure S43 HRESIMS spectrum of uncarialine E (**5**)

Qualitative Analysis Report

**Data Filename**	HUR4.d	**Sample Name**	HUR4
**Sample Type**	Sample	**Position**	P1-A1
**Instrument Name**	Instrument 1	**User Name**	
**Acq Method**	s.m	**Acquired Time**	12/15/2020 11:01:47 AM
**IRM Calibration Status**	Success	**DA Method**	PCDL.m
**Comment**			

Sample Group **Info.**

Acquisition SW 6200 series TOF/6500 series

Version Q-TOF B.05.01 (B5125.2)

User Spectra

Fragmentor Voltage	Collision Energy	Ionization Mode
175	0	ESI

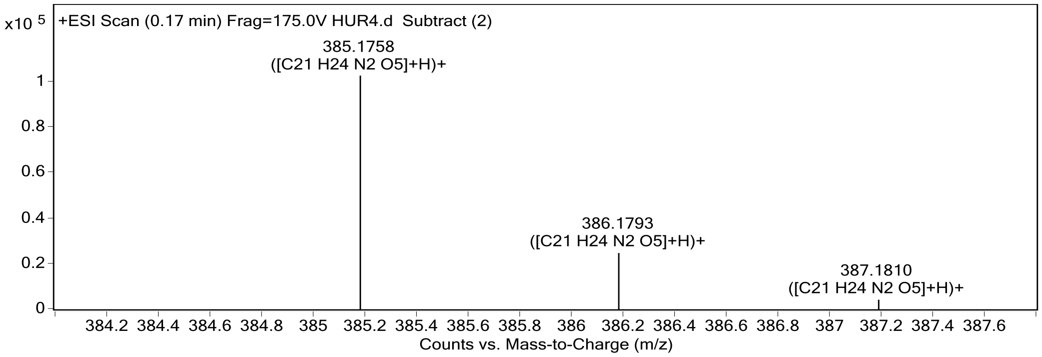

Peak List

m/z	z	Abund	Formula	Ion
102.1279	1	2534.14		
385.1758	1	102998.01	C21 H24 N2 O5	(M+H)+
386.1793	1	25163.49	C21 H24 N2 O5	(M+H)+
387.181	1	4490.22	C21 H24 N2 O5	(M+H)+
407.1576	1	30387.37		
408.1611	1	6742.94		
423.1315	1	18362.01		
424.1357	1	4402.51		
537.3945	1	3013.6		
553.3776	1	2762.65		

Formula Calculator Element Limits

Element	Min	Max
C	3	60
H	0	120
O	0	30
N	0	3

Formula Calculator Results

Formula	CalculatedMass	CalculatedMz	Mz	Diff. (mDa)	Diff. (ppm)	DBE
C21 H24 N2 O5	384.1685	385.1758	385.1758	0.00	0.00	11.0000

--- End Of Report ---

Figure S44 IR spectrum of uncarialine E (**5**)

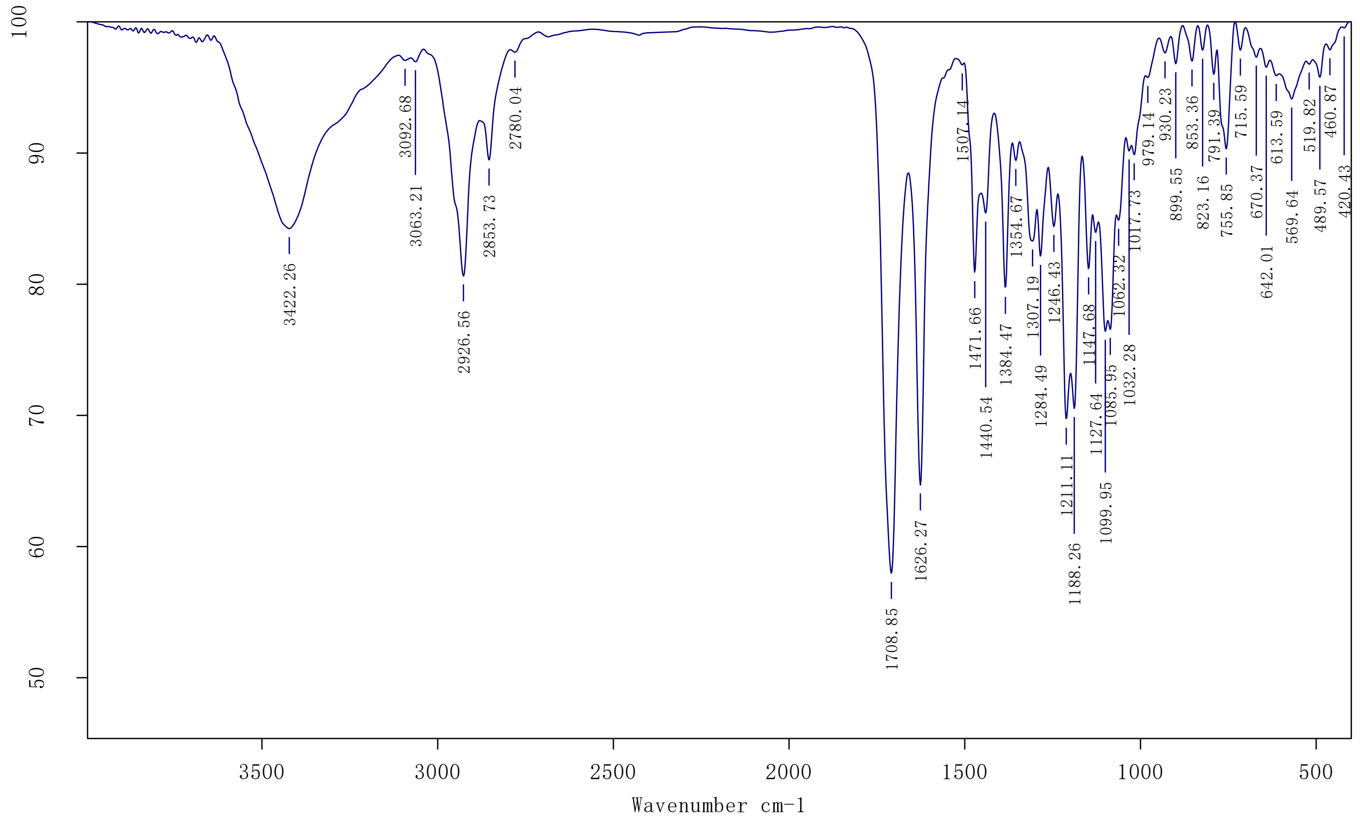

Sample Name: HUR 4
Sample Form: KBr
Path of File: E:\data
Date of Measurement: 2022/7/11

Resolution: 4
Aperture Setting: 6 mm
Number of Background Scans: 16
Number of Sample Scans: 16

Beamsplitter Setting: KBr
Source Setting: MIR
Instrument Type: BRUKER VERTEX 70
Soft Version: OPUS8.1

Figure S45 ECD spectrum of uncarialine E (**5**)

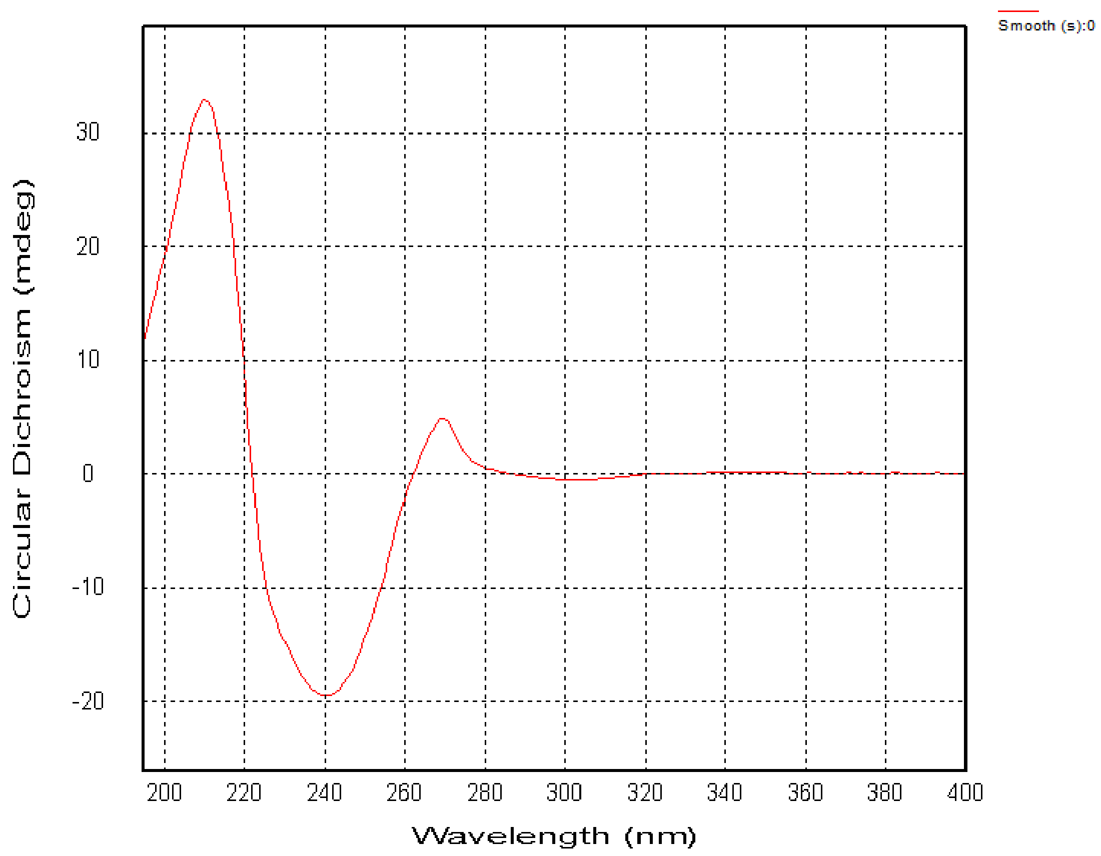

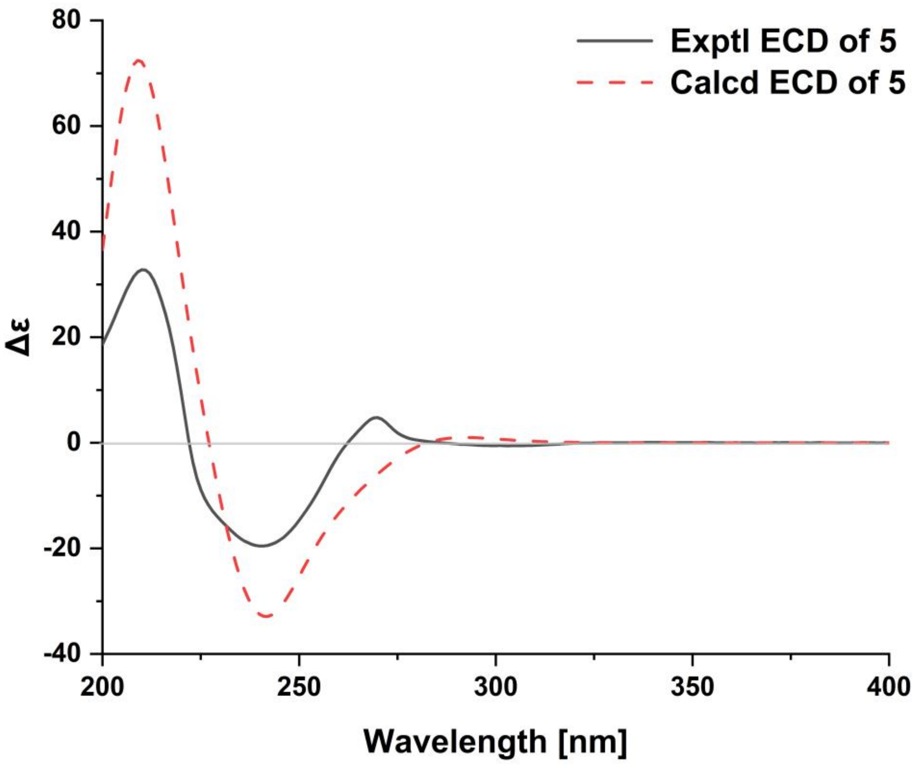

2. Computational methods for ECD calculation of **1-5**

The CONFLEX[1, 2] searches based on molecular mechanics with MMFF94S force fields were performed for **1**, **2**, **3**, **4** and **5** which gave 91, 110, 38, 32 and 2 stable conformers, respectively. Selected conformers (18, 19, 11, 18 and 2) with distributions higher than 1% were further optimized by the density functional theory method at the B3LYP/6-31G* level in Gaussian 09 program package,[3] leading to 4, 4, 4, 2 and 2 geometries (ΔE>2 kcal/mol), respectively, which was further checked by frequency TD-DFT-B3LYP/6-31G (d, p) of theory for compounds **1**, **2**, **3**, **4** and **5** on B3LYP/6-31G(d) optimized geometry through the IEFPCM model (in MeOH). The calculated ECD curves were generated using SpecDis 1.60[4].

Standard orientation of **1a** at B3LYP/6-31G(d) level in gas phase:

Center Number	Atomic Number	Atomic Type	Coordinates (Angstroms) X	Y	Z
1	6	0	5.169718	1.622292	-0.962727
2	6	0	4.793901	2.553072	0.017097
3	6	0	3.737565	2.309894	0.864811
4	6	0	3.054074	1.104969	0.714560
5	6	0	3.415744	0.158613	-0.251674
6	6	0	4.491169	0.433165	-1.102070
7	7	0	1.979661	0.604397	1.396795
8	6	0	1.674170	-0.648656	0.911866
9	6	0	0.536836	-1.409680	1.543680
10	7	0	0.501793	-1.474211	-2.266599
11	6	0	1.824133	-2.060734	-2.305666
12	6	0	2.529049	-2.221272	-0.938573
13	6	0	2.518700	-0.970644	-0.099824
14	6	0	-0.856626	-0.780865	1.350296
15	6	0	-1.293290	-0.271503	-0.046691
16	6	0	-2.413289	0.746633	0.134419
17	6	0	-2.393069	-0.785088	-2.281282
18	6	0	-1.693453	-1.375344	-1.076846
19	6	0	-0.509495	-2.237292	-1.553849
20	6	0	-2.261180	2.034429	-0.158037
21	1	0	-0.470761	0.298990	-0.453683

22	6	0	-3.597814	-1.132281	-2.690984
23	1	0	-2.387767	-2.046252	-0.586242
24	6	0	-3.722465	0.295065	0.657181
25	8	0	-3.963513	-0.827793	0.982857
26	8	0	-4.633758	1.261968	0.745204
27	6	0	-5.909868	0.892044	1.232036
28	8	0	0.704984	-1.448972	2.943144
29	6	0	1.700817	-2.313015	3.405108
30	8	0	-1.119884	2.535969	-0.635132
31	6	0	-1.109059	3.906398	-0.946641
32	1	0	5.999583	1.844643	-1.609472
33	1	0	5.342812	3.473786	0.106649
34	1	0	3.450286	3.024783	1.615804
35	1	0	4.790016	-0.275080	-1.854794
36	1	0	1.605478	0.970814	2.240875
37	1	0	0.551914	-2.422752	1.164846
38	1	0	0.577479	-0.550396	-1.897185
39	1	0	1.762156	-3.032722	-2.787235
40	1	0	2.435857	-1.434509	-2.946947
41	1	0	3.557385	-2.524454	-1.121046
42	1	0	2.078650	-3.037650	-0.385186
43	1	0	-0.880311	0.070915	2.021696
44	1	0	-1.582887	-1.485459	1.734220
45	1	0	-1.836672	-0.055018	-2.846242
46	1	0	-0.104999	-2.775168	-0.702052
47	1	0	-0.901791	-2.994663	-2.226052
48	1	0	-3.061058	2.737392	-0.021068
49	1	0	-4.037621	-0.703518	-3.574644
50	1	0	-4.191578	-1.857276	-2.160348
51	1	0	-6.498951	1.796363	1.228601
52	1	0	-6.360765	0.149614	0.589936
53	1	0	-5.835610	0.497542	2.234983
54	1	0	1.489834	-3.341583	3.121140
55	1	0	2.681056	-2.041034	3.025822
56	1	0	1.707557	-2.243494	4.484232
57	1	0	-0.115174	4.135402	-1.298506
58	1	0	-1.828115	4.133089	-1.726182
59	1	0	-1.326426	4.505820	-0.069010

Standard orientation of **1b** at B3LYP/6-31G(d) level in gas phase:

Center Number	Atomic Number	Atomic Type	Coordinates (Angstroms) X	Y	Z

1	6	0	5.298564	1.571379	-0.644574
2	6	0	4.927200	2.399605	0.423778
3	6	0	3.839305	2.101049	1.213076
4	6	0	3.122146	0.945851	0.913053
5	6	0	3.481372	0.099055	-0.141499
6	6	0	4.585870	0.428822	-0.931654
7	7	0	2.005825	0.417369	1.505910
8	6	0	1.689490	-0.773728	0.887554
9	6	0	0.520292	-1.567065	1.399497
10	7	0	0.588791	-1.242758	-2.403447
11	6	0	1.899076	-1.855215	-2.461316
12	6	0	2.554301	-2.169479	-1.096801
13	6	0	2.547838	-1.011915	-0.133706
14	6	0	-0.852236	-0.877371	1.227743
15	6	0	-1.235043	-0.219602	-0.123329
16	6	0	-2.332108	0.808316	0.128372
17	6	0	-2.288132	-0.480663	-2.423526
18	6	0	-1.635282	-1.204944	-1.267012
19	6	0	-0.459614	-2.046453	-1.797505
20	6	0	-2.140446	2.112574	-0.045113
21	1	0	-0.385727	0.363054	-0.449092
22	6	0	-3.489330	-0.754310	-2.894752
23	1	0	-2.359199	-1.901976	-0.862537
24	6	0	-3.663976	0.348318	0.580102
25	8	0	-3.939681	-0.791698	0.805322
26	8	0	-4.554725	1.326736	0.727486
27	6	0	-5.851760	0.950330	1.149598
28	8	0	0.748988	-1.754669	2.781510
29	6	0	0.156885	-2.879191	3.359445
30	8	0	-0.978730	2.621444	-0.458278
31	6	0	-0.911880	4.017494	-0.607576
32	1	0	6.152480	1.834475	-1.243014
33	1	0	5.503261	3.284449	0.629450
34	1	0	3.554040	2.738158	2.031820
35	1	0	4.882759	-0.201988	-1.751094
36	1	0	1.683491	0.648531	2.417203
37	1	0	0.530698	-2.533017	0.914165
38	1	0	0.673986	-0.362089	-1.942152
39	1	0	1.831858	-2.771983	-3.040676
40	1	0	2.544283	-1.182882	-3.017413
41	1	0	3.581042	-2.478904	-1.277509
42	1	0	2.069307	-3.026263	-0.642814
43	1	0	-0.867037	-0.092395	1.976744

44	1	0	-1.624706	-1.581011	1.510196
45	1	0	-1.699680	0.286756	-2.899358
46	1	0	-0.092157	-2.676584	-0.993353
47	1	0	-0.850388	-2.723014	-2.551815
48	1	0	-2.924446	2.822507	0.139748
49	1	0	-3.895241	-0.229279	-3.741888
50	1	0	-4.113868	-1.512738	-2.453604
51	1	0	-6.419551	1.866395	1.206078
52	1	0	-6.301900	0.274702	0.437100
53	1	0	-5.814196	0.473204	2.118152
54	1	0	-0.927338	-2.828039	3.345055
55	1	0	0.470563	-3.790594	2.855498
56	1	0	0.488315	-2.919431	4.388000
57	1	0	0.090568	4.245232	-0.934847
58	1	0	-1.621174	4.362540	-1.351910
59	1	0	-1.104364	4.518238	0.335170

Standard orientation of **1c** at B3LYP/6-31G(d) level in gas phase:

Center Number	Atomic Number	Atomic Type	Coordinates (Angstroms) X	Y	Z
1	6	0	-5.234167	1.436482	0.652453
2	6	0	-4.937176	2.154043	-0.515883
3	6	0	-3.865364	1.814641	-1.309120
4	6	0	-3.084511	0.731885	-0.909301
5	6	0	-3.365646	-0.002198	0.249069
6	6	0	-4.459706	0.366483	1.038113
7	7	0	-1.973811	0.179780	-1.485167
8	6	0	-1.564110	-0.904187	-0.740124
9	6	0	-0.364324	-1.688881	-1.204628
10	7	0	-0.334247	-0.902096	2.535368
11	6	0	-1.602978	-1.574109	2.717788
12	6	0	-2.284575	-2.090268	1.428961
13	6	0	-2.377772	-1.060308	0.334205
14	6	0	0.969107	-0.915972	-1.174846
15	6	0	1.342951	-0.070541	0.068932
16	6	0	2.326223	1.021568	-0.338585
17	6	0	2.493972	0.019898	2.335430
18	6	0	1.843708	-0.879180	1.307898
19	6	0	0.740195	-1.712670	1.987549
20	6	0	2.006725	2.312145	-0.298595
21	1	0	0.455661	0.474017	0.356984

22	6	0	3.716915	-0.139198	2.802726
23	1	0	2.591891	-1.584333	0.965569
24	6	0	3.707356	0.757575	-0.810402
25	8	0	4.491424	1.598382	-1.138597
26	8	0	4.007422	-0.534206	-0.847446
27	6	0	5.311410	-0.875200	-1.279674
28	8	0	-0.519321	-2.055300	-2.557034
29	6	0	-1.445397	-3.072378	-2.802633
30	8	0	0.817128	2.756519	0.105935
31	6	0	0.628893	4.150395	0.116043
32	1	0	-6.079077	1.729321	1.249702
33	1	0	-5.560256	2.984882	-0.795973
34	1	0	-3.638826	2.365814	-2.204884
35	1	0	-4.698503	-0.178886	1.934243
36	1	0	-1.626602	0.385127	-2.392915
37	1	0	-0.294490	-2.588847	-0.608296
38	1	0	-0.485072	-0.085365	1.982539
39	1	0	-1.462748	-2.407929	3.400169
40	1	0	-2.268592	-0.875408	3.213862
41	1	0	-3.284118	-2.430971	1.688781
42	1	0	-1.764251	-2.968326	1.063024
43	1	0	0.926959	-0.239751	-2.021714
44	1	0	1.753777	-1.627559	-1.398930
45	1	0	1.886211	0.822295	2.720146
46	1	0	0.383906	-2.457480	1.282518
47	1	0	1.198697	-2.267055	2.801142
48	1	0	2.720448	3.057860	-0.597497
49	1	0	4.122537	0.512996	3.556007
50	1	0	4.362058	-0.927770	2.452979
51	1	0	5.361982	-1.952422	-1.239477
52	1	0	5.479484	-0.529657	-2.289187
53	1	0	6.053058	-0.439756	-0.626188
54	1	0	-1.161916	-3.991553	-2.294665
55	1	0	-2.445820	-2.794128	-2.486312
56	1	0	-1.448222	-3.249696	-3.869325
57	1	0	-0.381554	4.322680	0.452689
58	1	0	1.323307	4.629844	0.796809
59	1	0	0.752877	4.564963	-0.878326

Standard orientation of **1d** at B3LYP/6-31G(d) level in gas phase:

Center Number	Atomic Number	Atomic Type	Coordinates (Angstroms) X	Y	Z

1	6	0	5.134433	1.578261	-1.057621
2	6	0	4.780329	2.540893	-0.101604
3	6	0	3.737872	2.328619	0.772115
4	6	0	3.046236	1.123691	0.672106
5	6	0	3.386636	0.145178	-0.270599
6	6	0	4.447701	0.388339	-1.147028
7	7	0	1.984223	0.648797	1.390421
8	6	0	1.666305	-0.617470	0.949258
9	6	0	0.530367	-1.352144	1.610282
10	7	0	0.446917	-1.580526	-2.132807
11	6	0	1.766962	-2.170279	-2.214252
12	6	0	2.478616	-2.253185	-0.853364
13	6	0	2.488108	-0.972306	-0.066290
14	6	0	-0.857724	-0.724977	1.390614
15	6	0	-1.291367	-0.298277	-0.034544
16	6	0	-2.371332	0.770747	0.092798
17	6	0	-2.463896	-0.906215	-2.190532
18	6	0	-1.746662	-1.452950	-0.975310
19	6	0	-0.577935	-2.342082	-1.445228
20	6	0	-2.173976	2.035421	-0.267143
21	1	0	-0.451220	0.181966	-0.513587
22	6	0	-3.631738	-1.329381	-2.635319
23	1	0	-2.440313	-2.091142	-0.440265
24	6	0	-3.690417	0.400421	0.653264
25	8	0	-3.968808	-0.689921	1.051760
26	8	0	-4.568328	1.402721	0.686742
27	6	0	-5.848790	1.109375	1.210888
28	8	0	0.698799	-1.343909	3.010946
29	6	0	1.698279	-2.187058	3.500622
30	8	0	-1.022053	2.467034	-0.783327
31	6	0	-0.939603	3.831509	-1.104218
32	1	0	5.953792	1.775768	-1.725729
33	1	0	5.334515	3.461504	-0.050410
34	1	0	3.467379	3.068051	1.505555
35	1	0	4.730771	-0.344491	-1.882450
36	1	0	1.623333	1.045360	2.226370
37	1	0	0.541886	-2.379148	1.268518
38	1	0	0.134148	-1.329336	-3.048156
39	1	0	1.760714	-3.172304	-2.654569
40	1	0	2.352359	-1.540111	-2.875191
41	1	0	3.501753	-2.571417	-1.039389
42	1	0	2.037241	-3.045881	-0.259348
43	1	0	-0.868707	0.165130	2.010724

44	1	0	-1.587813	-1.398325	1.821731
45	1	0	-1.953377	-0.122632	-2.729549
46	1	0	-0.133418	-2.826721	-0.589615
47	1	0	-0.986183	-3.143635	-2.063948
48	1	0	-2.944958	2.775042	-0.158637
49	1	0	-4.079758	-0.920006	-3.524153
50	1	0	-4.189954	-2.095230	-2.123986
51	1	0	-6.407652	2.031158	1.155110
52	1	0	-6.333449	0.342555	0.624294
53	1	0	-5.774876	0.778517	2.236708
54	1	0	1.490239	-3.226269	3.254722
55	1	0	2.676732	-1.926320	3.108871
56	1	0	1.708259	-2.079289	4.576721
57	1	0	0.052315	3.997278	-1.494974
58	1	0	-1.673120	4.099600	-1.857334
59	1	0	-1.085928	4.448251	-0.223683

Standard orientation of **2a** at B3LYP/6-31G(d) level in gas phase:

Center Number	Atomic Number	Atomic Type	Coordinates (Angstroms) X	Y	Z
1	6	0	5.593642	0.280860	0.688923
2	6	0	5.441245	-1.071026	1.034585
3	6	0	4.311203	-1.771406	0.684032
4	6	0	3.322561	-1.088293	-0.023255
5	6	0	3.445482	0.261404	-0.367405
6	6	0	4.611016	0.947336	-0.005250
7	7	0	2.126649	-1.522522	-0.512893
8	6	0	1.475370	-0.489668	-1.140119
9	6	0	0.152014	-0.813800	-1.815560
10	7	0	0.563014	2.772123	0.345220
11	6	0	1.681521	3.065606	-0.524790
12	6	0	1.988411	2.021196	-1.623785
13	6	0	2.242458	0.635130	-1.085529
14	6	0	-0.965407	0.208367	-1.581754
15	6	0	-1.351815	0.404399	-0.095193
16	6	0	-2.391625	-0.633009	0.320240
17	6	0	-2.019873	1.982855	1.786391
18	6	0	-1.774227	1.862842	0.267797
19	6	0	-0.766587	2.916516	-0.227819
20	6	0	-3.628850	-0.709097	-0.158440
21	1	0	-0.471179	0.186822	0.487969

22	6	0	-2.496415	3.358670	2.256165
23	1	0	-2.709882	2.083177	-0.237605
24	6	0	-2.068716	-1.697918	1.306855
25	8	0	-2.762935	-2.643736	1.542017
26	8	0	-0.922352	-1.493051	1.936054
27	6	0	-0.530275	-2.460392	2.890343
28	8	0	-0.221383	-2.099221	-1.373120
29	6	0	-0.997055	-2.851520	-2.258687
30	8	0	-4.117573	0.135030	-1.072567
31	6	0	-5.479735	-0.004777	-1.392049
32	1	0	6.492655	0.798276	0.972868
33	1	0	6.224853	-1.567238	1.579309
34	1	0	4.196639	-2.809828	0.941394
35	1	0	4.743983	1.982394	-0.265530
36	1	0	1.712290	-2.414195	-0.382812
37	1	0	0.337188	-0.862127	-2.888395
38	1	0	0.694112	1.876628	0.764637
39	1	0	1.508431	4.030219	-0.993561
40	1	0	2.560967	3.180972	0.099371
41	1	0	2.873271	2.357624	-2.161214
42	1	0	1.192521	2.008261	-2.358235
43	1	0	-0.639964	1.134309	-2.023991
44	1	0	-1.832198	-0.092491	-2.156021
45	1	0	-2.764652	1.248578	2.073049
46	1	0	-1.114279	1.720427	2.326116
47	1	0	-0.734884	2.898002	-1.312988
48	1	0	-1.151905	3.900149	0.021959
49	1	0	-4.293695	-1.483847	0.177048
50	1	0	-2.789239	3.317939	3.301102
51	1	0	-1.719790	4.109005	2.166038
52	1	0	-3.359486	3.695459	1.687019
53	1	0	0.422875	-2.127338	3.270651
54	1	0	-1.253900	-2.520346	3.690299
55	1	0	-0.432231	-3.431718	2.427379
56	1	0	-1.187625	-3.803558	-1.783704
57	1	0	-1.947847	-2.375353	-2.471406
58	1	0	-0.469089	-3.019025	-3.194323
59	1	0	-5.696540	0.725263	-2.156678
60	1	0	-5.688698	-0.998281	-1.773791
61	1	0	-6.102455	0.186803	-0.525470

Standard orientation of **2b** at B3LYP/6-31G(d) level in gas phase:

Center Number	Atomic Number	Atomic Type	Coordinates (Angstroms) X	Y	Z
1	6	0	5.099936	1.222343	-1.446146
2	6	0	4.704373	2.442485	-0.878574
3	6	0	3.647681	2.511523	0.000378
4	6	0	2.983976	1.325131	0.305910
5	6	0	3.365374	0.095011	-0.243762
6	6	0	4.440951	0.054509	-1.136161
7	7	0	1.914993	1.093451	1.126653
8	6	0	1.630844	-0.254404	1.136847
9	6	0	0.498504	-0.748738	1.999354
10	7	0	0.511653	-2.201846	-1.551123
11	6	0	1.832237	-2.753714	-1.339369
12	6	0	2.516329	-2.384725	-0.002250
13	6	0	2.484783	-0.912988	0.314193
14	6	0	-0.906239	-0.276257	1.570850
15	6	0	-1.305666	-0.257277	0.070261
16	6	0	-2.392109	0.798715	-0.110856
17	6	0	-2.350403	-1.472493	-1.938064
18	6	0	-1.699382	-1.636499	-0.548674
19	6	0	-0.511715	-2.610061	-0.603206
20	6	0	-2.201145	1.907437	-0.819729
21	1	0	-0.460591	0.129241	-0.481694
22	6	0	-2.692287	-2.786064	-2.643349
23	1	0	-2.435577	-2.090065	0.106753
24	6	0	-3.705337	0.604528	0.544999
25	8	0	-3.996988	-0.357188	1.188769
26	8	0	-4.561838	1.607556	0.355456
27	6	0	-5.838278	1.476778	0.952773
28	8	0	0.643482	-0.254688	3.312441
29	6	0	1.645869	-0.859830	4.074262
30	8	0	-1.054167	2.174243	-1.447067
31	6	0	-1.001458	3.358663	-2.201520
32	1	0	5.929612	1.204093	-2.130054
33	1	0	5.238218	3.340308	-1.135495
34	1	0	3.345645	3.448085	0.435451
35	1	0	4.755038	-0.876427	-1.574615
36	1	0	1.524719	1.740624	1.771220
37	1	0	0.543065	-1.828869	2.032111
38	1	0	0.582799	-1.206960	-1.589791
39	1	0	1.775741	-3.836278	-1.416462
40	1	0	2.456766	-2.416552	-2.160341
41	1	0	3.549304	-2.722191	-0.047401

42	1	0	2.063102	-2.943216	0.809038
43	1	0	-1.627205	-0.845479	2.143568
44	1	0	-0.976091	0.743873	1.932450
45	1	0	-3.266187	-0.903405	-1.826589
46	1	0	-1.702945	-0.883827	-2.581881
47	1	0	-0.123954	-2.739742	0.401118
48	1	0	-0.874488	-3.589744	-0.897045
49	1	0	-2.972268	2.648185	-0.918695
50	1	0	-3.274692	-2.589109	-3.538842
51	1	0	-1.801353	-3.323612	-2.944052
52	1	0	-3.285515	-3.435789	-2.004663
53	1	0	-6.350295	0.605880	0.570220
54	1	0	-5.749547	1.394047	2.026175
55	1	0	-6.378548	2.373391	0.690186
56	1	0	1.634459	-0.389175	5.047833
57	1	0	1.456592	-1.923915	4.196847
58	1	0	2.627471	-0.729919	3.628856
59	1	0	-0.005011	3.419908	-2.610562
60	1	0	-1.722460	3.335670	-3.011353
61	1	0	-1.187278	4.225905	-1.576800

Standard orientation of **2c** at B3LYP/6-31G(d) level in gas phase:

Center Number	Atomic Number	Atomic Type	Coordinates (Angstroms) X	Y	Z
1	6	0	-5.121077	1.385039	0.850606
2	6	0	-4.807695	2.260347	-0.199830
3	6	0	-3.747649	2.014321	-1.042039
4	6	0	-2.995921	0.863593	-0.813268
5	6	0	-3.294026	-0.027120	0.224981
6	6	0	-4.375180	0.249171	1.067609
7	7	0	-1.900969	0.374852	-1.471939
8	6	0	-1.520342	-0.818978	-0.899114
9	6	0	-0.345844	-1.562105	-1.479361
10	7	0	-0.291244	-1.291466	2.329324
11	6	0	-1.558475	-1.983528	2.418681
12	6	0	-2.257101	-2.293414	1.074315
13	6	0	-2.334900	-1.111722	0.144757
14	6	0	1.014647	-0.847817	-1.342289
15	6	0	1.411079	-0.130385	-0.022335
16	6	0	2.452718	0.935633	-0.369916
17	6	0	2.469231	-0.227072	2.317900

18	6	0	1.885601	-1.049722	1.148917
19	6	0	0.778846	-1.994963	1.642902
20	6	0	3.647498	0.692610	-0.900268
21	1	0	0.543173	0.414510	0.314803
22	6	0	2.906429	-1.055999	3.527007
23	1	0	2.679771	-1.684634	0.764564
24	6	0	2.186002	2.380910	-0.146526
25	8	0	2.937567	3.267746	-0.430332
26	8	0	1.006618	2.612013	0.411236
27	6	0	0.666024	3.962549	0.663583
28	8	0	-0.511896	-1.716714	-2.871769
29	6	0	-1.467323	-2.658331	-3.262003
30	8	0	4.099657	-0.533999	-1.162833
31	6	0	5.371333	-0.623895	-1.757571
32	1	0	-5.955791	1.608348	1.490712
33	1	0	-5.408050	3.140319	-0.348658
34	1	0	-3.507999	2.687028	-1.846713
35	1	0	-4.626155	-0.416918	1.874376
36	1	0	-1.556507	0.698735	-2.345627
37	1	0	-0.309794	-2.544250	-1.028099
38	1	0	-0.444349	-0.393405	1.921102
39	1	0	-1.412177	-2.913573	2.961433
40	1	0	-2.216826	-1.370443	3.025509
41	1	0	-3.261112	-2.651962	1.289153
42	1	0	-1.753516	-3.116284	0.579318
43	1	0	1.779027	-1.560315	-1.627260
44	1	0	1.008726	-0.093777	-2.120836
45	1	0	3.328998	0.324613	1.957948
46	1	0	1.744239	0.513081	2.643253
47	1	0	0.424398	-2.586345	0.805817
48	1	0	1.211770	-2.710584	2.334625
49	1	0	4.311251	1.505273	-1.132108
50	1	0	3.435921	-0.428573	4.238004
51	1	0	2.059796	-1.492240	4.042631
52	1	0	3.580096	-1.858506	3.235556
53	1	0	-0.322171	3.938809	1.095585
54	1	0	1.368534	4.407842	1.352970
55	1	0	0.661290	4.530640	-0.255236
56	1	0	-1.477435	-2.672768	-4.343273
57	1	0	-1.209853	-3.651387	-2.900144
58	1	0	-2.458860	-2.403005	-2.901311
59	1	0	5.580796	-1.674764	-1.884555
60	1	0	5.381022	-0.135699	-2.725553
61	1	0	6.128458	-0.180567	-1.120519

Standard orientation of **2d** at B3LYP/6-31G(d) level in gas phase:

Center Number	Atomic Number	Atomic Type	Coordinates (Angstroms) X	Y	Z
1	6	0	5.255871	-1.316145	-0.172680
2	6	0	5.187823	-1.016907	-1.541110
3	6	0	4.174157	-0.238319	-2.050639
4	6	0	3.216946	0.239616	-1.158320
5	6	0	3.263406	-0.046057	0.211817
6	6	0	4.306491	-0.838802	0.701949
7	7	0	2.118093	1.024217	-1.376603
8	6	0	1.491392	1.274719	-0.176296
9	6	0	0.257956	2.143677	-0.183947
10	7	0	0.261182	-1.486481	2.136453
11	6	0	1.310777	-0.774416	2.826521
12	6	0	1.780517	0.600529	2.290629
13	6	0	2.137445	0.631761	0.828004
14	6	0	-1.009378	1.439648	-0.698185
15	6	0	-1.296511	0.073753	-0.045253
16	6	0	-2.321706	-0.745872	-0.828034
17	6	0	-3.047037	0.525560	1.819354
18	6	0	-1.583619	0.183514	1.484817
19	6	0	-1.120008	-1.072492	2.256555
20	6	0	-3.432712	-0.300720	-1.413007
21	1	0	-0.374191	-0.472966	-0.142255
22	6	0	-3.256537	1.084957	3.230084
23	1	0	-0.986501	1.010117	1.850217
24	6	0	-2.109071	-2.202881	-1.055765
25	8	0	-2.887112	-2.934171	-1.595439
26	8	0	-0.940090	-2.639111	-0.606484
27	6	0	-0.657980	-4.017366	-0.776201
28	8	0	0.448653	3.244757	-1.040761
29	6	0	1.275827	4.254658	-0.541385
30	8	0	-3.854106	0.961641	-1.381633
31	6	0	-5.026686	1.251928	-2.103226
32	1	0	6.062841	-1.925274	0.193673
33	1	0	5.942732	-1.402574	-2.203135
34	1	0	4.122884	-0.007744	-3.100247
35	1	0	4.374734	-1.070525	1.749894
36	1	0	1.904712	1.512615	-2.214872
37	1	0	0.092367	2.509222	0.822770

38	1	0	0.511812	-1.720344	1.200550
39	1	0	1.004120	-0.642757	3.860862
40	1	0	2.171446	-1.435217	2.850446
41	1	0	2.654132	0.884252	2.876254
42	1	0	1.030781	1.353702	2.500818
43	1	0	-1.830318	2.132317	-0.573943
44	1	0	-0.885025	1.297516	-1.766790
45	1	0	-3.413922	1.256698	1.108746
46	1	0	-3.666947	-0.357947	1.689205
47	1	0	-1.315106	-0.924412	3.313332
48	1	0	-1.737217	-1.913171	1.953340
49	1	0	-4.052827	-0.988954	-1.957387
50	1	0	-4.296943	1.358336	3.379247
51	1	0	-2.998005	0.371375	4.004741
52	1	0	-2.658236	1.978147	3.390852
53	1	0	0.312138	-4.174046	-0.331788
54	1	0	-1.401853	-4.619795	-0.276432
55	1	0	-0.637735	-4.271636	-1.825698
56	1	0	1.330500	5.026128	-1.297272
57	1	0	0.863464	4.683354	0.369030
58	1	0	2.277269	3.891121	-0.332160
59	1	0	-5.222341	2.304050	-1.965524
60	1	0	-4.891832	1.046302	-3.159047
61	1	0	-5.864812	0.676969	-1.725933

Standard orientation of **3a** at B3LYP/6-31G(d) level in gas phase:

Center Number	Atomic Number	Atomic Type	Coordinates (Angstroms) X	Y	Z
1	6	0	-5.556798	-0.542559	-1.275253
2	6	0	-5.423848	-1.846822	-0.778428
3	6	0	-4.365782	-2.197784	0.029392
4	6	0	-3.431721	-1.210601	0.334614
5	6	0	-3.548427	0.100745	-0.142530
6	6	0	-4.630715	0.427987	-0.964524
7	7	0	-2.294904	-1.267566	1.090139
8	6	0	-1.703352	-0.022778	1.126329
9	6	0	-0.436004	0.128388	1.924575
10	7	0	0.116436	2.498710	-0.916032
11	6	0	-1.317103	2.520156	-1.182495
12	6	0	-2.163489	2.302446	0.086047
13	6	0	-2.429368	0.852895	0.392403

14	6	0	0.768031	-0.641659	1.356339
15	6	0	1.514688	0.083545	0.219048
16	6	0	2.949012	-0.416825	0.063013
17	6	0	4.001249	0.388960	0.175636
18	6	0	0.239726	-0.878467	-1.725976
19	6	0	0.811228	0.164299	-1.144042
20	6	0	0.880880	1.560142	-1.728937
21	6	0	0.690480	3.824028	-0.852783
22	6	0	-0.464329	-0.949799	-3.054847
23	6	0	3.209910	-1.850886	-0.211707
24	8	0	2.379487	-2.706418	-0.193779
25	8	0	4.488171	-2.125079	-0.474091
26	6	0	4.811792	-3.481677	-0.712470
27	8	0	-0.634498	-0.395308	3.221057
28	6	0	-1.471569	0.353936	4.049980
29	8	0	3.896341	1.697389	0.433689
30	6	0	5.099954	2.396029	0.628040
31	1	0	-6.394004	-0.301809	-1.906043
32	1	0	-6.161573	-2.586476	-1.034974
33	1	0	-4.262628	-3.199767	0.407509
34	1	0	-4.745301	1.425693	-1.350984
35	1	0	-2.019574	-2.022190	1.674417
36	1	0	-0.189801	1.183597	1.994005
37	1	0	-1.567276	1.751497	-1.902784
38	1	0	-1.589982	3.471027	-1.640157
39	1	0	-1.672412	2.793787	0.919396
40	1	0	-3.121574	2.800924	-0.038771
41	1	0	0.469464	-1.638930	1.069713
42	1	0	1.457925	-0.750875	2.186099
43	1	0	1.616117	1.105812	0.552143
44	1	0	5.002238	0.019440	0.058749
45	1	0	0.261121	-1.816343	-1.200767
46	1	0	1.917945	1.878597	-1.709354
47	1	0	0.573199	1.585004	-2.771049
48	1	0	1.711261	3.768088	-0.493646
49	1	0	0.128041	4.433952	-0.153836
50	1	0	0.693310	4.341541	-1.817196
51	1	0	-0.465473	-0.016297	-3.601251
52	1	0	0.002474	-1.702486	-3.685801
53	1	0	-1.497765	-1.253896	-2.911917
54	1	0	5.874427	-3.501498	-0.900513
55	1	0	4.572941	-4.088252	0.149106
56	1	0	4.273207	-3.855859	-1.570758
57	1	0	-1.504922	-0.148203	5.007497

58	1	0	-1.081708	1.359497	4.194496
59	1	0	-2.479118	0.426859	3.652820
60	1	0	4.835688	3.426656	0.808430
61	1	0	5.639695	2.009562	1.485648
62	1	0	5.730660	2.336481	-0.252521

Standard orientation of **3b** at B3LYP/6-31G(d) level in gas phase:

Center Number	Atomic Number	Atomic Type	Coordinates (Angstroms) X	Y	Z
1	6	0	-5.556826	-0.542718	-1.275098
2	6	0	-5.423932	-1.846891	-0.778010
3	6	0	-4.365900	-2.197727	0.029908
4	6	0	-3.431791	-1.210520	0.334948
5	6	0	-3.548440	0.100732	-0.142470
6	6	0	-4.630711	0.427852	-0.964543
7	7	0	-2.295016	-1.267352	1.090539
8	6	0	-1.703352	-0.022604	1.126405
9	6	0	-0.435923	0.128640	1.924503
10	7	0	0.116346	2.498430	-0.916569
11	6	0	-1.317208	2.519878	-1.182914
12	6	0	-2.163468	2.302454	0.085766
13	6	0	-2.429370	0.852951	0.392341
14	6	0	0.768067	-0.641492	1.356234
15	6	0	1.514670	0.083581	0.218823
16	6	0	2.949034	-0.416702	0.062872
17	6	0	4.001210	0.389203	0.175222
18	6	0	0.239808	-0.878874	-1.726059
19	6	0	0.811225	0.164036	-1.144293
20	6	0	0.880747	1.559793	-1.729416
21	6	0	0.690426	3.823722	-0.853240
22	6	0	-0.464217	-0.950492	-3.054931
23	6	0	3.210008	-1.850834	-0.211482
24	8	0	2.379673	-2.706436	-0.193046
25	8	0	4.488241	-2.124963	-0.474115
26	6	0	4.812004	-3.481592	-0.712140
27	8	0	-0.634298	-0.394828	3.221097
28	6	0	-1.471440	0.354460	4.049926
29	8	0	3.896205	1.697728	0.432793
30	6	0	5.099663	2.396095	0.629116
31	1	0	-6.394019	-0.302066	-1.905942
32	1	0	-6.161680	-2.586566	-1.034426

33	1	0	-4.262799	-3.199633	0.408236
34	1	0	-4.745260	1.425492	-1.351181
35	1	0	-2.019503	-2.022005	1.674686
36	1	0	-0.189715	1.183855	1.993736
37	1	0	-1.567460	1.751048	-1.902990
38	1	0	-1.590104	3.470646	-1.640784
39	1	0	-1.672223	2.793861	0.918971
40	1	0	-3.121534	2.800977	-0.038981
41	1	0	0.469477	-1.638789	1.069727
42	1	0	1.458010	-0.750626	2.185959
43	1	0	1.616034	1.105908	0.551735
44	1	0	5.002228	0.019752	0.058361
45	1	0	0.261220	-1.816652	-1.200671
46	1	0	1.917796	1.878298	-1.709984
47	1	0	0.572956	1.584465	-2.771500
48	1	0	1.711207	3.767753	-0.494113
49	1	0	0.127997	4.433628	-0.154270
50	1	0	0.693259	4.341291	-1.817623
51	1	0	-0.465056	-0.017192	-3.601685
52	1	0	0.002387	-1.703550	-3.685581
53	1	0	-1.497748	-1.254231	-2.911929
54	1	0	5.874582	-3.501313	-0.900508
55	1	0	4.573514	-4.087920	0.149705
56	1	0	4.273211	-3.856148	-1.570130
57	1	0	-1.504460	-0.147335	5.007633
58	1	0	-1.081856	1.360188	4.193991
59	1	0	-2.479079	0.426934	3.652922
60	1	0	4.835400	3.426928	0.808323
61	1	0	5.637550	2.009973	1.488036
62	1	0	5.732105	2.335765	-0.250143

Standard orientation of **3c** at B3LYP/6-31G(d) level in gas phase:

Center Number	Atomic Number	Atomic Type	Coordinates (Angstroms) X	Y	Z
1	6	0	-5.508718	-0.164382	-1.208967
2	6	0	-5.465084	-1.482437	-0.733932
3	6	0	-4.424506	-1.922708	0.053047
4	6	0	-3.417131	-1.011383	0.359784
5	6	0	-3.445195	0.312789	-0.093746
6	6	0	-4.510489	0.731304	-0.895952
7	7	0	-2.274756	-1.165088	1.095121

8	6	0	-1.599743	0.037359	1.150218
9	6	0	-0.315119	0.078604	1.934161
10	7	0	0.346629	2.498279	-0.911944
11	6	0	-1.090781	2.602526	-1.133208
12	6	0	-1.910009	2.409182	0.158020
13	6	0	-2.269424	0.974866	0.440424
14	6	0	0.834657	-0.734040	1.314137
15	6	0	1.591177	-0.009014	0.183140
16	6	0	3.018206	-0.521829	-0.002300
17	6	0	4.081450	0.262847	0.154328
18	6	0	0.215632	-0.863872	-1.748521
19	6	0	0.875183	0.126553	-1.168891
20	6	0	1.032683	1.519055	-1.745400
21	6	0	0.999318	3.787445	-0.861612
22	6	0	-0.512021	-0.873776	-3.066725
23	6	0	3.369238	-1.915919	-0.379407
24	8	0	4.464940	-2.281661	-0.690223
25	8	0	2.345525	-2.753523	-0.314873
26	6	0	2.599088	-4.102126	-0.660552
27	8	0	-0.529107	-0.490162	3.208838
28	6	0	-1.292752	0.280078	4.088867
29	8	0	4.006320	1.552315	0.494620
30	6	0	5.227646	2.213418	0.715708
31	1	0	-6.334111	0.146912	-1.824164
32	1	0	-6.258426	-2.161954	-0.990713
33	1	0	-4.390351	-2.935528	0.414607
34	1	0	-4.557162	1.741011	-1.264852
35	1	0	-2.069085	-1.929839	1.694933
36	1	0	-0.004384	1.112911	2.046312
37	1	0	-1.405306	1.863622	-1.859568
38	1	0	-1.323970	3.575487	-1.564770
39	1	0	-1.358503	2.842779	0.985391
40	1	0	-2.834296	2.976049	0.078099
41	1	0	0.471474	-1.703889	1.006935
42	1	0	1.535717	-0.906958	2.123101
43	1	0	1.718436	1.003335	0.536984
44	1	0	5.070352	-0.129183	0.002970
45	1	0	0.164247	-1.802198	-1.225751
46	1	0	2.089072	1.765719	-1.739292
47	1	0	0.711718	1.573567	-2.782347
48	1	0	2.024676	3.669361	-0.532567
49	1	0	0.494170	4.425697	-0.144467
50	1	0	1.004289	4.308332	-1.824065
51	1	0	-0.445886	0.059150	-3.609544

52	1	0	-0.115846	-1.657316	-3.708489
53	1	0	-1.564345	-1.094696	-2.909586
54	1	0	1.654521	-4.614003	-0.558850
55	1	0	2.956250	-4.172886	-1.677529
56	1	0	3.332456	-4.533425	0.005016
57	1	0	-1.352497	-0.265321	5.021002
58	1	0	-0.822158	1.243053	4.275079
59	1	0	-2.296704	0.451592	3.713316
60	1	0	4.986944	3.235512	0.964664
61	1	0	5.769458	1.761631	1.539053
62	1	0	5.844870	2.196157	-0.175766

Standard orientation of **3d** at B3LYP/6-31G(d) level in gas phase:

Center Number	Atomic Number	Atomic Type	Coordinates (Angstroms) X	Y	Z
1	6	0	5.574577	-0.027975	-1.200343
2	6	0	5.555371	1.322862	-0.826603
3	6	0	4.516235	1.843637	-0.088433
4	6	0	3.485457	0.980016	0.273545
5	6	0	3.488839	-0.375126	-0.078280
6	6	0	4.553188	-0.875736	-0.833323
7	7	0	2.338538	1.213932	0.980130
8	6	0	1.635598	0.034233	1.116741
9	6	0	0.345881	0.074028	1.891625
10	7	0	-0.385546	-2.495532	-0.681656
11	6	0	1.040046	-2.671789	-0.933302
12	6	0	1.901454	-2.411987	0.316217
13	6	0	2.292834	-0.968837	0.490008
14	6	0	-0.772892	0.898506	1.231538
15	6	0	-1.590802	0.149684	0.159075
16	6	0	-2.950621	0.815842	-0.073752
17	6	0	-3.150228	2.095651	-0.381204
18	6	0	-0.195725	0.760762	-1.842423
19	6	0	-0.877716	-0.152160	-1.169349
20	6	0	-1.063943	-1.591788	-1.603376
21	6	0	-1.071415	-3.752633	-0.480598
22	6	0	0.534995	0.623178	-3.151785
23	6	0	-4.218469	0.048631	0.044241
24	8	0	-5.312744	0.499186	-0.133552
25	8	0	-4.038779	-1.224136	0.379124
26	6	0	-5.204591	-2.012870	0.528665

27	8	0	0.573671	0.693554	3.140860
28	6	0	1.312151	-0.057308	4.058026
29	8	0	-2.175194	2.988990	-0.530301
30	6	0	-2.566052	4.297959	-0.866259
31	1	0	6.399449	-0.402657	-1.779876
32	1	0	6.366607	1.963735	-1.123706
33	1	0	4.500694	2.881440	0.195211
34	1	0	4.581391	-1.911140	-1.124659
35	1	0	2.145265	2.025977	1.518751
36	1	0	-0.000806	-0.942614	2.048265
37	1	0	1.358654	-1.999603	-1.719726
38	1	0	1.225994	-3.682099	-1.297916
39	1	0	1.369363	-2.778844	1.187625
40	1	0	2.812396	-3.001042	0.246873
41	1	0	-0.369605	1.828061	0.855115
42	1	0	-1.450732	1.158105	2.036556
43	1	0	-1.809909	-0.812106	0.594578
44	1	0	-4.151129	2.461054	-0.519294
45	1	0	-0.129841	1.747011	-1.417891
46	1	0	-2.123496	-1.819020	-1.566790
47	1	0	-0.751519	-1.754665	-2.631430
48	1	0	-2.083595	-3.571501	-0.139656
49	1	0	-0.563572	-4.328591	0.285606
50	1	0	-1.118041	-4.369184	-1.383419
51	1	0	0.450019	-0.357970	-3.598659
52	1	0	0.156936	1.346355	-3.870834
53	1	0	1.591679	0.836350	-3.013922
54	1	0	-4.860027	-2.996604	0.807965
55	1	0	-5.841690	-1.606200	1.300225
56	1	0	-5.755390	-2.057382	-0.399505
57	1	0	1.381731	0.526625	4.965858
58	1	0	0.815203	-0.998498	4.283423
59	1	0	2.313072	-0.271585	3.696720
60	1	0	-1.661696	4.881579	-0.943324
61	1	0	-3.086347	4.313900	-1.817180
62	1	0	-3.202655	4.720885	-0.097252

Standard orientation of **4a** at B3LYP/6-31G(d) level in gas phase:

Center Number	Atomic Number	Atomic Type	Coordinates (Angstroms) X	Y	Z
1	6	0	-5.155985	2.494411	-0.409187

2	6	0	-6.133396	1.995946	0.434286
3	6	0	-5.992148	0.757163	1.053905
4	6	0	-4.842603	0.045367	0.793436
5	6	0	-3.858292	0.524268	-0.060308
6	6	0	-4.003951	1.752061	-0.660224
7	7	0	-4.451329	-1.199414	1.292689
8	6	0	-3.204948	-1.557365	0.866833
9	6	0	-2.736685	-0.486953	-0.155456
10	8	0	-1.540934	0.118895	0.242213
11	6	0	-0.340004	-0.601412	0.088359
12	7	0	-0.182114	-1.050483	-1.278937
13	6	0	-1.299378	-1.883661	-1.688133
14	6	0	-2.590825	-1.086267	-1.559880
15	6	0	0.773399	0.357740	0.462936
16	6	0	2.155657	-0.281305	0.260801
17	6	0	2.288336	-0.813722	-1.184628
18	6	0	1.090757	-1.723266	-1.477676
19	8	0	-2.611414	-2.531732	1.218520
20	6	0	3.277105	0.630691	0.733736
21	6	0	3.617355	-1.552181	-1.405823
22	6	0	3.864664	-2.015193	-2.844045
23	6	0	4.043335	0.330425	1.780647
24	8	0	3.919379	-0.795172	2.481919
25	6	0	4.793686	-0.960202	3.571138
26	6	0	3.606142	1.940670	0.116018
27	8	0	4.464017	2.681024	0.500046
28	8	0	2.850112	2.230722	-0.932941
29	6	0	3.092824	3.466832	-1.577995
30	1	0	-5.284457	3.457463	-0.868476
31	1	0	-7.019691	2.575602	0.621307
32	1	0	-6.751267	0.378393	1.714329
33	1	0	-3.234666	2.137793	-1.304666
34	1	0	-4.924674	-1.710400	2.003549
35	1	0	-0.354898	-1.450357	0.771005
36	1	0	-1.157684	-2.164851	-2.726133
37	1	0	-1.356495	-2.802128	-1.103342
38	1	0	-2.562124	-0.263903	-2.265522
39	1	0	-3.453658	-1.700409	-1.798740
40	1	0	0.643826	0.647470	1.500499
41	1	0	0.664726	1.243335	-0.148314
42	1	0	2.202433	-1.148632	0.911731
43	1	0	2.235225	0.023281	-1.871256
44	1	0	1.120921	-2.047434	-2.510357
45	1	0	1.162409	-2.626890	-0.860209

46	1	0	4.430325	-0.894382	-1.117149
47	1	0	3.671363	-2.408152	-0.735322
48	1	0	4.868633	-2.415775	-2.946598
49	1	0	3.175445	-2.793744	-3.153189
50	1	0	3.767327	-1.189126	-3.543340
51	1	0	4.811100	1.010060	2.102283
52	1	0	4.557646	-1.914716	4.015496
53	1	0	5.826803	-0.962244	3.241820
54	1	0	4.649411	-0.176040	4.305886
55	1	0	2.384721	3.516833	-2.390514
56	1	0	2.938249	4.288645	-0.894177
57	1	0	4.103580	3.505400	-1.957359

Standard orientation of **4b** at B3LYP/6-31G(d) level in gas phase:

Center Number	Atomic Number	Atomic Type	Coordinates (Angstroms) X	Y	Z
1	6	0	-5.155826	2.494667	-0.408893
2	6	0	-6.133344	1.996099	0.434404
3	6	0	-5.992218	0.757197	1.053799
4	6	0	-4.842685	0.045383	0.793294
5	6	0	-3.858275	0.524387	-0.060262
6	6	0	-4.003814	1.752307	-0.659962
7	7	0	-4.451531	-1.199517	1.292346
8	6	0	-3.205092	-1.557416	0.866593
9	6	0	-2.736709	-0.486868	-0.155499
10	8	0	-1.540938	0.118880	0.242279
11	6	0	-0.340027	-0.601441	0.088342
12	7	0	-0.182120	-1.050334	-1.279014
13	6	0	-1.299408	-1.883399	-1.688356
14	6	0	-2.590832	-1.085975	-1.560014
15	6	0	0.773411	0.357620	0.463069
16	6	0	2.155671	-0.281400	0.260824
17	6	0	2.288327	-0.813661	-1.184660
18	6	0	1.090721	-1.723149	-1.477784
19	8	0	-2.611619	-2.531841	1.218224
20	6	0	3.277128	0.630550	0.733845
21	6	0	3.617308	-1.552145	-1.405952
22	6	0	3.864521	-2.015044	-2.844229
23	6	0	4.042965	0.330443	1.781090
24	8	0	3.918439	-0.794826	2.482771
25	6	0	4.793450	-0.960432	3.571331

26	6	0	3.606616	1.940281	0.115875
27	8	0	4.465044	2.680211	0.499511
28	8	0	2.850267	2.230704	-0.932748
29	6	0	3.093281	3.466708	-1.577896
30	1	0	-5.284210	3.457815	-0.868008
31	1	0	-7.019622	2.575774	0.621451
32	1	0	-6.751417	0.378340	1.714082
33	1	0	-3.234441	2.138112	-1.304256
34	1	0	-4.924922	-1.710553	2.003140
35	1	0	-0.354949	-1.450485	0.770864
36	1	0	-1.157705	-2.164462	-2.726392
37	1	0	-1.356582	-2.801951	-1.103700
38	1	0	-2.562083	-0.263499	-2.265525
39	1	0	-3.453680	-1.700049	-1.798993
40	1	0	0.643870	0.647179	1.500685
41	1	0	0.664751	1.243325	-0.148021
42	1	0	2.202458	-1.148811	0.911647
43	1	0	2.235227	0.023399	-1.871220
44	1	0	1.120894	-2.047268	-2.510484
45	1	0	1.162345	-2.626815	-0.860370
46	1	0	4.430329	-0.894427	-1.117229
47	1	0	3.671282	-2.408192	-0.735540
48	1	0	4.868472	-2.415645	-2.946887
49	1	0	3.175259	-2.793552	-3.153392
50	1	0	3.767156	-1.188915	-3.543446
51	1	0	4.810818	1.009992	2.102702
52	1	0	4.555453	-1.913677	4.017367
53	1	0	5.826212	-0.965583	3.240929
54	1	0	4.651939	-0.174817	4.305067
55	1	0	2.384289	3.517425	-2.389595
56	1	0	2.940223	4.288560	-0.893792
57	1	0	4.103631	3.504448	-1.958446

Standard orientation of **5a** at B3LYP/6-31G(d) level in gas phase:

Center Number	Atomic Number	Atomic Type	Coordinates (Angstroms) X	Y	Z
1	6	0	-4.696233	-1.187439	2.544629
2	6	0	-5.693351	-1.715459	1.743122
3	6	0	-5.649222	-1.593285	0.357008
4	6	0	-4.575352	-0.924584	-0.186531
5	6	0	-3.573231	-0.377478	0.603102

6	6	0	-3.622338	-0.511083	1.970095
7	7	0	-4.286786	-0.678534	-1.531255
8	6	0	-3.092620	-0.040058	-1.694169
9	6	0	-2.549352	0.300474	-0.280363
10	8	0	-2.585223	0.217287	-2.743794
11	8	0	-1.296564	-0.281004	-0.054664
12	6	0	-0.171271	0.285834	-0.678893
13	7	0	-0.068116	1.701798	-0.366537
14	6	0	-1.268636	2.416298	-0.771626
15	6	0	-2.475130	1.816187	-0.061805
16	6	0	1.027919	-0.469099	-0.133962
17	6	0	2.360402	0.120750	-0.622565
18	6	0	2.402595	1.646079	-0.425938
19	6	0	1.121462	2.273175	-0.973721
20	6	0	3.553558	-0.496542	0.066853
21	6	0	4.231448	0.178055	0.992695
22	8	0	3.932161	1.393441	1.432288
23	6	0	2.684868	1.978057	1.042355
24	6	0	2.816932	3.456989	1.357294
25	6	0	3.982500	-1.842894	-0.347813
26	8	0	3.448227	-2.470245	-1.211770
27	8	0	5.030327	-2.308568	0.327292
28	6	0	5.497602	-3.595626	-0.029325
29	1	0	-4.748633	-1.302981	3.611726
30	1	0	-6.518761	-2.235411	2.195598
31	1	0	-6.422691	-2.012363	-0.260940
32	1	0	-2.837789	-0.109359	2.585752
33	1	0	-4.789303	-1.048457	-2.306661
34	1	0	-0.256480	0.143064	-1.754954
35	1	0	-1.164805	3.455982	-0.479958
36	1	0	-1.408404	2.389214	-1.851979
37	1	0	-2.374920	1.989462	1.003671
38	1	0	-3.393998	2.290825	-0.391366
39	1	0	0.956036	-1.507432	-0.432128
40	1	0	0.967902	-0.435320	0.947669
41	1	0	2.445038	-0.081892	-1.685829
42	1	0	3.235902	2.041714	-1.003225
43	1	0	1.112539	3.341793	-0.799040
44	1	0	1.113412	2.138654	-2.059895
45	1	0	5.111135	-0.211912	1.464401
46	1	0	1.919764	1.544490	1.673095
47	1	0	1.859979	3.957294	1.273389
48	1	0	3.174054	3.581217	2.372776
49	1	0	3.524255	3.935288	0.687454

50	1	0	4.725600	-4.336045	0.122029
51	1	0	5.810246	-3.614671	-1.063330
52	1	0	6.337514	-3.793405	0.618805

Standard orientation of **5b** at B3LYP/6-31G(d) level in gas phase:

Center Number	Atomic Number	Atomic Type	Coordinates (Angstroms) X	Y	Z
1	6	0	4.728688	0.559314	2.638318
2	6	0	5.720911	1.199250	1.916147
3	6	0	5.634569	1.339708	0.533733
4	6	0	4.523894	0.815249	-0.088342
5	6	0	3.525836	0.158863	0.618703
6	6	0	3.617032	0.032805	1.984321
7	7	0	4.189376	0.834272	-1.445125
8	6	0	2.968856	0.274572	-1.682988
9	6	0	2.456933	-0.314616	-0.341246
10	8	0	2.419918	0.239799	-2.742878
11	8	0	1.228250	0.242665	0.031359
12	6	0	0.069511	-0.172922	-0.647958
13	7	0	-0.064670	-1.618947	-0.602922
14	6	0	1.102096	-2.270854	-1.176878
15	6	0	2.345747	-1.842428	-0.409338
16	6	0	-1.091385	0.492604	0.069412
17	6	0	-2.454227	0.039650	-0.478521
18	6	0	-2.534003	-1.495699	-0.564185
19	6	0	-1.288559	-2.039157	-1.263101
20	6	0	-3.614218	0.540945	0.350516
21	6	0	-4.279512	-0.282775	1.158373
22	8	0	-4.000546	-1.562406	1.359248
23	6	0	-2.782141	-2.090129	0.825586
24	6	0	-2.944910	-3.598822	0.863174
25	6	0	-4.104647	1.930124	0.280256
26	8	0	-5.005932	2.378576	0.924253
27	8	0	-3.429660	2.658771	-0.602235
28	6	0	-3.822398	4.010170	-0.750234
29	1	0	4.814344	0.471812	3.705899
30	1	0	6.575741	1.601641	2.429644
31	1	0	6.404753	1.844547	-0.020966
32	1	0	2.836699	-0.454840	2.540371
33	1	0	4.682491	1.328888	-2.154238
34	1	0	0.126449	0.167514	-1.680845

35	1	0	0.977497	-3.344229	-1.082387
36	1	0	1.209764	-2.043394	-2.237211
37	1	0	2.272521	-2.211460	0.607342
38	1	0	3.240590	-2.266531	-0.853789
39	1	0	-0.992061	1.566759	-0.026154
40	1	0	-1.004025	0.252547	1.122427
41	1	0	-2.556378	0.428870	-1.486876
42	1	0	-3.395019	-1.757651	-1.175739
43	1	0	-1.305820	-3.121406	-1.290036
44	1	0	-1.308955	-1.705047	-2.305290
45	1	0	-5.134705	0.042846	1.717392
46	1	0	-1.987361	-1.799169	1.500021
47	1	0	-2.004538	-4.095129	0.656930
48	1	0	-3.277092	-3.902614	1.848762
49	1	0	-3.683388	-3.928236	0.139388
50	1	0	-4.849190	4.073300	-1.080229
51	1	0	-3.716815	4.541141	0.184659
52	1	0	-3.163123	4.428242	-1.495216

----

(1) Goto, H.; Osawa, E. *J. Am. Chem. Soc.* **1989**, *111*, 8950–8951.

(2) Goto, H.; Osawa, E. *J. Chem. Soc., Perkin Trans.* **1993**, *2*, 187–198.

(3) Frisch, M. J.; Trucks, G. W.; Schlegel, H. B.; Scuseria, G. E.; Robb, M.A.; Cheeseman, J. R.; Scalmani, G.; Barone, V.; Mennucci, B.; Petersson, G. A.; Nakatsuji, H.; Caricato, M.; Li, X.; Hratchian, H. P.; Izmaylov, A. F.; Bloino, J.; Zheng, G.; Sonnenberg, J. L.; Hada, M.; Ehara,M.; Toyota, K.; Fukuda, R.;Hasegawa, J.; Ishida, M.;Nakajima, T.; Honda, Y.; Kitao, O.; Nakai, H.; Vreven, T.; Montgomery, Jr., J. A.; Peralta, J. E.; Ogliaro, F.; Bearpark,M.; Heyd, J. J.; Brothers, E.; Kudin, K. N.; Staroverov, V. N.; Keith, T.; Kobayashi, R.; Normand, J.; Raghavachari, K.; Rendell, A.; Burant, J. C.; Iyengar, S. S.; Tomasi, J.; Cossi, M.; Rega, N.; Millam, J. M.; Klene, M.; Knox, J. E.; Cross, J. B.; Bakken, V.; Adamo, C.; Jaramillo, J.; Gomperts, R.; Stratmann, R. E.; Yazyev,O.; Austin,A. J.; Cammi, R.; Pomelli,C.; Ochterski, J.W.; Martin,R. L.;Morokuma, K.; Zakrzewski,V. G.;Voth, G. A.; Salvador, P.; Dannenberg, J. J.; Dapprich, S.;Daniels, A. D.; Farkas, O.; Foresman, J. B.; Ortiz, J. V.; Cioslowski, J.; Fox, D. J. Gaussian 09, revision C.01; Gaussian, Inc.: Wallingford, CT, 2010.

(4) Bruhn, T.; Schaumlöffel, A.; Hemberger, Y.; Bringmann, G. *SpecDis*, version 1.60, University of Wuerzburg, Germany, 2012.
